# Comparative Efficacy of Pharmacological Therapies for Low Back Pain: A Bayesian Network Analysis

**DOI:** 10.3389/fphar.2022.811962

**Published:** 2022-02-15

**Authors:** Jiuzhou Jiang, Hao Pan, Haomai Chen, Liyang Song, Yiyun Wang, Bao Qian, Pengfei Chen, Shunwu Fan, Xianfeng Lin

**Affiliations:** ^1^ Department of Thoracic Surgery, Sir Run Run Shaw Hospital, Medical College of Zhejiang University, Hangzhou, China; ^2^ Department of Orthopaedics, The First Affiliated Hospital of Wenzhou Medical University, Wenzhou, China; ^3^ Department of Cardiovascular Surgery, Ruijin Hospital, School of Medicine, Shanghai Jiao Tong University, Shanghai, China; ^4^ Wenzhou Medical University, Wenzhou, China; ^5^ Department of Orthopaedic Surgery, Sir Run Run Shaw Hospital, Medical College of Zhejiang University, Hangzhou, China

**Keywords:** low back pain, bayesian network analysis, meta analyses, pharmacological therapies, drug combination

## Abstract

Low back pain (LBP) is a common problem, but the efficacy of pharmacological therapies remains controversial. Therefore, we aimed to comprehensively evaluate and quantitatively rank various pharmacological therapies for patients with low back pain. Two meta-analyses were performed: an initial pair-wise meta-analysis, followed by network meta-analysis using a random-effects Bayesian model. We included randomized controlled trials comparing placebos, non-steroidal anti-inflammatory drugs, opioids, skeletal muscular relaxants, pregabalin (or gabapentin), and some drug combinations. The primary and secondary outcomes were pain intensity and physical function. Eighty-eight eligible trials with 21,377 patients were included. Here, we show that only skeletal muscle relaxants significantly decreased the pain intensity of acute (including subacute) low back pain. Several kinds of drugs significantly decreased the pain of chronic low back pain, but only opioids and cyclo-oxygenase 2-selective non-steroidal anti-inflammatory drugs effectively reduced pain and improved function. Pregabalin (or gabapentin) seemed to be an effective treatment to relieve pain, but it should be used with caution for low back pain.

## Introduction

Low back pain (LBP), with an estimated mean point prevalence of 18.3%, is one of the greatest challenges to worldwide health. (Hoy et al., 2012). Most people experience LBP, and it is the dominant cause of years lived with disability. (Global Burden of Disease Study, 2015). Systematic pharmacological therapy is one of the most important and basic choices to control LBP in many major international clinical guidelines (such as the American College of Physicians (ACP), (Qaseem et al., 2017), National Institute of Health and Care Excellence (NICE), (de Campos, 2017), and Evidence-Informed Primary Care Management of Low Back Pain (Canada) (Group. TOPTLBPW, 2015) guidelines). Various drugs are commonly used in LBP pharmacotherapy, including opioids, non-steroidal anti-inflammatory drugs (NSAIDs), skeletal muscle relaxants, antidepressants, corticosteroids, antiepileptics (pregabalin or gabapentin), and combination medications (with more than two active ingredients). (Maher et al., 2017).

Previous pair-wise meta-analyses have assessed a few of the commonly prescribed medications, and the results showed that skeletal muscle relaxants (Chou et al., 2017a) and opioids (Chaparro et al., 2013) were effective against acute (include subacute) and chronic LBP, respectively, while NSAIDs were effective for both acute (include subacute) and chronic LBP. (Roelofs et al., 2008; Enthoven et al., 2016). Nevertheless, these studies were insufficient by only considering the direct comparative evidence between two medications. Moreover, most of the studies only compared the active interventions to placebo. Therefore, multiple comparisons between various medications are still lacking. Additionally, limited by statistical methodological shortcomings, these pair-wise meta-analyses could not quantitatively rank the efficacies of numerous medications and objectively recommend the most suitable treatments for patients with LBP.

Recently, the use of network meta-analysis has gradually increased in evidence-based medicine studies and has been proven to have outstanding advantages for assessing intricate treatment efficacy in osteoarthritis, (da Costa et al., 2017), myocardial infarction, (Jinatongthai et al., 2017), diabetes, (Palmer et al., 2016), and other areas. Network meta-analysis (NMA) can synthesize all of the direct and indirect comparison evidence into one statistical framework and then comprehensively evaluate and rank the exact quantitative efficacy of the numerous treatments. (Lu and Ades, 2004). Therefore, in this study, we aimed to perform a Bayesian random-effects network meta-analysis to comprehensively evaluate and quantitatively grade the effects of various pharmacological therapies for patients with LBP.

In our analysis, the main assumptions are that the reduce of LBP and the promote of function come from medical treatements. And the curative effect of all drugs is independent of the age and gender.

## Results

### Search Results

**FIGURE 1:**
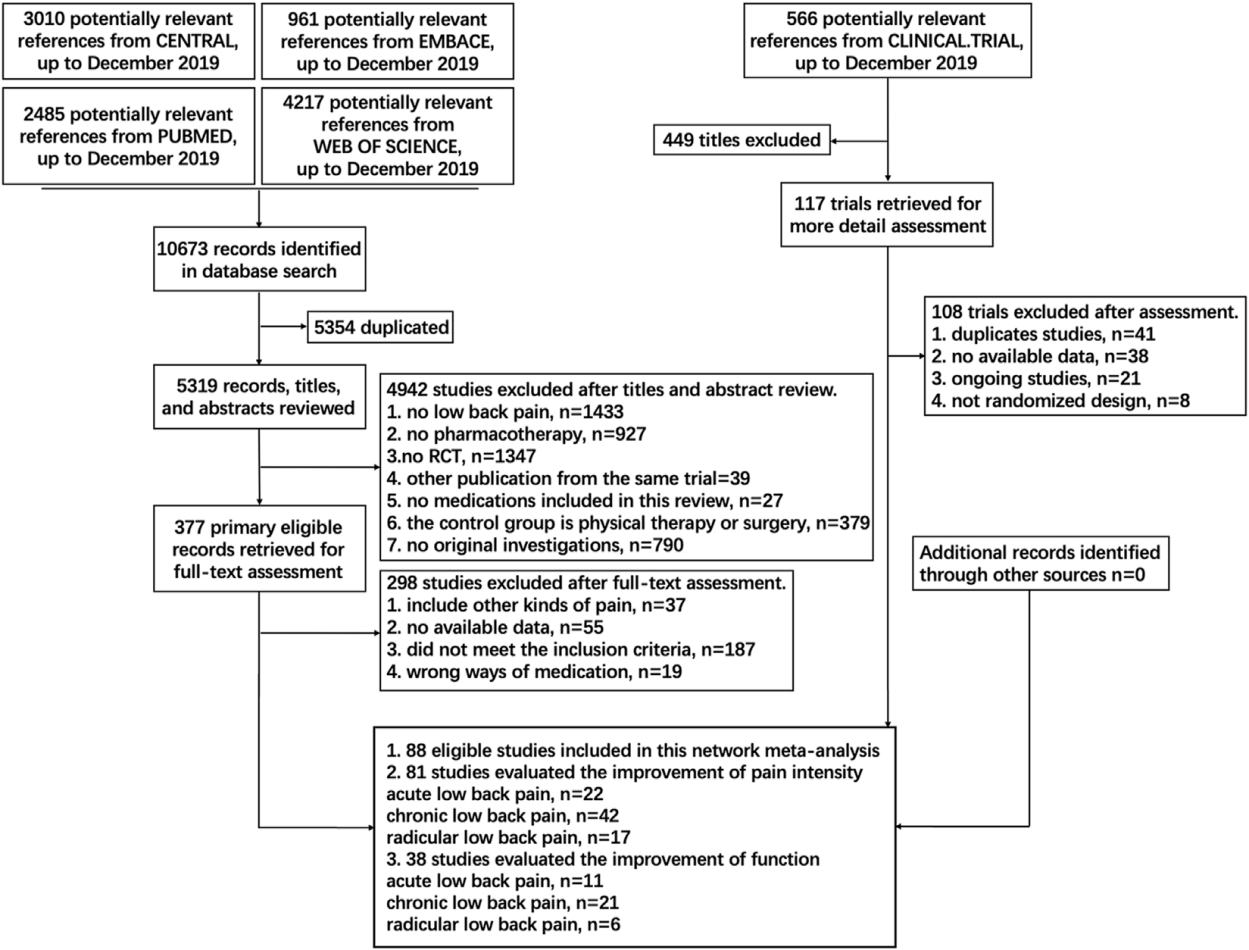
Abbreviations: PLA, Placebo; OPI, Opioids; NSA, Non-steroidal anti-inflammatory drug; TCA, Tricyclic antidepressant; TAP, Tapentadol; SSI, Selective serotonin reuptake inhibitor; GMA, GABA mimetic antiepileptics (Pregabalin or Gabapentin); ANE, Antiepileptic (apart from Pregabalin and Gabapentin); TRA, Tramadol; DUL, Duloxetine; C-NSA, Cyclo-oxygenase 2-selective non-steroidal anti-inflammatory drugs; ACE, Acetaminophen; TAN, Tanezumab; COR, Corticosteroids; DIA, Diazepam; SMR, Skeletal muscle relaxants; ANC, Anticholinergics (Diphenhydramine or Benztropine); ALG, Acetylsalicylic acid + Acetaminophen + Caffeine + Chlorpheniramine; BUP, Buprenorphine. Details of the electronic search and study selection flow diagram.

A total of 11,239 studies were identified, and 88 eligible trials with 21,377 participants were finally included in the network meta-analysis. As presented in Supplementary Table S1, 81 eligible trials (22, 43, and 16 studies of acute, chronic, and radicular LBP, respectively) reported data on pain intensity and 47 eligible trials (12, 27, and eight studies of acute, chronic, and radicular LBP, respectively) reported data on function. The numbers of participants with acute, chronic, and radicular LBP were 7,229, 11,912, and 2,236, respectively. Details of the electronic search and selection flow diagram are shown in Figure 1.

**FIGURE 2:**
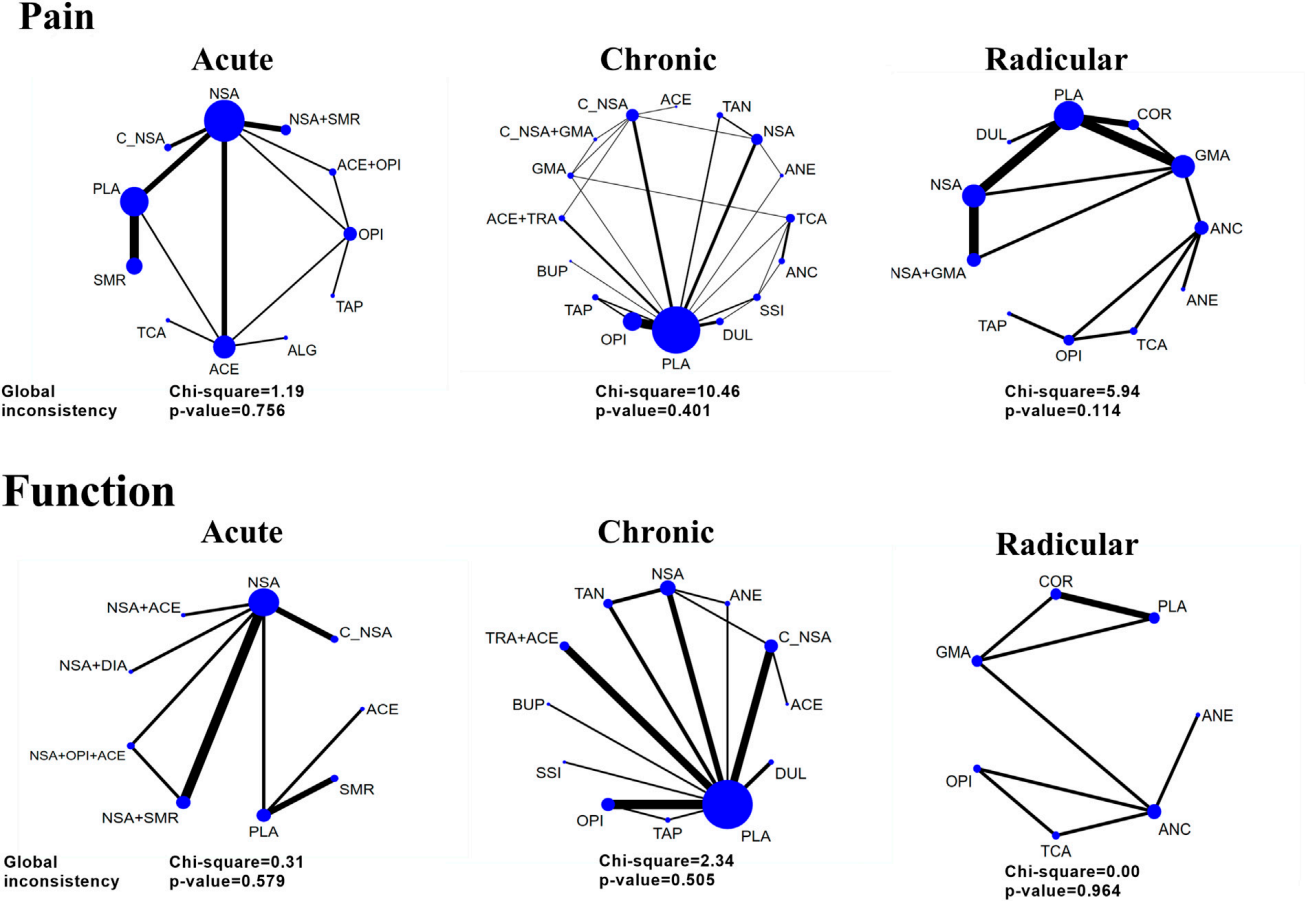
Network and global inconsistency of eligible comparisons for pain intensity and function. Acute, radicular, and chronic low back pain (LBP). The line widths of arcs represent the strength of the relationship between treatments. The wider arc means more direct comparisons between these two treatments.

Figure 2 shows the network plot of eligible comparisons of potency. We assessed the effects of the following single pharmacological treatments for pain relief and functional evaluations: NSAIDs, opioids, corticosteroids, skeletal muscular relaxants, cyclo-oxygenase 2 (COX2)-selective NSAIDs, γ-aminobutyric acid (GABA) mimetic antiepileptics (gabapentin or pregabalin), other antiepileptics, tramadol, tricyclic antidepressants (TCA), selective serotonin reuptake inhibitors (SSRIs), duloxetine, buprenorphine, tanezumab, acetaminophen, anticholinergics (diphenhydramine or benztropine), diazepam and tapentadol. Further, the effects of some co-treatments were assessed.

### Pair-Wise Meta-analysis

The results of the pairwise meta-analysis revealed that skeletal muscle relaxants and NSAIDs were effective for acute LBP, both for pain and function. For radicular LBP, only duloxetine was better than the placebo. For chronic LBP, COX2-selective NSAIDs, opioids, duloxetine, tanezumab and a drug combination (tramadol plus acetaminophen) showed superior efficacy to the placebo. The detailed results of the pair-wise meta-analysis are shown in Supplementary Tables S2–7.

### Network Results of Acute LBP

**FIGURE 3:**
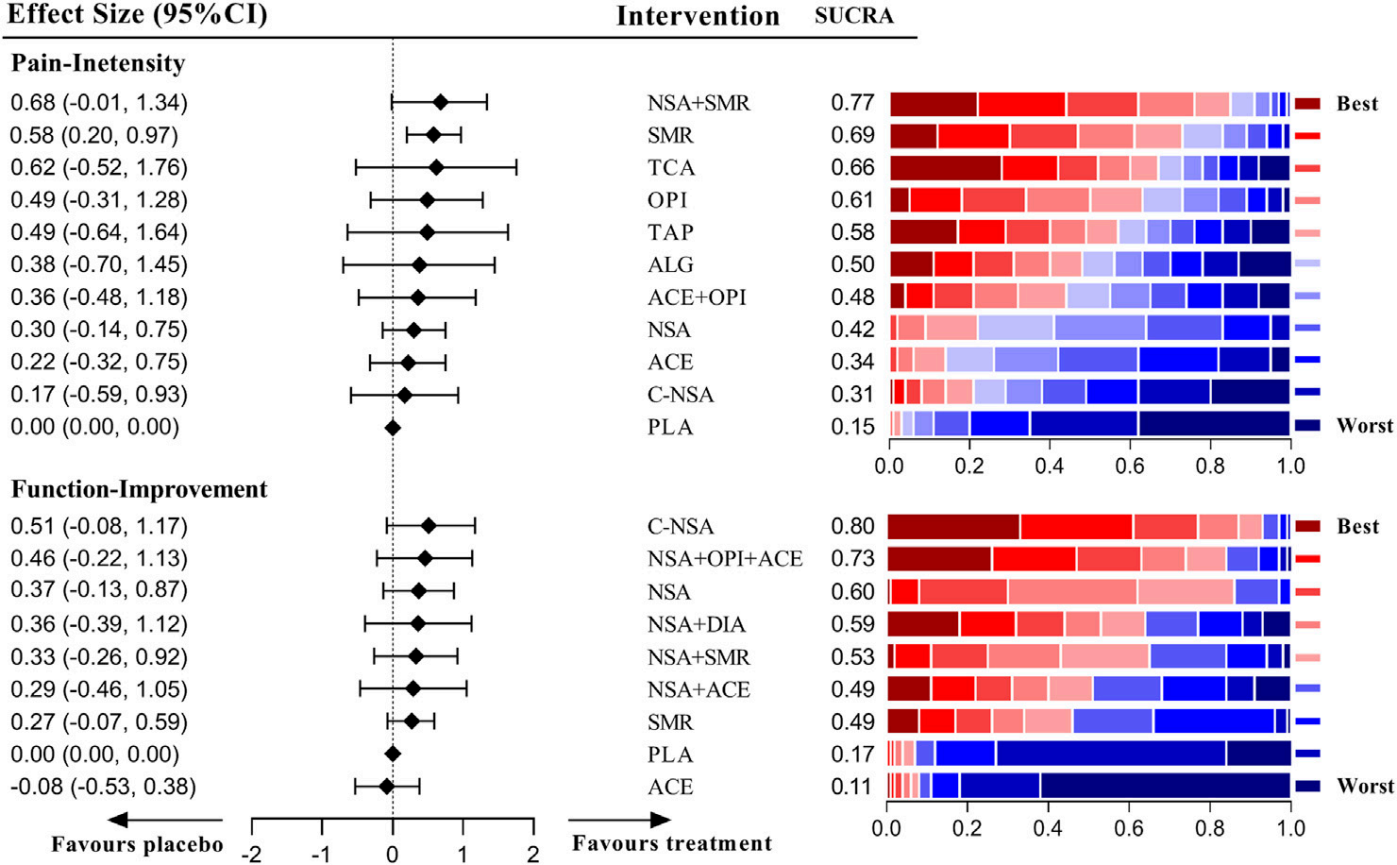
Estimate effects and hierarchy of pharmacotherapies for acute low back pain (LBP).

As shown in Figure 3, the results of the network meta-analysis demonstrated that only the skeletal muscle relaxant was efficient and the most precise (SMD = 0.58 [95% CI, 0.20 to 0.97]). Although NSAIDs plus skeletal muscle relaxants had the highest ranking (SUCRA = 0.77), the pooled result of this intervention was imprecise (SMD = 0.68 [95% CI, −0.01 to 1.34]), and the comparison between NSAIDs plus skeletal muscle relaxants and a single skeletal muscle relaxant did not show superior potency (SMD = 0.10 [95% CI, −0.69 to 0.68]). However, the pooled effects indicated that none of the medications included in this study were effective for improving the condition of disability. The detailed network results for acute LBP are shown in Supplementary Tables S20, 21.

### Network Results of Radicular LBP

**FIGURE 4:**
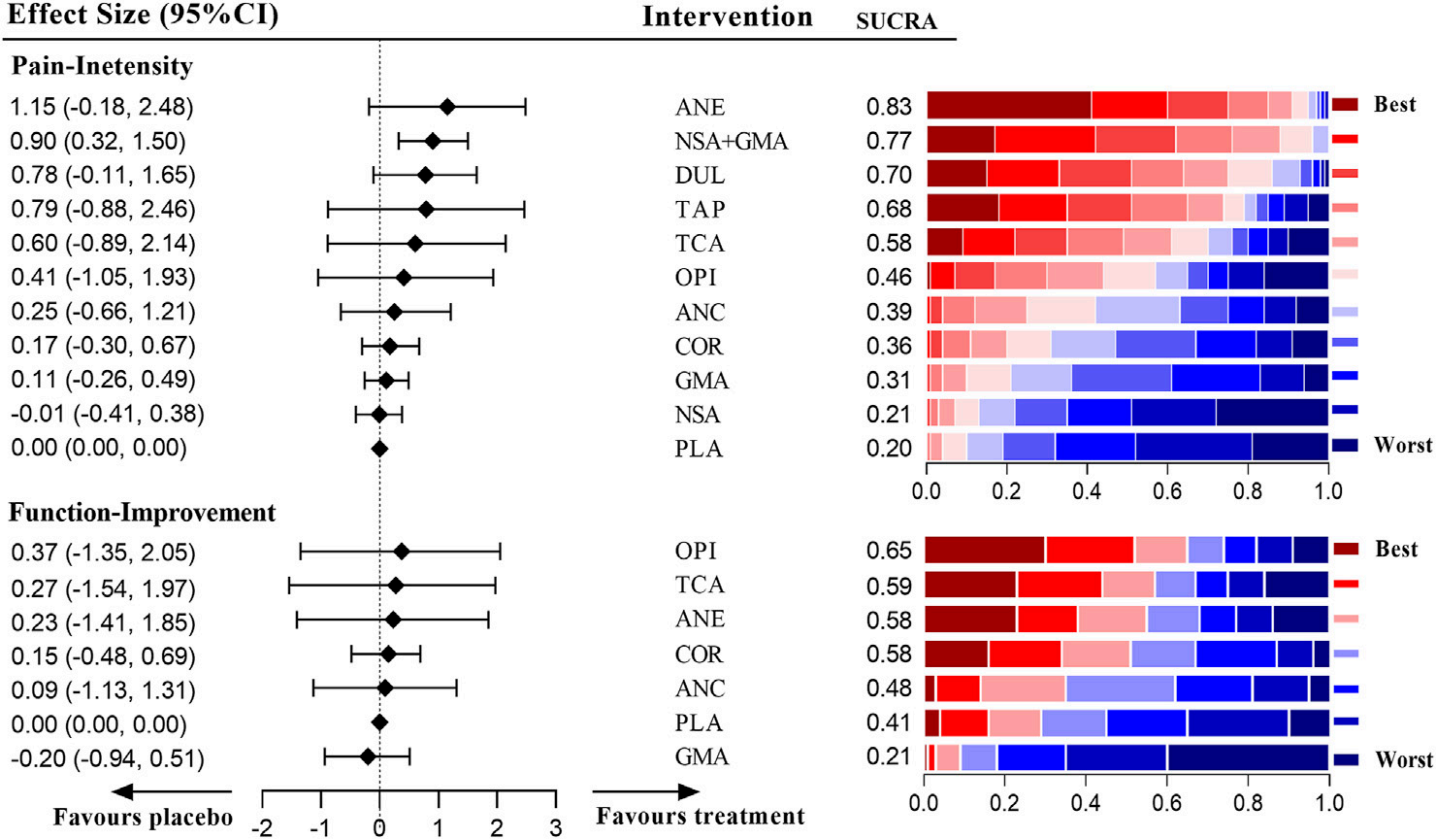
Estimate effects and hierarchy of pharmacotherapies for radicular low back pain (LBP).

Figure 4 shows that only the combination of NSAIDs and GABA mimetic antiepileptics showed better effects than the placebo in pain management of patients with radicular LBP (SMD = 0.90 [95% CI, 0.32 to 1.50]). Moreover, this combination had a better precision than the other drugs that had a similar SUCRA. Antiepileptics (like topiramate) had the highest ranking, but the pooled result of this intervention was imprecise (SMD = 1.15 [95% CI, −0.18 to 2.48]). For the management of function, none of the medications included in this group proved to be superior to the placebo. The detailed network results for radicular LBP are shown in Supplementary Tables S24, 25.

### Network Results of Chronic LBP

**FIGURE 5:**
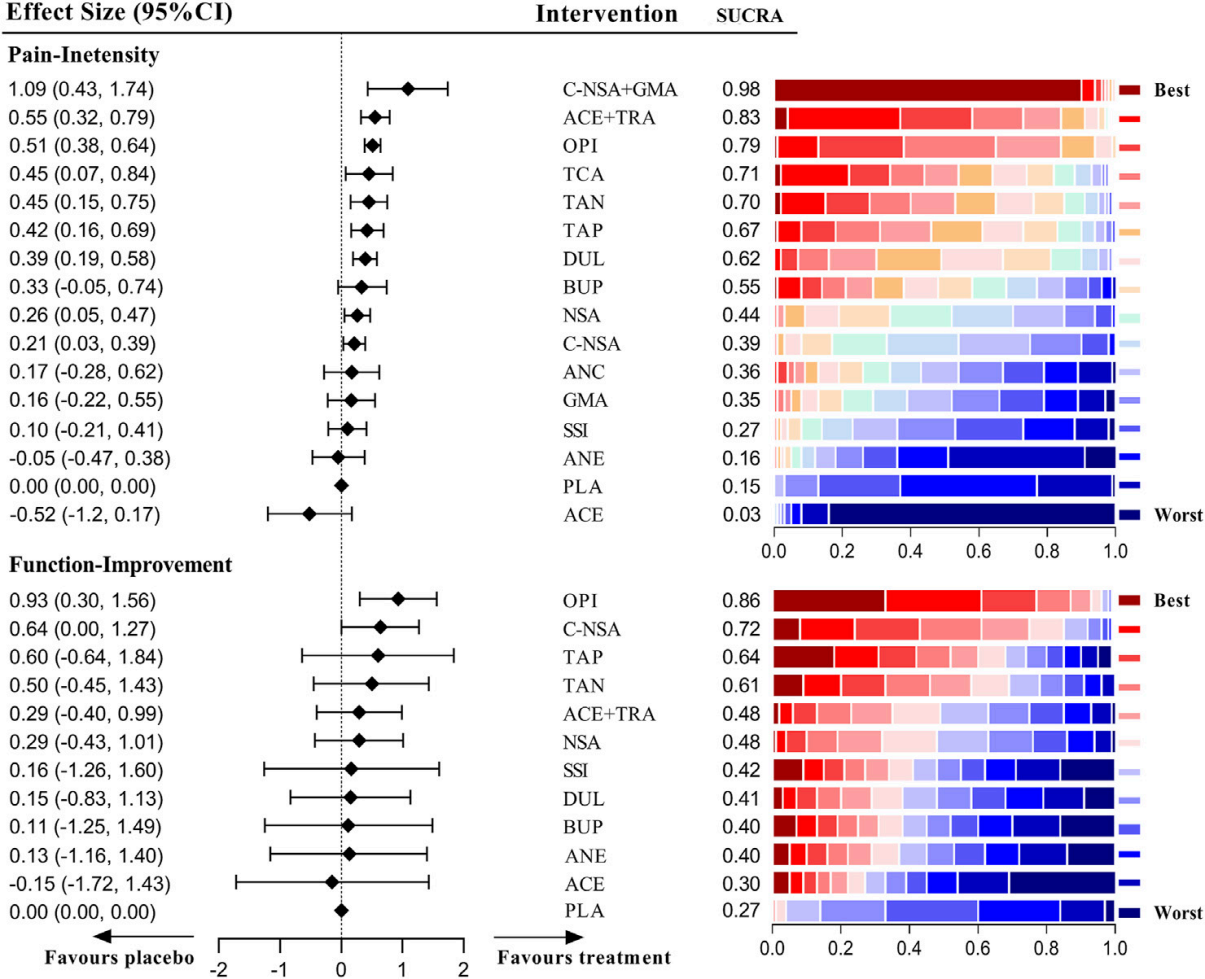
Estimate effects and hierarchy of pharmacotherapies for chronic low back pain (LBP).

Figure 5 shows the effects of different medications on pain and function outcomes compared with the placebo. Most medications evaluated for pain management of LBP were effective, but acetaminophen, GABA mimetic antiepileptics (pregabalin or gabapentin), anticholinergics, SSRIs and buprenorphine were ineffective. Notably, the recommendation ranking of acetaminophen was significantly lower than that of the placebo (SUCRA = 0.03). In the network analysis, the combination of COX2-selective NSAIDs and GABA mimetic antiepileptics seemed to have the best statistical efficiency in pain management of patients with LBP (SUCRA = 0.98); but its precision was lower than most of the treatments. The drug with the best precision was strong opioids, of which the recommendation ranking was three. For the management of function, opioids (SMD = 0.93 [95% CI, 0.30 to 1.56]) and COX2-selective NSAIDs (SMD = 0.64 [95% CI, 0.00 to 1.27]) were proven superior to the placebo. The detailed network results for chronic LBP are shown in Supplementary Tables S22, 23.

### Inconsistency, Risk of Bias, Sensitivity Analyses, and Publication Bias

As illustrated in Figure 2, the global inconsistency assessment did not show any statistically significant difference, and local inconsistency evaluations further confirmed its consistency (*p* > 0.05, Supplementary Tables S8–13). The loop of acute function was formed only by one multi-arm trial, which also did not affect its consistency according to the standard definition (Higgins et al., 2012). The node-splitting inconsistency, which reported the estimated direct and indirect treatment effects and their differences, is illustrated in Supplementary Tables S14–19. However, the funnel plot (Supplementary Figures S1–6) of each outcome did not show any obvious asymmetry, although we found a few direct comparisons which were probably publication biased. In terms of the included studies, direct comparisons and mixed comparisons, their outcomes of risk of bias are respectively illustrated in Supplementary Material S7–10. The contribution matrixes, which reported the contribution of each direct comparison to the mixed comparison, indirect comparison, and the whole network, are illustrated in Supplementary Figures S9–14. Sensitivity analyses revealed that the obtained results were stable (Supplementary Tables S20–25).

### The Quality of the Estimates of Treatment Effect

**FIGURE 6:**
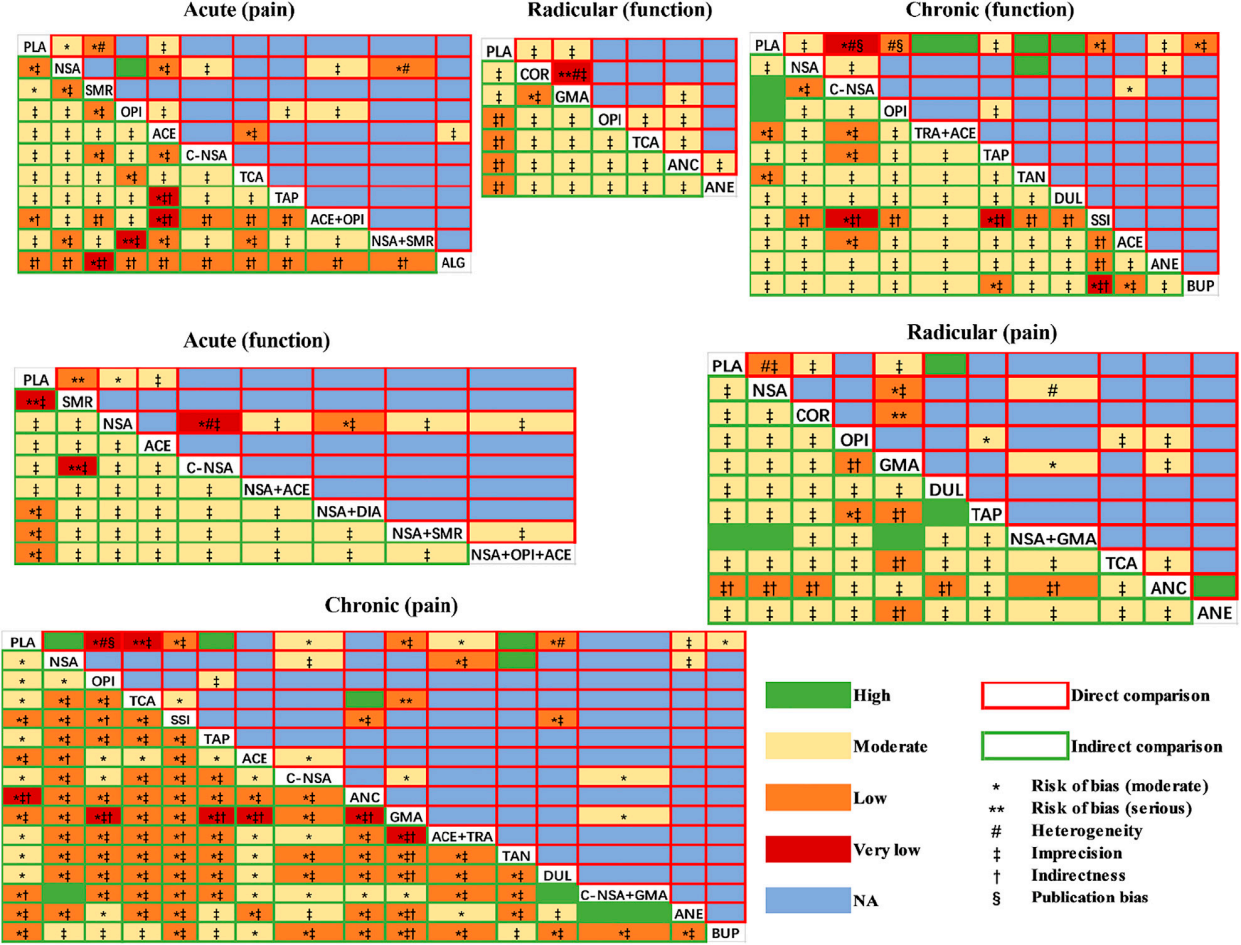
The quality of the estimates of direct and indirect evidence (GRADE approach).

We rated the quality of direct, indirect, and NMA (combined direct + indirect) evidence following the GRADE approach (Puhan et al., 2014; Brignardello-Petersen et al., 2018) in our analyses (Figure 6). There was no evidence of high quality when we analysed the functional improvement of patients with acute and radicular LBP, and most evidence was of moderate quality. However, for the pain relief of patients suffering from chronic LBP, the drug combinations (COX2-selective NSAIDs + pregabalin or gabapentin) were significantly superior to NSAIDs, and NSAIDs showed better effects than placebo. These two comparisons both showed high quality. Another combination (NSAIDs + GABA mimetic antiepileptic) also showed superiority to the placebo with high quality.

## Discussion

This network meta-analysis is the first comprehensive study to synthesize all of the available evidence and to precisely and quantitatively compare the efficacy of numerous pharmacological treatments for patients with LBP. Skeletal muscle relaxants are more effective than the placebo in relieving pain caused by acute LBP, consistent with previous studies. (Chou et al., 2017a). In addition to skeletal muscle relaxants, the previous meta-analyses (Roelofs et al., 2008; Chou et al., 2017a) and some guidelines (ACP and NICE) (de Campos, 2017; Qaseem et al., 2017) have recommended NSAIDs as an effective medication for the pain intensity of acute LBP. However, our results revealed that NSAIDs (including COX2-selective) were not superior to the placebo.

The reasons for this paradoxical finding may be as follows. First, we compared NSAIDs with other interventions, not just the placebo. In the network comparison, the included data revealed that NSAIDs were not more effective (in the emergency department) than acetaminophen, which seemed ineffective in our results and those of other previous high-quality trials. (Eken et al., 2014; Williams et al., 2014). Second, in this network meta-analysis, we categorized patients with sciatica into the radicular LBP group (previous studies did not). (Chou et al., 2017a). Similar to our results, a series of trials with another outcome (pain relief) showed that NSAIDs were noneffective. (Goldie, 1968; Weber and Aasand, 1980; Basmajian, 1989). Considering the controversial effects of NSAIDs and the potential risk of side effects (gastrointestinal, liver, and cardio-renal toxicities), we do not recommend NSAIDs as applicable medications to treat acute LBP with urgent pain.

In the analysis of the pain intensity of radicular LBP, previous systematic reviews found that none of the single medications was effective for radicular LBP. (Chou et al., 2017a). However, our results suggest that combination pharmacotherapy with NSAIDs plus GABA mimetic antiepileptics (pregabalin or gabapentin) exhibited remarkable effects that were considerably more effective than single NSAIDs (recommended to treat nonspecific-LBP by ACP, NICE, and Canadian guidelines) (Qaseem et al., 2017; de Campos, 2017; Group. TOPTLBPW, 2015) or GABA mimetic antiepileptics (gabapentin or pregabalin, recommended to treat neuropathic-pain by NICE guidelines). (de Campos, 2017). This combination pharmacotherapy could be used to reduce prostaglandin-mediated pain and neuropathic pain simultaneously.

However, we have to acknowledge that the limitations of this finding might affect its validity. One of the trials included in this comparison was performed using a crossover-design, which would result in a carry-over effect. (Woods et al., 1989). We have to consider that pregabalin has been reported to be ineffective for sciatica and is associated with significant harms. (Mathieson et al., 2017). Whether the combination of NSAIDs and pregabalin (or gabapentin) is safe and effective for radicular LBP needs more research for confirmation. The current evidence shows that in addition to the fact that exercise has little effect, other non-invasive treatments (including pharmacotherapies and physiotherapies) are noneffective. (Chou et al., 2017b; Qaseem et al., 2017).

For the pain intensity of chronic LBP, previous meta-analyses found that strong opioid agents (morphine, oxymorphone, and others) were effective. (Chaparro et al., 2013). Although we reached the same conclusion from our NMA, we do not recommend opioids as applicable medications to treat chronic LBP in consideration of their overdose and addiction risks. In addition, a previous study found that opioids were associated with a high risk of nausea, dizziness, constipation, and vomiting. (Chaparro et al., 2013; Els et al., 2017). Of note, all of the major guidelines (ACP, NICE, and Canadian guidelines) (Qaseem et al., 2017; de Campos, 2017; Group. TOPTLBPW, 2015) recommend the consideration of central analgesics only when NSAIDs are contraindicated. Moreover, the recommendation ranking opioids was just third.

Some guidelines recommend the use of tramadol and tramadol-acetaminophen in managing the pain intensity of chronic LBP. (de Campos, 2017; Qaseem et al., 2017). We also found that tramadol-acetaminophen combination drugs were significantly effective. However, tramadol is also addictive, although this risk is less than for strong opioid agents. (Bravo et al., 2017). Compared with strong opioids and tramadol-acetaminophen, COX2-selective NSAIDs plus pregabalin (or gabapentin) seemed to be a better choice. However, the comparison of this combination with placebo was of low quality because of the risk of bias and its indirectness. Similar to the findings for radicular LBP, caution should be applied when using pregabalin for chronic LBP.

The ineffectiveness of SSRIs for LBP have long been known, but duloxetine is currently commonly used to control chronic or neuropathic pain. (de Campos, 2017). Recently, a growing number of studies have supported the view that duloxetine is an effective medication for reducing the pain intensity of chronic LBP. (Chou et al., 2017a). The results of this network meta-analysis show the superior analgesic effect of duloxetine, but not that of TCA or SSRIs.

Additionally, some new pharmacological therapies for pain intensity might be available in the near future. (Abbasi, 2017; Knezevic et al., 2017). According to the current eligible data, we found that nerve growth factors (such as tanezumab) were superior to the placebo. However, the safety and tolerability of these drugs are still under evaluation. Considering its safety, NSAIDs (including COX2-seletive NSAIDs) may be applicable and safe medications for chronic LBP, although the SUCRA ranking of these medicines was not high.

Unfortunately, because data on functional improvement were lacking in many of the eligible trials, we were unable to assess the effects of all of the prescribed medications on functional improvement, as detailed as the pain intensity. For function in acute or radicular LBP, none of the pharmacotherapies showed better effects than the placebo. However, a previous study revealed that functional improvement was associated with reduced pain intensity (because the perceived intensity of pain would increase distress and fear). (Lee et al., 2015). We suggest that more high-quality randomized controlled trials should be designed to evaluate this synergistic effect. For the functional improvement of patients with chronic LBP, a previous meta-analysis found that NSAIDs had none to slight effects based on the RMDQ score. (Enthoven et al., 2016). Notably, we found that only COX2-selective NSAIDs and strong opioids were more effective than the placebo for functional improvement. The opioids had a higher SUCRA ranking and higher quality of evidence than COX2-selective NSAIDs. We still hope that COX2-selective NSAIDs could be preferentially used for functional improvement of patients with chronic LBP, following the ACP, NICE, and Canadian guidelines. (Qaseem et al., 2017; de Campos, 2017; Group. TOPTLBPW, 2015).

Our study has some limitations. First, although we used a comprehensive search strategy and strict criteria, a few old trials that were only reported as abstracts were not included. Second, we classified LBP into three groups: acute, chronic, and radicular, but we did not perform a more detailed analysis of each group according to treatment duration and dosage. Further, we believe that the absence of the above analyses limits the clinical applicability of our studies to some extent. Finally, our findings were based on unadjusted estimations, and the various characteristics of the participants (age, sex, ethnicity, and geographical location) might have influenced the synthesized effect size. Furthermore, we found a few direct comparisons which were probably publication biased. We must admit that our analysis is fully based on unadjusted estimations, and this is an assignable limitation. However, we could not find appropriate methods for covariates adjustment in our analysis. Matching‐adjusted indirect comparison (MAIC) and simulated treatment comparison (STC) are not generalizable to larger treatment networks (David, 2020). Meta-regresstion has been derived for only the simple case of binary outcomes and binary covariates so far (David, 2020).

In conclusion, this network meta-analysis is the first to provide comprehensive and quantitative evidence of pharmacological therapies for LBP. For acute LBP, skeletal muscle relaxants decreased the pain intensity with moderate quality of the estimate of effect. For radicular and chronic LBP, a combination of NSAIDs (including COX2-selective NSAIDs) and pregabalin (or gabapentin) seemed to be the best non-invasive treatment to relieve pain. In fact, many previous trials reported that pregabalin or gabapentin were not effective or safe for treating LBP as a single drug (Mathieson et al., 2017; Cairns et al., 2019). Pregabalin or gabapentin were also reported to be addictive and at the risk of misuse (Atkinson et al., 2016; Smith et al., 2016), although these drugs are effective for neuropathic pain (Finnerup et al., 2015). Research about combinations of NSAIDs (including COX2-selective NSAIDs) and pregabalin (or gabapentin) are still rare. We are looking forward to more high-quality trials and studies to be performed, either with this combination or other new pharmacological therapies for LBP.

## Methods

### Search Strategy and Selection Criteria

We searched PubMed, embase, Web of Science, Cochrane Central Register of Controlled Trials (CENTRAL) and Clinical Trial databases for relevant randomized clinical trials (RCTs) published before 30 December, 2019. The details of the search strategy are shown in Figure 1 and Supplementary Material S1.

Studies were selected according to the following criteria: 1) participants were >18 years old and diagnosed with LBP (radicular or non-radicular); 2) the causes of the LBP were not cancer, infection, high-velocity trauma, fractures, pregnancy, or severe neurologic deficits; 3) medications were compared with placebo or another medication; and 4) the reported drug administration route was oral or intravenous. Moreover, we did not apply any language restrictions. The exclusion criteria were trials only published as abstracts or LBP complicated with neck pain.

### Data Extraction

Two investigators independently reviewed all eligible studies and then extracted the relevant data using a predefined data extraction sheet. We extracted authors, trial design and size, detailed characteristics of participants (age, sex, geographical location, duration of pain symptoms, and duration of follow up), intervention, and outcome data. The time point we extracted data was the last one in trials that had multiple time points. The number of patients in each trial was extracted as the number of subjects who completed the trial. If the data were not reported, we extracted the number of initial subjects. We only extracted data reported in the articles, and if the data were graphically presented, we collected them from the related graphical information. (Enthoven et al., 2016). Any disagreements were resolved by team discussions, and the final decision was reached based on a majority vote.

### Outcomes and Study Design

The primary outcome of this study was the mean pain intensity. If more than one pain condition was provided in a single trial, we extracted the data in the following order of hierarchy: average pain intensity > pain on rest > pain on walking > pain on sleep. In addition, our secondary outcome was functional improvement. Quantitative evaluations of function using the Roland Morris disability questionnaire (RMDQ) or the Oswestry disability index (ODI) were included in the analysis.

Then, we classified the symptoms of LBP into three different categories: 1) acute or subacute LBP (pain duration ≤12 weeks), 2) chronic LBP (pain duration >12 weeks), or 3) radicular LBP (pain with neuropathic symptoms). (Chou et al., 2017a).

### Quality Assessment of the Evidence

The Grading of Recommendations Assessment, Development and Evaluation (GRADE) Working Group has developed a sensible and transparent approach to grading the quality of evidence. (Guyatt et al., 2011a; Balshem et al., 2011; Guyatt et al., 2011b; Guyatt et al., 2011c; Guyatt et al., 2011d; Guyatt et al., 2011e; Guyatt et al., 2011f). In the GRADE approach, the evidence is evaluated based on five domains: study limitations (risk of bias), inconsistency and heterogeneity, indirectness, imprecision, and publication bias. The risk of bias was assessed by using the Cochrane risk of bias tool (Higgins, 2011) for each study. Contributions of the included studies to direct and indirect evidence were used to assess the risk of bias of the NMA. Indirectness was identified as surrogate outcomes, study populations or interventions that differed from those of interest (Guyatt et al., 2011e) or intransitivity (Jansen et al., 2011). Imprecision was confirmed if the 95% confidence intervals were wide. When we rated the quality of the evidence, we followed four steps to assess the quality of treatment effect estimates from the NMA: 1. present direct and indirect treatment estimates for each comparison of the evidence network; 2. rate the quality of each direct and indirect effect estimate; 3. present the NMA estimate for each comparison of the evidence network; 4. rate the quality of each NMA effect estimate. For a particular comparison, if both direct and indirect evidence were available, we chose the higher of the two quality ratings as the quality rating for the NMA estimate. (Puhan et al., 2014).

### Statistical Methods

We performed two types of meta-analyses in this study. First, we performed a standard pair-wise meta-analysis using a random-effects model. The heterogeneity and inconsistency in these analyses were assessed with I^2^, τ^2^ and *p*-value. (Higgins, 2011). Further, we conducted a network meta-analysis using a random Bayesian model. Three Markov chains were used in this Bayesian model, and we recorded each trace plot to ensure that the pooled results were convergent. Details of the random Bayesian model are shown in Supplementary Material S2. All of the statistical analyses were performed using WINBUGS, R, and STATA.

The changes in pain intensity and function were considered as continuous outcomes, and the pooled effect size was calculated as standardized mean difference (SMD). Each mean pooled effect size was reported as the corresponding 95% confidence intervals (CI). (Salanti et al., 2008). Further, the possible rank of each intervention was evaluated using surface under the cumulative ranking (SUCRA) probabilities, and higher values indicated a more efficient intervention. (Salanti et al., 2011).

To ensure the transitivity assumption held, we assessed potential inconsistencies between direct and indirect evidence using their specific methods. The global inconsistency was assessed using a design-by-treatment interaction model, which used the χ (Global Burden of Disease Study, 2015) test to confirm the plausibility of assumptive consistency throughout the network analysis. (Higgins et al., 2012). Further, we assessed the local inconsistency by calculating the difference between direct and indirect estimates of the closed loops in every network using the loop-specific approach. We also constructed a node-splitting model, which separates the direct and indirect evidence to evaluate the inconsistency. (Dias et al., 2010).

Additionally, to assess the possible publication bias throughout the network, comparison-adjusted funnel plots were constructed. (Salanti et al., 2011). Moreover, we performed sensitivity analyses for pain intensity by omitting the low-quality trials to verify that our pooled results were stable.

## Data Availability

The original contributions presented in the study are included in the article/Supplementary Material further inquiries can be directed to the corresponding authors.
