# Supplementary material for: Comparative Efficacy of Pharmacological Therapies for Low Back Pain: A Bayesian Network Analysis
Source: Front Pharmacol. 2022 Feb 15;13:811962. doi: 10.3389/fphar.2022.811962 (PMC8892951; doi:10.3389/fphar.2022.811962)
Supplement: Supplementary file 1 [file Table1.docx]

**TABLEI Characteristic of the Included Studies**

| **Study** | **Intervention/(dosage)** | **n(initial)** | **n(complete)** | **Follow-Up/weeks** | **Mean-Age (SD or range)** | **Male (%)** | **Kind** | **Pain Scale** | **Function Scale** | **Geographical Location** |
| --- | --- | --- | --- | --- | --- | --- | --- | --- | --- | --- |
| Amlie E (1987)^1^ | Piroxicam (40-20mg/day)  Placebo | 140  142 | 134  132 | 3 days  7 days | 39.7  36.4 | 58.5%  59.1% | Acute | VAS | NA | NA |
| Atkinson JH (1999)^2^ | Maprotiline(150mg/day)  Paroxetine(30mg/day)  Diphenhydramine(12.5-37.5mg/day) | 33  34  36 | 20  22  32 | 8 | 49.2(9.4) | 63.10% | Chronic | DDS | NA | La Jolla, USA |
| Atkinson JH (2007)^3^ | Fluoxetine (100, 200, 400 ng/ml)  Desipramine (< 60 ng/Ml)  Desipramine (> 60 ng/Ml)  Benztropine mesylate(0.5mg/day) | 43  34  18  26 | 31  19  11  22 | 12 | 46.4(10.2) | 61.20% | Chronic | DDS | NA | San Diego, CA, USA |
| Atkinson JH (2016)^4^ | Gabapentin (up to 3600mg/day)  Placebo | 55  53 | 34  38 | 12 | 54.6(11.3)  57.6(8.9) | 75.5%  81.1% | Radicular | DDS | NA | San Diego, La Jolla, CA, USA |
| Baron R (2010)^5^ | Pregabalin (410 mg/day)  Placebo | 110  107 | 108  107 | 5 | 52.5(11.1)  52.6(12.8) | 50%  45.1% | Radicular | DPRS | NA | Kiel, Germany |
| Baron R (2016)^6^ | Oxycodone-Naloxone (10/5-40/20mg /day) Tapentadol (100-250mg/day) | 128  130 | 112  117 | 12 | 58.4(12.2)  58.1(11.4) | 34.4%  40.8% | Radicular | NRS | NA | Grunenthal, GmbH, Aachen, Germany |
| Bedaiwi MK (2016)^7^ | Acetaminophen (1g/day)  Celecoxib (400mg/day) | 25  25 | 23  24 | 4 | 37.2(10.2)  43.4(11.2) | 23, 56%  24, 48% | Chronic | TBP | ODI | Riyadh, Saudi Arabia/Izmir, Turkey/Southampton, UK/Dublin, Ireland/Toronto, Ontario, Canada |
| Berry H (1982)^8^ | Naproxen (1100 mg/day)  Diflunisal (1000 mg/day)  Placebo | 37  37  37 | NA  NA  NA | 2 | 55 | 35.10% | Chronic | VAS | NA | Lancashire/London, UK |
| Berry H (1988)^9^ | Tizanidine (12mg/day)  Placebo | 59  53 | 51  45 | 3 days  7 days | 44(13)  38(13) | 50.8%  50.9% | Acute | VAS | NA | London, UK |
| Berry H (1988)^10^ | Tizanidine (12mg/day)-Ibuprofen (1.2g/day)  Ibuprofen (1.2g/day) | 51  54 | 46  52 | 3 days  7 days | 43(12.4)  42(12.4) | 52.9%  57.4% | Acute | VAS | NA | Denmark Hill, London |
| Biondi D (2013)^11^ | Tapentadol (50-100mg/4-6hours)  Oxycodone HCl(5-15mg/4-6hours) | 321  324 | 302  311 | 5 days | 45.1(13.9)  44.9(14.4) | 53.7%  46.3% | Acute | NRS | NA | New Jersey, USA |
| Birbara CA (2003)^12^ | Etoricoxib (60 mg/day)  Etoricoxib (90 mg/day)  Placebo | 103  107  109 | 75  72  65 | 4  12 | 52.3(13.3)  52.2(12.4)  51.0(13.7) | 36.6%  36.8%  44.9% | Chronic | VAS | RMDQ | Worcester, MA, USA |
| Buynak R (2010)^13^ | Tapentado ER (200-500mg/day)  Oxycodone CR (40-100mg/day)  Placebo | 321  334  326 | 166  133  152 | 12 | 49.4(13.2)  50.0(14.2)  50.4(14.1) | 39.0%  44.8 %  42.3% | Chronic | NRS | NA | Indiana, USA |
| Chandanwale AS (2011)^14^ | Eperisone(150mg/day)  Placebo | 120  120 | 112  113 | 2 | 41.84(11.8)  40.91(12.4) | 44.64%  49.56% | Acute | VAS | NA | Maharashtra, India |
| Christoph A (2017)^15^ | Placebo  Cebranopadol (200ug/day)  Cebranopadol (400ug/day)  Cebranopadol (600ug/day)  Tapentadol (200mg/day) | 126  131  128  130  126 | 126  129  127  127  126 | 14 | 56.9(12.5)  58.0(11.5)  57.5(11.6)  56.9(11.7)  58.2(11.4) | 39.7%  34.9%  37.0%  26.0%  38.1% | Chronic | NRS | ODI | Munchen, Germany |
| Chu LF (2012)^16^ | Morphine (78 mg/day)  Placebo | 69  70 | 48  55 | 1 months | 44(14.2)  46(13.5) | 63.8%  48.6% | Chronic | VAS | RMDQ | Stanford, USA |
| Coats TL (2004)^17^ | Valdecoxib (40mg/ day)  Placebo | 148  145 | 134  115 | 4 | 48.6(13.3)  48.7(12.6) | 45%  41% | Chronic | VAS | RMDQ | Texas, USA |
| Dapas F (1985)^18^ | Baclofen(30-80mg/day)  Placebo | 100  100 | 88  99 | 2 | 42.7(17-74)  41.8(18-74) | 52%  44% | Acute | VAS | NA | NA |
| Dickens C (2000)^19^ | Paroxetine(20mg/day)  Placebo | 44  48 | 27  34 | 2  4  8 | 44(9.7)  46(10.6) | 47.7%  43.751% | Chronic | VAS | ODI | Manchester University, UK |
| Dreiser RL (2001)^20^ | Meloxicam(7.5mg/day)  Meloxicam(15mg/day)  Placebo | 171  181  180 | 165  167  168 | 1  2 | 47(14)  47(14)  47(14) | 43%  39%  43% | Radicular | VAS | NA | Boulogne, France |
| Dreiser RL (2003)^21^ | Diclofenac-K (12.5-75mg/day)  Ibuprofen (400 mg/day)  Placebo | 124  122  126 | 122  119  121 | 1 | 40.9(10.9)  40.6(11.6)  41.0(11.3) | 48.4%  52.5%  47.2% | Acute | VAS | RMDQ | Issy les Moulineaux, France |
| Eken C (2014)^22^ | Acetaminophen (1 g in 100 ml)  Morphine (0.1 mg/kg in 100 ml)  Dexketoprofen (50 mg in 100 ml) | 46  45  46 | 46  45  46 | 15 mins  30 mins | 31.5(9.5) | 60.60% | Acute | VAS | NA | Antalya, Turkey |
| Enomoto H (2019)^23^ | Duloxetine (60mg/day)  Placebo | 230  226 | NA  NA | 14 | 60(13.2)  57.8(13.7) | 50.0%  46.0% | Chronic | NA | RMDQ | Japan |
| Friedman BW (2015)^24^ | Naproxen(1g/day) -Placebo  Naproxen-Cyclobenzaprine (1g-5mg/day)  Naproxen-Oxycodone /Acetaminophen (1g-5mg/325mg/day) | 107  108  108 | 107  108  108 | 1  12 | 39(11)  38(11)  39(11) | 50%  58%  44% | Acute | NA | RMDQ | Bronx, New York, USA |
| Friedman BW (2017)^25^ | Naproxen-Diazepam(1g-5mg/day)  Naproxen(1g/day) + Placebo | 57  57 | 50  53 | 1  12 | 34(12)  38(12) | 53%  58% | Acute | NA | RMDQ | Bronx, New York, USA |
| Friedman BW (2018)^26^ | Naproxen(1g/day)- placebo  Naproxen- Orphenadrine (1g-200mg/day)  Naproxen-Methocarbamol (1g -750mg/day) | 79  90  81 | 76  78  80 | 1  12 | 39(12)  40(12)  38(12) | 57.0%  57.5%  48.4% | Acute | NA | RMDQ | Bronx, New York, USA |
| Friedman BW (2019)^27^ | Ibuprofen (1.8g/day)-Placebo  Ibuprofen(1.8g/day)-Baclofen(30-60mg/day)  Ibuprofen(1.8g/day)-Metaxalone(1.2-2.4g/day)  Ibuprofen (1.8g/day)-Tizanidine(6-12mg/day) | 80  80  80  80 | 74  79  76  76 | 2 days  1 | 39(11)  39(12)  37(10)  40(11) | 55.0%  71.0%  55.0%  53.0% | Acute | NA | RMDQ | Bronx, New York, USA |
| Friedman BW (2020)^28^ | Ibuprofen (1.8g/day)-Placebo  Ibuprofen (1.8g/day)-Acetaminophen (0.5-1g/per) | 60  60 | 53  57 | 2 days  1 | 41(13)  41(12) | 53.0%  52.0% | Acute | NA | RMDQ | Bronx, New York, USA |
| Goldberg H (2015)^29^ | Prednisone (cumulative dose of 600 mg)  Placebo | 181  88 | 157  77 | 3  52 | 46.0(12.1)  45.6(11.8) | 55.4%  54.1% | Radicular | NRS | ODI | Oakland/San Francisco/Redwood City, California, USA |
| Goodkin K (1990)^30^ | Trazodone(201mg/day)  Placebo | 22  20 | 22  20 | 2  6 | 51.4(13.1)  56.1(12.6) | 63.6%  60% | Chronic | VAS | NA | Stanford, California, USA |
| Gurrell R (2018)^31^ | Naproxen (1g/day)  PF-06372865(selective GABAA modulator) (5-15mg/day)  Placebo | 74  74  74 | 65  65  58 | 4 | 51.6(21-75)  49.7(18-72)  51.1(22-74) | 50.00%  47.30%  48.65% | Chronic | NRS | RMDQ | Granta Park, Cambridge, UK |
| Hale M (2010)^32^ | Hydromorphone(64mg/day)  Placebo | 134  134 | 66  44 | 1/2/3/8/12 | 47.8(10.5)  49.4(10.6) | 54.1%  45.1% | Chronic | NRS | RMDQ | Weston, USA |
| Hale ME (2007)^33^ | Oxymorphone(10mg/day)  Placebo | 70  73 | 49  18 | 12 | 48.2(11.7)  46.0(11.3) | 42.9%  66.7% | Chronic | VAS | NA | Weston, Florida, USA. |
| Herrmann WA (2009)^34^ | Lornoxicam (8mg/day)  Diclofenac (50mg/day)  Placebo | 57  57  57 | 53  55  56 | 0-8 hours | 51.8(14.5)  48.9(13.8)  48.4(11.8) | 44%  47%  42% | Radicular | VAS | NA | Munich, Germany |
| Innes GD (1998)^35^ | Ketorolac (10 mg/4 to 6 h)  Acetaminophen-Codeine (600mg-60mg/ 4 to 6 h) | 63  60 | 55  58 | 0-6 hours | 33.1(9.9)  36(10.1) | 80.6%  76.7% | Acute | VAS | NA | New Westminster, BC, Canada |
| Jenkins DG (1976)^36^ | Tofranil (75 mg/day)  Placebo | 30  29 | 23  21 | 4 | 52.3(13.3)  51.0(13.7) | 36.6%  44.9% | Chronic | VAS | NA | NA |
| Kalita J (2014)^37^ | Pregabalin (150-300mg/day)  Amitriptyline (12.5-50 mg/day) | 97  103 | 70  77 | 6  14 | 42.0(11.6)  41.6(10.7) | NA | Chronic | VAS | NA | Lucknow, India |
| Katz N (2003)^38^ | Rofecoxib (25 mg/day)  Rofecoxib (50 mg/day)  Placebo | 233  229  228 | 198  197  185 | 4 | 53.4(13.2)  52.5(12.9)  54.2(12.7) | 37.3%  38.4%  37.3% | Chronic | VAS | NA | Boston, USA |
| Katz N (2007)^39^ | Oxymorphone (5–10 mg/12 h/3–7 days)  Placebo | 105  100 | 71  47 | 12 | 51.3(13.9)  48.1(12.4) | 43.8%  50.0% | Chronic | VAS | NA | Boston, MA, USA |
| Katz N (2011)^40^ | Tanezumab(200μg/kg)  Naproxen (1g/day)  Placebo | 88  88  41 | 59  64  25 | 6  12 | 49.5(14.7)  52.1(14.8)  52.2(15.0) | 39.8%  52.3%  43.9% | Chronic | NRS | RMDQ | Needham, MA, USA. |
| Katz N (2015)^41^ | Xtampza ER(40-160mg/day)  Placebo | 193  196 | 122  100 | 12 | 49.2(13.3) | 46.60% | Chronic | NRS | RMDQ | Boston, MA/ Hendersonville, NC, USA |
| Kawamata M (2019)^42^ | Oxycontin (5-80mg/day)  Placebo | 62  68 | 46  37 | 5 | 62.8 (13.2)  64.9 (12.0) | 53.2%  47.1% | Chronic | BPI | RMDQ | Matsumoto, Japan |
| Ketenci A (2005)^43^ | Tizanidine (6 mg/day)  Thiocolchicoside (16mg/day)  Placebo | 32  38  27 | 31  35  26 | 3 days  1 | 37  37  40 | 37.5%  42.1%  48.1% | Acute | VAS | NA | Istanbul, Turkey |
| Khoromi S (2005)^44^ | Topiramate (50-400mg/day)  Diphenhydramine(6.25-50mg/day) | 21  20 | 13  16 | 2 | 53(28-74)  60.5(32-74) | 55%  50% | Radicular | VAS | ODI | Bethesda, MD, USA |
| Khoromi S (2007)^45^ | Morphine (15-90mg/day)  Nortriptyline (25-100mg/day)  Morphine-Nortriptyline (15-90-25-100mg/day)  Benztropine (0.25-1 mg/day) | 13  15  13  14 | 9  7  5  7 | 4 | 52.5(30-64) | 50% | Radicular | VAS | ODI | Bethesda, MD, USA. |
| Kivitz AJ (2013)^46^ | Placebo  Tanezumab(5mg/day)  Tanezumab(10mg/day)  Tanezumab(20mg/day)  Naproxen(500mg/day) | 230  232  295  295  295 | 128  146  193  188  185 | 16 | 51.2 (18–85)  51.5 (19–89)  52.0 (18–84)  51.2 (20–81)  52.6 (18–87) | 45.7%  50.4%  46.8%  44.1%  48.5% | Chronic | LBPI | RMDQ | Duncansville, USA |
| Ko S (2016)^47^ | Glucocorticoid triamcinolone (4 mg/day)  Pregabalin (7.5mgpregabalin/100mg Gabapentin) | 27  27 | 20  20 | 6  12 | 62.5(12.7)  62.6(13.2) | 30%  35% | Radicular | VAS | RMDQ | Daegu, Korea |
| Kopecky EA (2017)^48^ | Xtampza™ ER oxycodone (40-160mg/day)  Placebo | 190  194 | NA  NA | 12 | 68.8(31.4) | 43.20% | Chronic | NRS | NA | Canton, USA |
| Lee JH (2013)^49^ | TA-ER (tramadol hydrochloride 75-mg/acetaminophen 650-mg fixed-dose combination tablets)  Placebo | 123  120 | 92  104 | 4 | 59.9(10.7)  60.4(9.9) | 24.8%  25.8% | Chronic | VAS | ODI(Korea) | Seoul, Korea |
| Markman J (2018)^50^ | Oxycodone DETERx (20-160mg/day)  Placebo | 22  22 | 14  4 | 12 | 49.4 (14.4)  50.8 (11.2) | 40.9%  45.5% | Chronic | NRS | NA | Rochester, NY, USA |
| Markman J (2019)^51^ | NKTR-181 (200mg/day)  Placebo | 309  301 | 249  242 | 12 | 52(12.7)  50.7(12.5) | 39.5%  43.5% | Chronic | NRS | RMDQ | Rochester, NY, USA |
| Markman JD (2015)^52^ | Pregabalin (75mg/day)  Diphenhydramine(12.5mg/day) | 29  29 | 24  28 | 1 | 53(19-65)  52.5(30-64) | 54%  50% | Radicular | NRS | ODI | Rochester, NY, USA/Cambridge, MA, UK |
| Mathieson S (2017)^53^ | Pregabalin(150-600mg/day)  Placebo | 106  101 | 83  80 | 2/4/8/12/26/52 | 52.4(17.2)  55.2(16.0) | 62.3%  48.5% | Radicular | VAS | RMDQ | NA |
| Mazza M (2010)^54^ | Escitalopram(20mg/day)  Duloxetine(60mg/day) | 41  44 | 39  41 | 13 | 52.0(12.4)  53.6(13.5) | 43.6%  43.1% | Chronic | LS | NA | Rome, Italy |
| Mccleane GJJPC (2000)^55^ | Gabapentin (300 mg/day)  Placebo | 30  30 | 24  24 | 1  6 | 52.1(14.8)  52.1(14.8) | 46.05%  46.05% | Chronic | NRS | NA | NA |
| Miki K (2018)^56^ | Acetaminophen (2.4g/day)  Loxoprofen (60mg/day) | 64  63 | 35  35 | 2  4 | 66.73 (2.29)  63.50 (19.4) | 32.81%  34.92% | Acute | NRS | NA | Suita, Japan |
| Nadler SF (2002)^57^ | Ibuprofen(200mg/day)  Acetaminophen(500mg/day)  Placebo  Heat Wrap  Unheated Wrap | 106  113  20  113  20 | 102  111  19  111  20 | 1 | 34.9(11.29)  36.61(10.40)  38.00(9.07)  35.82(10.54)  36.79(9.32) | 43.4%  40.6%  40.0%  41.6%  42.1% | Acute | VRS-6 | RMDQ | Newark, USA. |
| Nakashima H (2019)^58^ | Loxonin(180mg/day)  Pregabalin (150mg/day) –Loxonin (180mg/day) | 30  30 | 30  26 | 2  4 | 53.7(15.5)  48.1(12.8) | 56.67%  53.85% | Radicular | VAS | NA | Japan |
| Pallay RM (2004)^59^ | Etoricoxib (60mg/day)  Etoricoxib (90mg/day)  Placebo | 109  106  110 | 77  77  77 | 4  12 | 53.3 (12.3)  53.3 (12.7)  51.8(13.5) | 32.1%  41.5%  39.1% | Chronic | VAS | RMDQ | Hillsboro, NJ, USA |
| Pareek A (2009)^60^ | Aceclofenac–Tizanidine (100mg-2 mg twice)  Aceclofenac (100mg twice) | 101  96 | 94  91 | 3 days  1 | 43.0(12.2)  43.35(11.0) | 61.39%  60.42% | Acute | VAS | NA | Mumbai, India |
| Patel HD (2019)^61^ | Chlorzoxazone(1.5g/day)-Ibuprofen(1.2g/day)  Ibuprofen (1.2g/day) | 203  203 | NA  NA | 3 days  1 | 43.3(11.14)  41.3(11.26) | 40.89%  42.36% | Acute | VAS | NA | India |
| Peloso PM (2004)^62^ | Tramadol-Acetaminophen (37.5-325 mg/day)  Placebo | 167  169 | 86  61 | 14 | 57.5(11.5)  57.5(13.6) | 35.9%  39.1% | Chronic | VAS | RMDQ | Iowa City, USA |
| Perrot S (2006)^63^ | Acetaminophen-Tramadol (325-37.5mg/day)  Tramadol(50mg/day) | 59  60 | 51  48 | 3 days  7 days  10 days | 56.5(15.3)  54.1(14.6) | 35.6%  48.3% | Acute | VAS | NA | Paris, France |
| Pohjolainen T (2000)^64^ | Ibuprofen (1.8g/day)  Nimesulide (200 mg/day) | 52  52 | 52  50 | 3days  1 | 42.0(19-63)  42.5(22-63) | 50% | Acute | VAS | ODI | Espoo, Finland |
| Ralph L (2008)^65^ | Carisoprodol (250 mg/day)  Placebo | 277  285 | 269  278 | 3 days  1 | 39.3(11.8)  41.5(11.7) | 51.3%  45% | Acute | NRS | RMDQ | San Diego, USA |
| Rauck RL (2014)^66^ | Hydrocodone(40-200mg/day)  Placebo | 151  151 | 124  59 | 12 | 50.4(10.9)  50.8(12.4) | 38.4%  51.0% | Chronic | NRS | NA | Winston-Salem, NC, USA |
| Rauck RL (2016)^67^ | Buprenorphine(300-900ug/day)  Placebo | 229  232 | 209  211 | 12 | 51.2(12.6)  49.0(13.1) | 46.3%  41.4% | Chronic | NRS | RMDQ | Malvern, PA /Raleigh, NC, USA. |
| Rodrigues LC (2014)^68^ | Prednisone(1mg/kg/day)  Placebo | 31  30 | 31  30 | 3  6  12 | 58.23(6.38)  58.33(6.19) | 50% | Radicular | VAS | RMDQ | Sao Paulo, Brazil |
| Romanò CL (2009)^69^ | Celecoxib (3–6 mg/kg/die)  Pregabalin (1–4 mg/kg/die)  Celecoxib-Pregabalin (3–6mg/kg/die-1–4 mg/kg/die) | 42  42  42 | 36  36  36 | 4 | 53(16) | 44.40% | Chronic | VAS | NA | Via Riccardo Galeazzi, Milan, Italy |
| Ruoff GE (2003)^70^ | Tramadol-Acetaminophen (37.5-325 mg/day)  Placebo | 161  157 | 91  74 | 12 | 53.6(11.9)  54.1(12.0) | 32.9%  40.8% | Chronic | VAS | RMDQ | Kalamazoo, USA. |
| Schiporst Preuper HR (2014)^71^ | Tramadol-Acetaminophen (37.5-325 mg/day)  Placebo | 25  25 | 24  25 | 2 | 42.0 (35.5–50.5)  44.0 (32.5–48.0) | 28%  36% | Chronic | VAS | RMDQ | Haren, The Netherlands |
| Schukro RP (2016)^72^ | Duloxetine (up to 120mg/day)  Placebo | 41  41 | 31  29 | 4 | 57.9(13.4) | 49% | Radicular | VAS | NA | Vienna, Austria |
| Serfer GT (2010)^73^ | Carisoprodol (250mg/day)  Carisoprodol (350mg/day)  Placebo | 264  273  269 | 259  269  265 | 3 days  1 | 40.9(11.7)  40.5(12.4)  40.7(13.1) | 47.7%  44.3%  39.4% | Acute | NA | RMDQ | Hollywood, FL, USA |
| Skljarevski V (2009)^74^ | Duloxetine(20mg/day)  Duloxetine(60mg/day)  Duloxetine(120mg/day)  Placebo | 59  116  112  117 | 43  80  62  82 | 1-13 | 52.9(12.8)  53.3(14.7)  54.9(14.8)  54.0(13.5) | 39.0%  42.2%  42.0%  45.3% | Chronic | LS | RMDQ | Indianapolis, IN, USA |
| Skljarevski V (2010)^75^ | Duloxetine(60mg/day)  Placebo | 198  203 | 147  156 | 12 | 54.9(13.7)  53.4(14.2) | 40.4%  36.9% | Chronic | BPI | RMDQ | Indianapolis, Indiana, USA |
| Skljarevski V (2010)^76^ | Duloxetine(60-120mg/day)  Placebo | 115  121 | 109  116 | 1-13 | 51.8(14.9)  51.2(13.5) | 38.3%  39.7% | Chronic | BPI | RMDQ | Indianapolis, Indiana, USA |
| Stein D (1996)^77^ | Amitriptyline(150mg/day)  Acetaminophen(2000mg/day) | 20  19 | NA  NA | 1-6 | 36.25(6.8)  36.73(7.9) | 55%  73.7% | Acute | VAS | NA | Bat-Yam, Israel |
| Szpalski M (1994)^78^ | Tenoxicam(20mg)  Placebo | 37  36 | 33  35 | 8 days  15 days | 37.5(9.2)  38.9(10.4) | 62.2%  67.7% | Acute | VAS | NA | Brussels, Belgium |
| Tetsunaga T (2015)^79^ | Tramadol-Acetaminophen (37.5-325mg/day)  Celecoxoib (200mg/day) | 35  35 | 35  35 | 8 | 65.4  62.3 | 37.1%  37.1% | Chronic | NRS | ODI | Kitaku, Okayama, Japan |
| Tsuji T (2017)^80^ | Duloxetine (60 mg/day)  Placebo | 230  226 | NA  NA | 2/4/6/10/14 | 60.0(13.16)  57.8(13.7) | 50%  46.0% | Chronic | BPI | NA | Kitaku, Okayama, Japan |
| Urquhart DM (2018)^81^ | Amitriptyline (25mg/day)  Benztropine mesylate (1mg/day) | 72  74 | 61  57 | 3 months  6 months | 43.5(14.2)  56.0(13.2) | 61%  62% | Chronic | VAS | RMDQ | Australia |
| Voicu VA (2019)^82^ | Paracetamol (1.5g/day)  Algopirin (1 tablet/day) | 45  44 | 43  44 | 1/2/4/6 hours | 47.38(12.51)  46.94(11.42) | 35.6%  47.7% | Acute | VAS | NA | Bucharest, Romania |
| Weber H (1993)^83^ | Piroxicam (40 mg-20 mg/days)  Placebo | 120  94 | NA  NA | 2 | NA | NA | Radicular | VAS | RMDQ | Oslo, Norway |
| Wen W (2015)^84^ | Hydrocodone(20-120mg/day)  Placebo | 296  292 | 229  210 | 2  4  8  12 | 49.2(13.5)  47.9(13.2) | 42%  43% | Chronic | NRS | ODI | Stamford, CT, USA |
| Williams CM (2014)^85^ | Acetaminophen (3990mg/day, regular)  Acetaminophen (as-needed)  Placebo | 550  549  553 | 535  533  538 | 1  2  4  12 | 44.1(14.8)  45.4(16.7) 45.4(15.9) | 52%  53%  55% | Acute | VAS | RMDQ | Darlinghurst, NSW, Australia |
| Ximenes A (2007)^86^ | Valdecoxib (40 mg/day)  Diclofenac (75 mg/day) | 170  170 | 167  166 | 1 | 41.6(11.7)  40.1(12.7) | 49%  49% | Acute | VAS | ODI | Goiania, GO, Brazil. |
| Yaksi A (2007)^87^ | Gabapentin (up to 300-2400mm/day)- NSAIDS  NSAIDS | 28  27 | NA  NA | 17 | 50.8(10.0) | 37% | Radicular | VAS | NA | Istanbul, Turkey |
| Zerbini C (2005)^88^ | Etoricoxib (60mg/day)  Diclofenac(150mg/day) | 224  222 | 204  197 | 4 | 51.7(14.7)  52.2(12.8) | 28.1%  28.4% | Chronic | LBP-IS | RMDQ | Sao Paolo, Brazil |

RMDQ = Roland Morris Disability Questionnaire, ODI = Oswestry Dability Index, VAS = Visual Analogue Scale, NRS = Numerical Rating Scale, DDS = Descriptor Differential Scale, DPRS = Daily Pain Rating Scale Scores, TBP = Total Back Pain, LBPI = Low Back Pain Intensity, LS = Likert Scale, BPI = Brief Pain Inventory, VRS-6 = 6-Point Verbal Rating Scale, LBP-IS = Low Back Pain Intensity Scale

Full references for all trials are showed in the supplementary file 14。
