# Supplementary material for: Comparative Efficacy of Pharmacological Therapies for Low Back Pain: A Bayesian Network Analysis
Source: Front Pharmacol. 2022 Feb 15;13:811962. doi: 10.3389/fphar.2022.811962 (PMC8892951; doi:10.3389/fphar.2022.811962)
Supplement: Supplementary file 2 [file DataSheet1.docx]

**Title: Comparative efficacy of pharmacological therapies for low back pain: a Bayesian network analysis**

**Authors:**

Jiuzhou Jiang, MD ^1†^; Hao Pan ^2†^; Haomai Chen ^1†^; Liyang Song ^2^, MD; Yiyun Wang ^1^; Bao Qian, MD ^1^; Pengfei Chen, MD ^1^; Shunwu Fan, MD ^1^^*^; Xianfeng Lin, MD ^1*^

**Affiliations:**

^1^ Department of Orthopaedic Surgery, Sir Run Run Shaw Hospital, Medical College of Zhejiang University, Hangzhou, China

^2^ Department of Orthopaedics, The First Affiliated Hospital of Wenzhou Medical University, Wenzhou

**Contents**

1. Search Strategy and Results……………………………………….…**2**
2. Bayes Model……………………………………………………….…**4**
3. Results of Pairwise Meta-Analysis……………………………….….**6**
4. Local Inconsistency……………………………………………….….**9**
5. Node-splitting Inconsistency………………………………………...**11**
6. Funnel Plots……………………………………………………….…**14**
7. Risk of Bias, Included Studies……………………………………….**17**
8. Risk of Bias, Direct Comparisons…………………………………....**20**
9. Contribution Matrix………………………………………………….**21**
10. Risk of Bias, Whole Network………………………………………...**26**
11. Result of Network Meta-Analysis and Sensitivity Analysis………….**29**
12. References of the Included Studies…………………………………...**35**

**Abbreviations (for the whole document):**

PLA = Placebo, OPI = Opioids, NSA = Non-steroidal anti-inflammatory drugs, TCA = Tricyclic antidepressant, TAP = Tapentadol, SSI = Selective serotonin reuptake inhibitor, GMA = GABA mimetic antiepileptics (Pregabalin or Gabapentin), ANE = Antiepileptic (apart from Pregabalin and Gabapentin), TRA = Tramadol, DUL = Duloxetine, C-NSA = Cyclo-oxygenase 2-selective non-steroidal anti-inflammatory drugs, ACE = Acetaminophen, TAN = Tanezumab, COR = Corticosteroids, DIA = Diazepam, SMR = Skeletal muscle relaxants, ANC = Anticholinergics (Diphenhydramine or Benztropine), ALG = Acetylsalicylic acid + Acetaminophen + Caffeine + Chlorpheniramine, BUP = Buprenorphine

**1.Search Strategy and Results**

1.1 Search strategy:

#1 (low back pain* OR lower back pain* OR low back ache* OR lumbago OR sciatic neuralgia* OR sciatic pain* OR lumbar pain* OR “spinal stenosis*” OR “lumbar stenosis*” OR lumbar disc*) OR ("Low Back Pain"[Mesh] OR "Sciatic Neuropathy"[Mesh] OR "Lumbosacral Region"[Mesh] OR "Intervertebral disc disease" [Supplementary Concept] OR "Spinal Stenosis"[Mesh])

#2 (Anodyne* OR Antinociceptive agent* OR paregoric* OR obtundent OR acesodyne OR analgesic* OR Opioid* OR morphine OR oxycodone OR dihydrone OR oxycodeinon OR dihydrohydroxycodeinone OR pancodine OR eucodal OR oxymorphone OR hydrocodone OR dicodid OR dolantin OR Buprenorphine OR buantnex OR pethidine OR codeine OR methadone OR dolophine OR physeptone OR fentanyl OR pentazocine OR Tramadol OR Tapentadol) OR ("Analgesics"[Mesh] OR "Morphinans"[Mesh] OR "Tramadol"[Mesh] OR "tapentadol" [Supplementary Concept])

#3 (Nonsteroidal Anti Inflammatory Agent* OR NSAIDs OR Aspirin Like Agent* OR Anti Rheumatic Agent* OR ibuprofen OR ketoprofen OR flurbiprofen OR naproxen OR naprosyn OR indomethacin OR sulindac OR fenbid OR celecoxib OR arcoxia OR valdecoxib OR diclofenac OR rofecoxib OR Tenoxicam OR piroxicam OR meloxicam OR lornoxicam OR ketorolac OR phenylbutazone OR butazodine OR flufenamic acid OR acetaminophen* OR paracetamol OR Hydroxyacetanilide OR APAP OR Acephen OR Tylenol OR Panadol) OR ("Antirheumatic Agents"[Mesh] OR "Phenylpropionates"[Mesh] OR "Naproxen"[Mesh] OR "Indomethacin"[Mesh] OR "Sulindac"[Mesh] OR "Celecoxib"[Mesh] OR "Piroxicam"[Mesh] OR "Acetaminophen"[Mesh])

#4 (Antidepressants* OR reboxetine OR bupropion OR “noradrenergic and specific serotonergic Antidepressants” OR NSASA or mirtazapine OR “serotonin norepinephrine reuptake inhibitor*” OR SNRI OR venlafaxine OR duloxetine OR “selective serotonin reuptake inhibitor” OR SSRI OR fluoxetine OR citalopram OR paroxetine OR sertraline OR fluvoxamine OR escitalopram OR tricyclic OR TCA OR amersergide OR amitriptyline OR amineptine OR amoxapine OR butriptyline OR clomipramine OR chlorpoxiten OR desipramine OR demexiptiline OR dibenzipin OR dothiepin OR doxepin OR imipramine OR lofepramine OR melitracen OR metapramine OR nortriptyline OR quinupramine OR opipramol OR protriptyline OR tianeptine OR trimipramine) OR("Antidepressants Agents"[Mesh] OR "Bupropion"[Mesh] OR "Serotonin and Noradrenaline Reuptake Inhibitors"[Mesh] OR "Duloxetine Hydrochloride"[Mesh] OR "Fluoxetine"[Mesh]) OR "Citalopram"[Mesh]) OR "Paroxetine"[Mesh] OR "Sertraline"[Mesh] OR "Fluvoxamine"[Mesh] OR "Antidepressive Agents, Tricyclic"[Mesh] OR "Amitriptyline"[Mesh] OR "Clomipramine"[Mesh] OR "Desipramine"[Mesh] OR "Dothiepin"[Mesh] OR "Nortriptyline"[Mesh] OR "Opipramol"[Mesh] OR "Protriptyline"[Mesh] OR "Trimipramine"[Mesh])

#5 (“skeletal muscle relaxant*” OR neuromuscular effect* OR neuromuscular agent* OR “neuromuscular Blocking Agent*” OR rocuronium OR esmeron OR norcuron OR vecuronium OR atracurium OR tracrium OR pancuronium OR pavulon OR rapacuronium OR mivacurium OR eperisone OR tizanidine OR zanaflex OR sirdalud OR clofen OR baclofen) OR ("Neuromuscular Agents"[Mesh] OR "Vecuronium Bromide"[Mesh] OR "Atracurium"[Mesh] OR "Pancuronium"[Mesh] OR "Baclofen"[Mesh])

#6 (benzodiazepine* OR chlordiazepoxide OR GABA OR librium OR chlozepid OR elenium OR etizolam OR flutazolam OR meprobamate OR miltown OR tranmep OR clonazepam OR lorazepam OR ativan OR hydroxyzine OR midazolam OR quazepam OR diazepam) OR ("Benzazepines"[Mesh] OR "Chlordiazepoxide"[Mesh] OR "Clonazepam"[Mesh] OR "Lorazepam"[Mesh] OR "Hydroxyzine"[Mesh] OR "Midazolam"[Mesh] OR "Diazepam"[Mesh] OR "Carbamazepine"[Mesh] OR)

#7 (anticonvulsant* OR antiepileptic* OR antiepileptic OR Antiseizure OR gabapentin OR neurontin OR topiramate OR Topamax OR tegretol OR carbamazepine OR dilantin OR Phenytoin OR antisacer OR valproate OR Propylisopropylacetic* OR Depakene OR lamotrigine OR Triazines) OR ("Anticonvulsants"[Mesh] OR OR "Carbamazepine"[Mesh] OR "Phenytoin"[Mesh] OR "Valproic Acid"[Mesh] OR "gabapentin" [Supplementary Concept] OR "lamotrigine" [Supplementary Concept])

#8 (corticosteroid* OR GCS OR steroids OR cortisone OR corticoid* OR hydrocortisone OR antdnisone OR metacortandracin OR antdnisolone OR methylprednisolone OR triamcinolone OR hexadecadrol OR dexamethasone OR betamethasone OR metacortandralone OR methylprednisolone OR triamcinolone OR aristocort) OR ("Adrenal Cortex Hormones"[Mesh] OR "corticoid"[Mesh] OR "Steroids"[Mesh] OR "Cortisone"[Mesh] OR "Hydrocortisone"[Mesh] OR " methylprednisolone "[Mesh] OR "Triamcinolone"[Mesh])

#9 (controlled clinical trial* OR randomized controlled trial OR clinical trial* OR follow up stud* OR comparative stud* OR prospective stud* OR random allocation OR double blinds method* OR single blind method* OR evaluation stud*) OR ("Controlled Clinical Trial" [Publication Type] OR "Prospective Studies"[Mesh] OR "Evaluation Studies" [Publication Type] OR "Comparative Study" [Publication Type] OR "Follow-Up Studies"[Mesh])

#10 = (#1 AND (#2 OR #3 OR #4 OR #5 OR #6 OR #7 OR #8)) AND #9

Table S1. Search results

| Database | Citations |
| --- | --- |
| PUBMED | 2485 |
| CENTRAL | 3010 |
| EMBACE | 961 |
| WEB OF SCIENCE | 4217 |
| CLINICAL TRIAL | 566 |
| Total | 11239 |

**2.Bayes Model**

Winbugs coda of random effects model

##

Parameters: n.chains =3,n.iter=50000, n.burnin=10000, n.thin=10

y= arm means,

sd= arm standard difference,

n= arm sample size,

t= the names (numbers) of treatments,

na=a vector with the number of arms in each study,

ref=a number specifying which is the reference treatment

##

model {

for (i in 1: ns){

w[i,1] <- 0

delta[i,t[i,1]]<- 0

ss[i] <- sum(n[i, 1: na[i]])

nom[i] <- sum(nom1[i, 1: na[i]])

pooled.sd[i] <- sqrt(nom[i]/(ss[i]-na[i]))

J[i] <- 1-(3/((4 * (ss[i]-na[i]))-1))

for (k in 1: na[i]){

y[i,k] ~ dnorm(phi[i, t[i,k]],prec[i,k])

se[i,k] <- sd[i,k]/sqrt(n[i,k])

var[i,k] <- se[i,k]*se[i,k]

prec[i,k] <- 1/var[i,k]

nom1[i,k] <- (n[i,k]-1)*sd[i,k]*sd[i,k]

}

phi[i,t[i,1]] <- u[i]*(pooled.sd[i]/J[i])

for (k in 2: na[i]){

phi[i,t[i,k]] <- (u[i] + delta[i,t[i,k]])*(pooled.sd[i]/J[i])

delta[i,t[i,k]] ~ dnorm(md[i,t[i,k]], taud[i,t[i,k]])

md[i,t[i,k]] <- d[t[i,k]]-d[t[i,1]]+sw[i,k]

taud[i,t[i,k]] <- tau * 2 *(k-1)/k

w[i,k] <- (delta[i,t[i,k]]-d[t[i,k]]+d[t[i,1]])

sw[i,k] <- sum(w[i,1:k-1])/(k-1)

}

}

SD ~ dnorm(0,1)I(0,1)

tau <- 1/pow(SD,2)

for(k in 1:(ref-1)){

d[k] ~ dnorm(0,.0001)}

for(k in (ref+1):nt){

d[k] ~ dnorm(0,.0001)}

for(i in 1:ns){

u[i] ~ dnorm(0,.0001)

}

d[ref] <- 0

for (c in 1:(ref-1)){

SMD.ref[c] < -d[c]-d[ref]

predSMD.ref[c] ~ dnorm(SMD.ref[c],tau)}

for (c in (ref+1) : nt){

SMD.ref[c] < -d[c]-d[ref]

predSMD.ref[c] ~ dnorm(SMD.ref[c],tau)}

for(c in 1:(nt-1)){

for(k in (c+1):nt){

SMD[c,k] <- d[c]-d[k]

predSMD[c,k] ~ dnorm(SMD[c,k],tau)}

}

for(k in 1:nt){

order[k] <- rank(d[],k)

most.effective[k] <- equals(order[k],1)

for(j in 1:nt){

effectiveness[k,j] <- equals(order[k],j)}

}

for(k in 1:nt){

for(j in 1:nt){

cumeffectiveness[k,j] < -sum(effectiveness[k,1:j])}

}

for(k in 1:nt){

SUCRA[k] <- sum(cumeffectiveness[k,1:(nt-1)])/(nt-1)

}

for(i in 1: ns){

for(k in 1: na[i]){

Darm[i,k] <- (y[i,k]-phi[i,t[i,k]])*(y[i,k]-phi[i,t[i,k]])/var[i,k]

}

D[i] < -sum(Darm[i,1:na[i]])

}

D.bar < -sum(D[])

}

**3.Results of Pairwise Meta-Analysis**

| Table S2. Acute-LBP, Pain-Intensity | | | | | | |
| --- | --- | --- | --- | --- | --- | --- |
| **Comparison** | **trials** | **SMD** | **95%CrI** | **Chi-square** | **P-value** | **I²** |
| PLA VS NSA | 3 | 0.316 | (0.153,0.480) | 0.99 | 0.611 | 0% |
| PLA VS SMR | 5 | 0.474 | (0.354,0.594) | 52.51 | <0.001 | 92.4% |
| NSA VS OPI | 1 | 0.509 | (0.091,0.926) | NA | NA | NA |
| PLA VS ACE | 1 | 0.096 | (-0.027,0.219) | NA | NA | NA |
| NSA VS ACE | 3 | -0.026 | (-0.227,0.176) | 3.58 | 0.167 | 44.1% |
| OPI VS ACE | 1 | -0.171 | (-0.583,0.241) | NA | NA | NA |
| NSA VS C-NSA | 2 | -0.053 | (-0.242,0.135) | 2.56 | 0.109 | 61.0% |
| ACE VS TCA | 1 | 0.399 | (-0.236,1.033) | NA | NA | NA |
| OPI VS TAP | 1 | 0.000 | (-0.158,0.158) | NA | NA | NA |
| NSA VS ACE+OPI | 1 | -0.060 | (-0.415,0.295) | NA | NA | NA |
| OPI VS ACE+OPI | 1 | 0.000 | (-0.394,0.394) | NA | NA | NA |
| NSA VS NSA+SMR | 3 | 0.374 | (0.223,0.526) | 7.96 | 0.019 | 74.9% |
| ACE VS ALG | 1 | 0.161 | (-0.255,0.577) | NA | NA | NA |

| Table S3. Acute-LBP, Function-Improvement | | | | | | |
| --- | --- | --- | --- | --- | --- | --- |
| **Comparison** | **trials** | **SMD** | **95%CrI** | **Chi-square** | **P-value** | **I²** |
| PLA VS SMR | 2 | 0.265 | (0.145,0.385) | 1.40 | 0.237 | 28.6% |
| PLA VS NSA | 1 | 0.368 | (0.114,0.621) | NA | NA | NA |
| PLA VS ACE | 1 | -0.080 | (-0.203,0.044) | NA | NA | NA |
| NSA VS C-NSA | 2 | 0.097 | (-0.091,0.285) | 3.47 | 0.063 | 71.2% |
| NSA VS NSA+ACE | 1 | -0.078 | (-0.452,0.296) | NA | NA | NA |
| NSA VS NSA+DIA | 1 | 0.000 | (-0.386,0.386) | NA | NA | NA |
| NSA VS NSA+SMR | 3 | -0.035 | (-0.207,0.137) | 0.96 | 0.618 | 0% |
| NSA VS NSA+OPI+ACE | 1 | 0.123 | (-0.145,0.390) | NA | NA | NA |
| NSA+SMR VS NSA+OPI+ACE | 1 | 0.088 | (-0.179,0.355) | NA | NA | NA |

| Table S4. Chronic-LBP, Pain-Intensity | | | | | | |
| --- | --- | --- | --- | --- | --- | --- |
| **Comparison** | **trials** | **SMD** | **95%CrI** | **Chi-square** | **P-value** | **I²** |
| PLA VS NSA | 4 | 0.224 | (0.062,0.385) | 3.16 | 0.367 | 5.1% |
| PLA VS OPI | 12 | 0.452 | (0.378,0.526) | 42.51 | <0.001 | 74.1% |
| PLA VS TCA | 1 | 0.046 | (-0.546,0.637) | NA | NA | NA |
| PLA VS SSI | 2 | 0.058 | (-0.282,0.397) | 0.24 | 0.622 | 0% |
| TCA VS SSI | 1 | -0.717 | (-1.343,-0.091) | NA | NA | NA |
| PLA VS TAP | 2 | 0.337 | (0.172,0.503) | 0.06 | 0.810 | 0% |
| OPI VS TAP | 2 | 0.017 | (-0.150,0.184) | 0.05 | 0.829 | 0% |
| PLA VS C-NSA | 4 | 0.248 | (0.118,0.377) | 3.62 | 0.306 | 17.1% |
| NSA VS C-NSA | 1 | -0.085 | (-0.281,0.111) | NA | NA | NA |
| ACE VS C-NSA | 1 | 0.741 | (0.149,1.333) | NA | NA | NA |
| TCA VS ANC | 3 | -0.328 | (-0.595,-0.061) | 1.55 | 0.461 | 0% |
| SSI VS ANC | 1 | 0.144 | (-0.399,0.688) | NA | NA | NA |
| PLA VS GMA | 1 | 0.338 | (-0.232,0.908) | NA | NA | NA |
| TCA VS GMA | 1 | -0.365 | (-0.644,-0.085) | NA | NA | NA |
| C-NSA VS GMA | 1 | -0.045 | (-0.665,0.575) | NA | NA | NA |
| PLA VS ACE+TRA | 3 | 0.510 | (0.357,0.662) | 1.44 | 0.487 | 0% |
| C-NSA VS ACE+TRA | 1 | 0.689 | (0.206,1.171) | NA | NA | NA |
| PLA VS TAN | 2 | 0.352 | (0.149,0.555) | 0.10 | 0.748 | 0% |
| NSA VS TAN | 2 | 0.217 | (0.041,0.393) | 0.47 | 0.491 | 0% |
| PLA VS DUL | 4 | 0.431 | (0.321,0.542) | 8.63 | 0.035 | 65.2% |
| SSI VS DUL | 1 | 0.073 | (-0.365,0.512) | NA | NA | NA |
| C-NSA VS C-NSA+GMA | 1 | 0.889 | (0.238,1.540) | NA | NA | NA |
| GMA VS C-NSA+GMA | 1 | 0.896 | (0.244,1.547) | NA | NA | NA |
| PLA VS ANE | 1 | -0.097 | (-0.419,0.225) | NA | NA | NA |
| NSA VS ANE | 1 | -0.254 | (-0.578,0.069) | NA | NA | NA |
| PLA VS BUP | 1 | 0.334 | (0.141,0.526) | NA | NA | NA |

| Table S5. Chronic-LBP, Function-Improvement | | | | | | |
| --- | --- | --- | --- | --- | --- | --- |
| **Comparison** | **trials** | **SMD** | **95%CrI** | **Chi-square** | **P-value** | **I²** |
| PLA VS NSA | 3 | 0.162 | (-0.010,0.334) | 2.77 | 0.25 | 27.9% |
| PLA VS C-NSA | 4 | 0.662 | (0.527,0.797) | 62.05 | <0.001 | 95.2% |
| NSA VS C-NSA | 1 | 0.042 | (-0.154,0.238) | NA | NA | NA |
| PLA VS OPI | 5 | 1.163 | (1.041,1.285) | 309.24 | <0.001 | 98.7% |
| PLA VS TRA+ACE | 4 | 0.277 | (0.141,0.412) | 0.13 | 0.988 | 0% |
| PLA VS TAP | 1 | 0.214 | (-0.034,0.461) | NA | NA | NA |
| OPI VS TAP | 1 | 0.079 | (-0.168,0.325) | NA | NA | NA |
| PLA VS TAN | 2 | 0.348 | (0.145,0.552) | 0.29 | 0.590 | 0% |
| NSA VS TAN | 2 | 0.281 | (0.105,0.457) | 0.41 | 0.524 | 0% |
| PLA VS DUL | 2 | 0.157 | (0.019,0.295) | 0.30 | 0.584 | 0% |
| PLA VS SSI | 1 | 0.153 | (-0.257,0.563) | NA | NA | NA |
| C-NSA VS ACE | 1 | -0.810 | (-1.406,-0.214) | NA | NA | NA |
| PLA VS ANE | 1 | 0.178 | (-0.145,0.501) | NA | NA | NA |
| NSA VS ANE | 1 | -0.214 | (-0.537,0.110) | NA | NA | NA |
| PLA VS BUP | 1 | 0.108 | (-0.084,0.299) | NA | NA | NA |

| Table S6. Radicular-LBP, Pain-Intensity | | | | | | |
| --- | --- | --- | --- | --- | --- | --- |
| **Comparison** | **trials** | **SMD** | **95%CrI** | **Chi-square** | **P-value** | **I²** |
| PLA VS NSA | 3 | -0.016 | (-0.166,0.135) | 9.64 | 0.008 | 79.3% |
| PLA VS COR | 2 | 0.022 | (-0.218,0.262) | 0.02 | 0.881 | 0% |
| PLA VS GMA | 3 | 0.123 | (-0.062,0.308) | 1.77 | 0.413 | 0% |
| NSA VS GMA | 1 | 0.703 | (-0.013,1.418) | NA | NA | NA |
| COR VS GMA | 1 | -0.529 | (-1.160,0.102) | NA | NA | NA |
| PLA VS DUL | 1 | 0.791 | (0.214,1.367) | NA | NA | NA |
| OPI VS TAP | 1 | 0.367 | (0.106,0.628) | NA | NA | NA |
| NSA VS NSA+GMA | 3 | 0.865 | (0.517,1.214) | 6.92 | 0.031 | 71.1% |
| GMA VS NSA+GMA | 1 | 0.961 | (0.227,1.696) | NA | NA | NA |
| OPI VS TCA | 1 | 0.203 | (-0.787,1.194) | NA | NA | NA |
| OPI VS ANC | 1 | -0.160 | (-1.150,0.830) | NA | NA | NA |
| GMA VS ANC | 1 | 0.146 | (-0.369,0.662) | NA | NA | NA |
| TCA VS ANC | 1 | -0.367 | (-1.425,0.691) | NA | NA | NA |
| ANC VS ANE | 1 | 0.921 | (0.149,1.693) | NA | NA | NA |

| Table S7. Radicular-LBP, Function-Improvement | | | | | | |
| --- | --- | --- | --- | --- | --- | --- |
| **Comparison** | **trials** | **SMD** | **95%CrI** | **Chi-square** | **P-value** | **I²** |
| COR VS GMA | 2 | 0.215 | (-0.026,0.456) | 2.86 | 0.091 | 65.0% |
| PLA VS COR | 1 | -0.200 | (-0.509,0.109) | NA | NA | NA |
| PLA VS COR | 1 | -0.361 | (-0.987,0.264) | NA | NA | NA |
| OPI VS TCA | 1 | -0.109 | (-1.097,0.880) | NA | NA | NA |
| OPI VS ANC | 1 | 0.303 | (-0.215,0.821) | NA | NA | NA |
| TCA VS ANC | 1 | -0.295 | (-1.289,0.698) | NA | NA | NA |
| ANC VS ANE | 1 | -0.184 | (-1.234,0.866) | NA | NA | NA |
| PLA VS GMA | 1 | 0.129 | (-0.603,0.862) | NA | NA | NA |

**4.** **Local Inconsistency**

We used the tests of local inconsistency revealed that the percentages of inconsistent loops were to be expected according to empirical data

| Table S8. Acute LBP, Pain Intensity | | | | | |
| --- | --- | --- | --- | --- | --- |
| **Loop** | **ROR** | **Z-value** | **P-value** | **95%CI** | **τ²** |
| NSA-OPI-TAP | 1.722 | 1.581 | 0.114 | (1.000,3.380) | 0.000 |
| NSA-OPI-ACE | 1.558 | 1.384 | 0.166 | (1.000,2.920) | 0.000 |
| PLA-NSA-ACE | 1.228 | 1.153 | 0.249 | (1.000,1.740) | 0.005 |

| Table S9. Acute LBP, Function Improvement | | | | | |
| --- | --- | --- | --- | --- | --- |
| **Loop** | **ROR** | **Z-value** | **P-value** | **95%CI** | **τ²** |
| NSA-NSA+DIA-NSA+SMR | 1.112 | 0.473 | 0.636 | (1.000,1.730) | 0.000 |

| Table S10. Chronic LBP, Pain Intensity | | | | | |
| --- | --- | --- | --- | --- | --- |
| **Loop** | **ROR** | **Z-value** | **P-value** | **95%CI** | **τ²** |
| PLA-TCA-SSI | 2.209 | 1.686 | 0.092 | (1.000,5.550) | 0.000 |
| PLA-TCA-GMA | 1.918 | 1.471 | 0.141 | (1.000,4.570) | 0.000 |
| PLA-C_NSA-ACE+TRA | 1.521 | 1.569 | 0.117 | (1.000,2.570) | 0.000 |
| TCA-SSI-ANC | 1.518 | 0.932 | 0.351 | (1.000,3.650) | 0.000 |
| PLA-SSI-DUL | 1.321 | 0.806 | 0.420 | (1.000,2.600) | 0.020 |
| PLA-OPI-TAP | 1.272 | 0.906 | 0.365 | (1.000,2.140) | 0.045 |
| PLA-NSA-TAN | 1.156 | 0.753 | 0.451 | (1.000,1.690) | 0.000 |
| PLA-C_NSA-GMA | 1.130 | 0.274 | 0.784 | (1.000,2.700) | 0.004 |
| PLA-NSA-C_NSA | 1.124 | 0.726 | 0.468 | (1.000,1.540) | 0.003 |
| PLA-NSA-BUP | 1.082 | 0.312 | 0.755 | (1.000,1.770) | 0.000 |
| Loop C_NSA-GMA-C_NSA+GMA is formed only by multi-arm trial - Consistent by definition | | | | | |

| Table S11. Chronic LBP, Function Improvement | | | | | |
| --- | --- | --- | --- | --- | --- |
| **Loop** | **ROR** | **Z-value** | **P-value** | **95%CI** | **τ²** |
| PLA-OPI-TAP | 2.672 | 0.519 | 0.604 | (1.000,9.640) | 1.579 |
| PLA-NSA-C_NSA | 1.644 | 0.740 | 0.460 | (1.000,6.130) | 0.269 |
| PLA-NSA-TAN | 1.389 | 1.530 | 0.126 | (1.000,2.120) | 0.000 |
| PLA-NSA-ACE | 1.378 | 1.259 | 0.208 | (1.000,2.270) | 0.000 |

| Table S12. Radicular LBP, Pain Intensity | | | | | |
| --- | --- | --- | --- | --- | --- |
| **Loop** | **ROR** | **Z-value** | **P-value** | **95%CI** | **τ²** |
| NSA-GMA-NSA+GMA | 2.598 | 1.729 | 0.084 | (1.00,7.67) | 0.000 |
| PLA-COR-GMA | 1.856 | 1.731 | 0.084 | (1.00,3.74) | 0.000 |
| PLA-NSA-GMA | 1.709 | 1.134 | 0.257 | (1.00,4.31) | 0.028 |
| Loop OPI-TCA-ANC is formed only by multi-arm trial - Consistent by definition | | | | | |

| Table S13. Radicular LBP, Function Improvement | | | | | |
| --- | --- | --- | --- | --- | --- |
| **Loop** | **ROR** | **Z-value** | **P-value** | **95%CI** | **τ²** |
| PLA-COR-ANT | 1.061 | 0.158 | 0.875 | (1.000,2.220) | 0.000 |
| Loop OPI-TCA-ANC is formed only by multi-arm trial - Consistent by definition | | | | | |

**5.** **Node-splitting Inconsistency**

Tests of inconsistency using node-splitting method fitted the node-splitting model. The results reported the estimated direct and indirect treatment effects and their differences; the P-value of the difference is the test of consistency.

| Table S14. Acute LBP, Pain Intensity | | | | | | | | |
| --- | --- | --- | --- | --- | --- | --- | --- | --- |
| **Comparison** | **Direct** | | **Indirect** | | **Difference** | | | **tau** |
|  | **SMD** | **SE** | **SMD** | **SE** | **SMD** | **SE** | **P-value** |  |
| PLA VS NSA | -0.338 | 0.231 | -0.125 | 0.438 | -0.213 | 0.496 | 0.667 | 0.365 |
| PLA VS SMR | . | . | . | . | . | . | . | . |
| PLA VS ACE | -0.096 | 0.370 | -0.308 | 0.330 | 0.212 | 0.496 | 0.669 | 0.365 |
| NSA VS OPI | -0.484 | 0.404 | 0.201 | 0.463 | -0.685 | 0.614 | 0.265 | 0.344 |
| NSA VS ACE | 0.007 | 0.237 | 0.291 | 0.413 | -0.283 | 0.476 | 0.552 | 0.362 |
| NSA VS C-NSA | 0.133 | 0.270 | 0.585 | 44.720 | -0.452 | 44.721 | 0.992 | 0.348 |
| NSA VS ACE+OPI | 0.060 | 0.404 | -0.294 | 0.559 | 0.354 | 0.690 | 0.608 | 0.361 |
| NSA VS NSA+SMR | -0.379 | 0.219 | 0.590 | 36.513 | -0.969 | 36.514 | 0.979 | 0.348 |
| OPI VS ACE | 0.167 | 0.419 | 0.442 | 0.557 | -0.274 | 0.697 | 0.694 | 0.363 |
| OPI VS TAP | 0.000 | 0.357 | 0.959 | 63.232 | -0.959 | 63.233 | 0.988 | 0.348 |
| OPI VS ACE+OPI | 0.000 | 0.413 | 0.354 | 0.552 | -0.354 | 0.690 | 0.608 | 0.361 |
| ACE VS TCA | -0.390 | 0.475 | 0.436 | 63.254 | -0.827 | 63.256 | 0.990 | 0.348 |
| ACE VS ALG | -0.159 | 0.407 | 0.424 | 63.241 | -0.584 | 63.242 | 0.993 | 0.348 |

| Table S15. Acute LBP, Function Improvement | | | | | | | | |
| --- | --- | --- | --- | --- | --- | --- | --- | --- |
| **Comparison** | **Direct** | | **Indirect** | | **Difference** | | | **tau** |
|  | **SMD** | **SE** | **SMD** | **SE** | **SMD** | **SE** | **P-value** |  |
| PLA VS SMR | . | . | . | . | . | . | . | . |
| PLA VS NSA | -0.367 | 0.149 | 0.008 | 11.950 | -0.374 | 11.951 | 0.975 | 0.075 |
| PLA VS ACE | . | . | . | . | . | . | . | . |
| NSA VS C-NSA | -0.117 | 0.122 | 0.733 | 44.711 | -0.850 | 44.711 | 0.985 | 0.075 |
| NSA VS NSA+ACE | 0.078 | 0.205 | 0.730 | 63.272 | -0.652 | 63.273 | 0.992 | 0.075 |
| NSA VS NSA+DIA | 0.000 | 0.211 | 0.733 | 63.171 | -0.733 | 63.171 | 0.991 | 0.075 |
| NSA VS NSA+SMR | 0.037 | 0.098 | 0.756 | 36.501 | -0.719 | 36.501 | 0.984 | 0.075 |
| NSA VS NSA+OPI+ACE | -0.118 | 0.171 | 0.093 | 0.402 | -0.212 | 0.438 | 0.629 | 0.104 |
| NSA+SMR VS NSA+OPI+ACE | -0.091 | 0.171 | -0.303 | 0.403 | 0.212 | 0.438 | 0.629 | 0.104 |

| Table S16. Chronic LBP, Pain Intensity | | | | | | | | |
| --- | --- | --- | --- | --- | --- | --- | --- | --- |
| **Comparison** | **Direct** | | **Indirect** | | **Difference** | | | **tau** |
|  | **SMD** | **SE** | **SMD** | **SE** | **SMD** | **SE** | **P-value** |  |
| PLA VS NSA | -0.233 | 0.119 | -0.308 | 0.209 | 0.075 | 0.241 | 0.755 | 0.156 |
| PLA VS OPI | . | . | . | . | . | . | . | . |
| PLA VS TCA | -0.045 | 0.337 | -0.679 | 0.234 | 0.635 | 0.410 | 0.122 | 0.149 |
| PLA VS SSI | -0.065 | 0.207 | -0.133 | 0.231 | 0.067 | 0.311 | 0.828 | 0.154 |
| PLA VS TAP | -0.330 | 0.135 | -0.778 | 0.274 | 0.448 | 0.306 | 0.142 | 0.149 |
| PLA VS C-NSA | -0.268 | 0.103 | -0.067 | 0.164 | -0.201 | 0.194 | 0.300 | 0.152 |
| PLA VS GMA | -0.333 | 0.328 | -0.086 | 0.240 | -0.246 | 0.407 | 0.545 | 0.153 |
| PLA VS ACE+TRA | -0.491 | 0.124 | -0.925 | 0.303 | 0.434 | 0.327 | 0.185 | 0.151 |
| PLA VS TAN | -0.385 | 0.159 | -0.724 | 0.375 | 0.340 | 0.416 | 0.414 | 0.154 |
| PLA VS DUL | -0.414 | 0.095 | -0.069 | 0.325 | -0.346 | 0.339 | 0.308 | 0.151 |
| PLA VS ANE | 0.098 | 0.226 | -0.137 | 0.455 | 0.234 | 0.508 | 0.645 | 0.155 |
| PLA VS BUP | . | . | . | . | . | . | . | . |
| NSA VS C-NSA | 0.085 | 0.186 | 0.010 | 0.153 | 0.075 | 0.241 | 0.755 | 0.156 |
| NSA VS TAN | -0.232 | 0.146 | 0.108 | 0.390 | -0.340 | 0.416 | 0.414 | 0.154 |
| NSA VS ANE | 0.256 | 0.226 | 0.491 | 0.455 | -0.234 | 0.508 | 0.645 | 0.155 |
| OPI VS TAP | -0.019 | 0.136 | 0.430 | 0.273 | -0.448 | 0.306 | 0.142 | 0.149 |
| TCA VS SSI | 0.777 | 0.350 | 0.150 | 0.262 | 0.628 | 0.440 | 0.154 | 0.150 |
| TCA VS ANC | 0.341 | 0.166 | -0.460 | 0.816 | 0.800 | 0.840 | 0.340 | 0.153 |
| TCA VS GMA | 0.363 | 0.209 | 0.128 | 0.344 | 0.236 | 0.403 | 0.558 | 0.153 |
| SSI VS ANC | -0.092 | 0.316 | -0.052 | 0.340 | -0.040 | 0.461 | 0.931 | 0.154 |
| SSI VS DUL | -0.073 | 0.270 | -0.418 | 0.205 | 0.346 | 0.339 | 0.308 | 0.151 |
| ACE VS C-NSA | -0.728 | 0.338 | -0.438 | 63.250 | -0.290 | 63.251 | 0.996 | 0.151 |
| C-NSA VS GMA | 0.043 | 0.352 | 0.037 | 0.247 | 0.006 | 0.429 | 0.989 | 0.154 |
| C-NSA VS ACE+TRA | -0.680 | 0.289 | -0.247 | 0.154 | -0.434 | 0.327 | 0.185 | 0.151 |
| C-NSA VS C-NSA+GMA | -0.872 | 0.361 | -0.883 | 0.789 | 0.012 | 0.859 | 0.989 | 0.154 |
| GMA VS C-NSA+GMA | -0.915 | 0.362 | -0.903 | 0.788 | -0.012 | 0.859 | 0.989 | 0.154 |

| Table S17. Chronic LBP, Function Improvement | | | | | | | | |
| --- | --- | --- | --- | --- | --- | --- | --- | --- |
| **Comparison** | **Direct** | | **Indirect** | | **Difference** | | | **tau** |
|  | **SMD** | **SE** | **SMD** | **SE** | **SMD** | **SE** | **P-value** |  |
| PLA VS NSA | -0.178 | 0.388 | -0.676 | 0.736 | 0.498 | 0.832 | 0.549 | 0.649 |
| PLA VS C-NSA | -0.718 | 0.333 | -0.220 | 0.763 | -0.498 | 0.832 | 0.549 | 0.649 |
| PLA VS OPI | . | . | . | . | . | . | . | . |
| PLA VS TRA+ACE | . | . | . | . | . | . | . | . |
| PLA VS TAP | -0.207 | 0.623 | -2.205 | 1.250 | 1.998 | 1.397 | 0.153 | 0.610 |
| PLA VS TAN | -0.383 | 0.476 | -1.286 | 1.280 | 0.903 | 1.370 | 0.510 | 0.646 |
| PLA VS DUL | . | . | . | . | . | . | . | . |
| PLA VS SSI | . | . | . | . | . | . | . | . |
| PLA VS ANE | -0.176 | 0.676 | 0.111 | 1.424 | -0.287 | 1.576 | 0.856 | 0.656 |
| PLA VS BUP | . | . | . | . | . | . | . | . |
| NSA VS C-NSA | -0.042 | 0.656 | -0.540 | 0.511 | 0.498 | 0.832 | 0.549 | 0.649 |
| NSA VS TAN | -0.314 | 0.469 | 0.589 | 1.288 | -0.903 | 1.370 | 0.510 | 0.646 |
| NSA VS ANE | 0.217 | 0.676 | -0.070 | 1.423 | 0.287 | 1.576 | 0.856 | 0.656 |
| C-NSA VS ACE | 0.797 | 0.703 | 2.074 | 200.228 | -1.277 | 200.229 | 0.995 | 0.634 |
| OPI VS TAP | -0.079 | 0.623 | 1.919 | 1.250 | -1.998 | 1.397 | 0.153 | 0.610 |

| Table S18. Radicular LBP, Pain Intensity | | | | | | | | |
| --- | --- | --- | --- | --- | --- | --- | --- | --- |
| **Comparison** | **Direct** | | **Indirect** | | **Difference** | | | **tau** |
|  | **SMD** | **SE** | **SMD** | **SE** | **SMD** | **SE** | **P-value** |  |
| PLA VS NSA | -0.043 | 0.174 | 0.261 | 0.434 | -0.304 | 0.468 | 0.516 | 0.263 |
| PLA VS COR | -0.016 | 0.208 | -0.693 | 0.421 | 0.677 | 0.470 | 0.150 | 0.224 |
| PLA VS GMA | -0.149 | 0.182 | 0.028 | 0.321 | -0.177 | 0.368 | 0.631 | 0.262 |
| PLA VS DUL | . | . | . | . | . | . | . | . |
| NSA VS GMA | -0.648 | 0.433 | 0.041 | 0.226 | -0.688 | 0.492 | 0.162 | 0.240 |
| NSA VS NSA+GMA | -0.918 | 0.237 | -0.310 | 0.906 | -0.609 | 0.937 | 0.516 | 0.263 |
| COR VS GMA | 0.518 | 0.392 | -0.159 | 0.259 | 0.677 | 0.470 | 0.150 | 0.224 |
| OPI VS TAP | -0.366 | 0.276 | 0.816 | 63.227 | -1.182 | 63.228 | 0.985 | 0.241 |
| OPI VS TCA | -0.194 | 0.560 | -1.376 | 63.262 | 1.182 | 63.264 | 0.985 | 0.241 |
| OPI VS ANC | 0.155 | 0.559 | -0.484 | 28.288 | 0.639 | 28.294 | 0.982 | 0.241 |
| GMA VS NSA+GMA | -0.904 | 0.453 | -0.693 | 0.365 | -0.211 | 0.581 | 0.717 | 0.264 |
| GMA VS ANC | -0.144 | 0.357 | 0.393 | 17.545 | -0.537 | 17.549 | 0.976 | 0.241 |
| TCA VS ANC | 0.350 | 0.589 | -0.658 | 44.744 | 1.007 | 44.748 | 0.982 | 0.241 |
| ANC VS ANE | -0.894 | 0.463 | 0.405 | 63.243 | -1.300 | 63.245 | 0.984 | 0.241 |

| Table S19. Radicular LBP, Function Improvement | | | | | | | | |
| --- | --- | --- | --- | --- | --- | --- | --- | --- |
| **Comparison** | **Direct** | | **Indirect** | | **Difference** | | | **tau** |
|  | **SMD** | **SE** | **SMD** | **SE** | **SMD** | **SE** | **P-value** |  |
| PLA VS COR | -0.129 | 0.250 | -0.155 | 0.531 | 0.026 | 0.587 | 0.964 | 0.279 |
| PLAGMA | 0.199 | 0.320 | 0.225 | 0.492 | -0.026 | 0.587 | 0.964 | 0.279 |
| COR VS GMA | 0.354 | 0.424 | 0.328 | 0.406 | 0.026 | 0.587 | 0.964 | 0.279 |
| GMA VS ANC | -0.299 | 0.305 | -0.126 | 66.673 | -0.173 | 66.674 | 0.998 | 0.153 |
| OPI VS TCA | . | . | . | . | . | . | . | . |
| OPI VS ANC | 0.281 | 0.529 | -0.487 | 200.007 | 0.768 | 200.009 | 0.997 | 0.153 |
| TCA VS ANC | 0.176 | 0.557 | -0.593 | 199.938 | 0.768 | 199.938 | 0.997 | 0.153 |
| ANC VS ANE | -0.126 | 0.404 | 0.080 | 200.484 | -0.206 | 200.484 | 0.999 | 0.153 |

**6.Funnel Plots**

Figure S1. Acute LBP, Pain Intensity


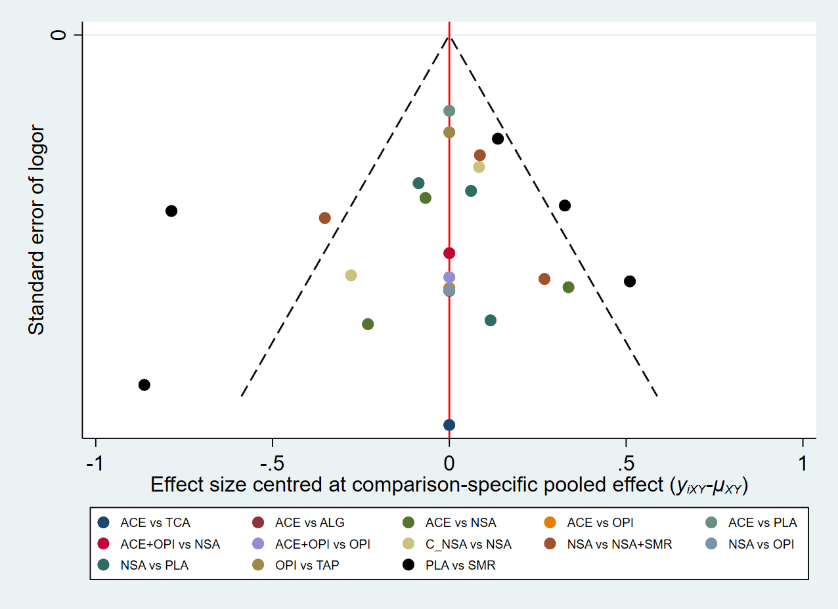


Figure S2. Acute LBP, Function Improvement


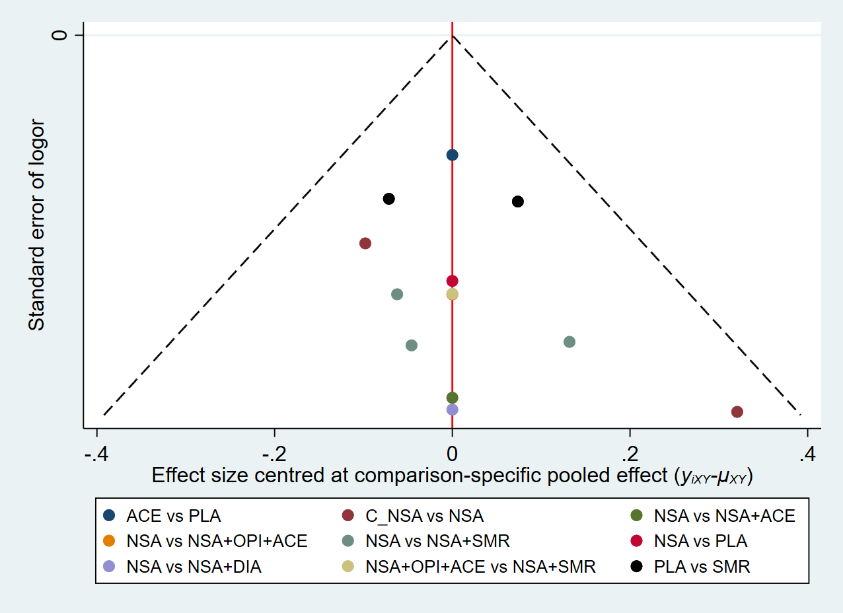


Figure S3. Chronic LBP, Pain Intensity


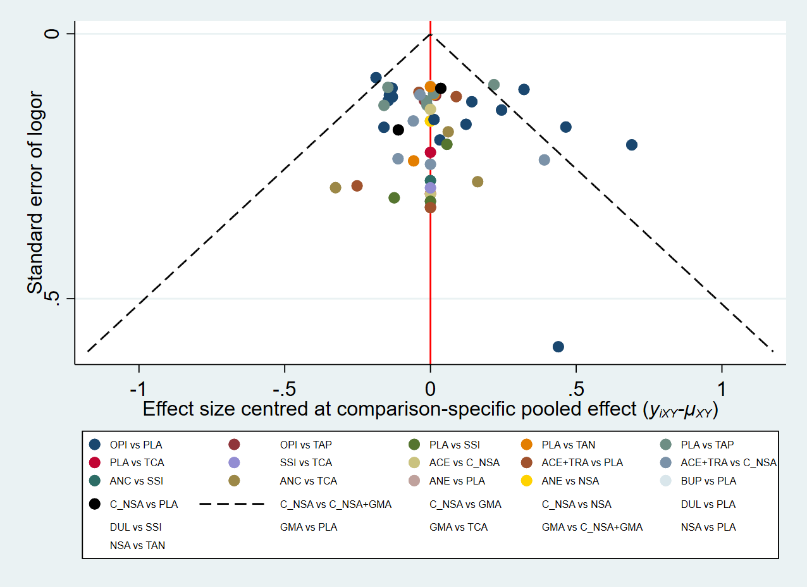


Figure S4. Chronic LBP, Function Improvement


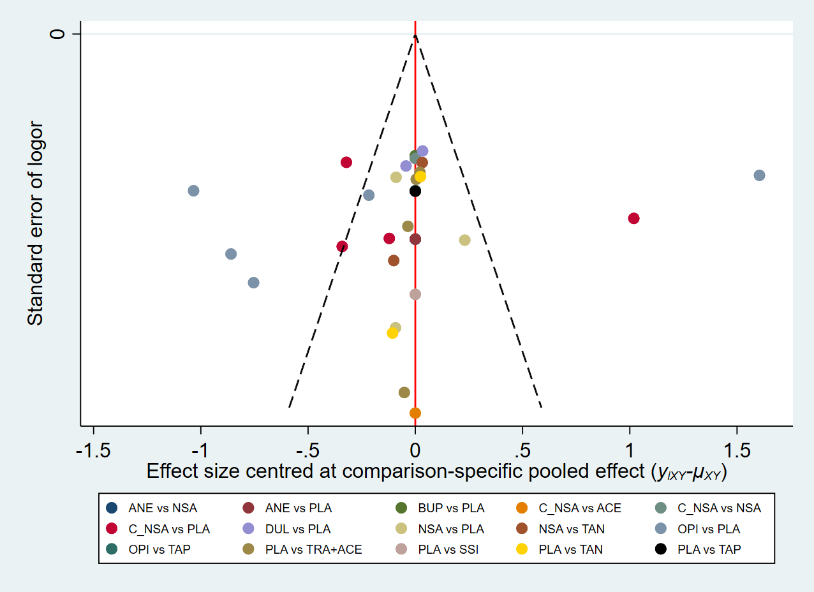


Figure S5. Radicular LBP, Pain Intensity


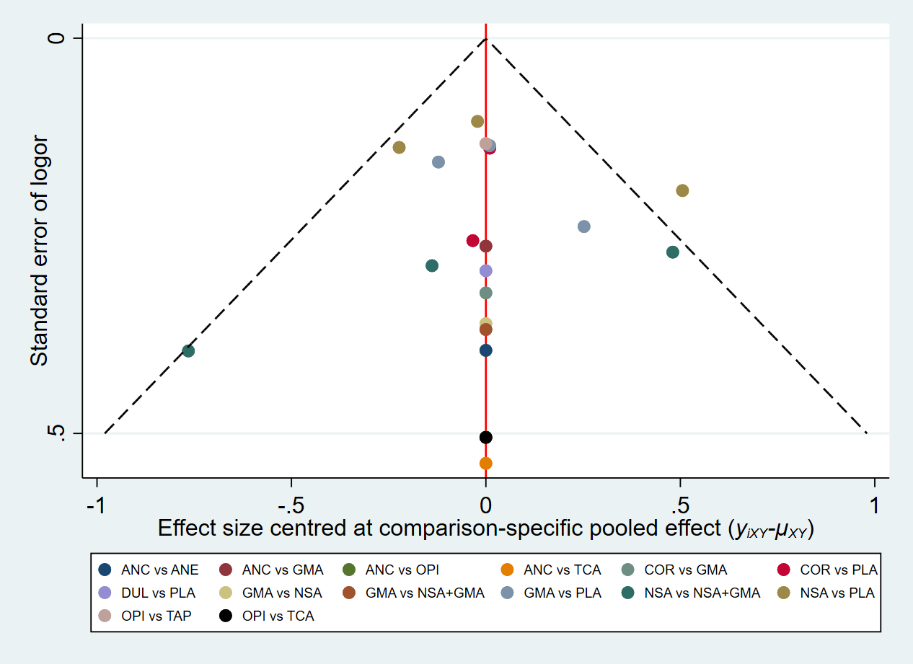


Figure S6. Radicular LBP, Function Improvement


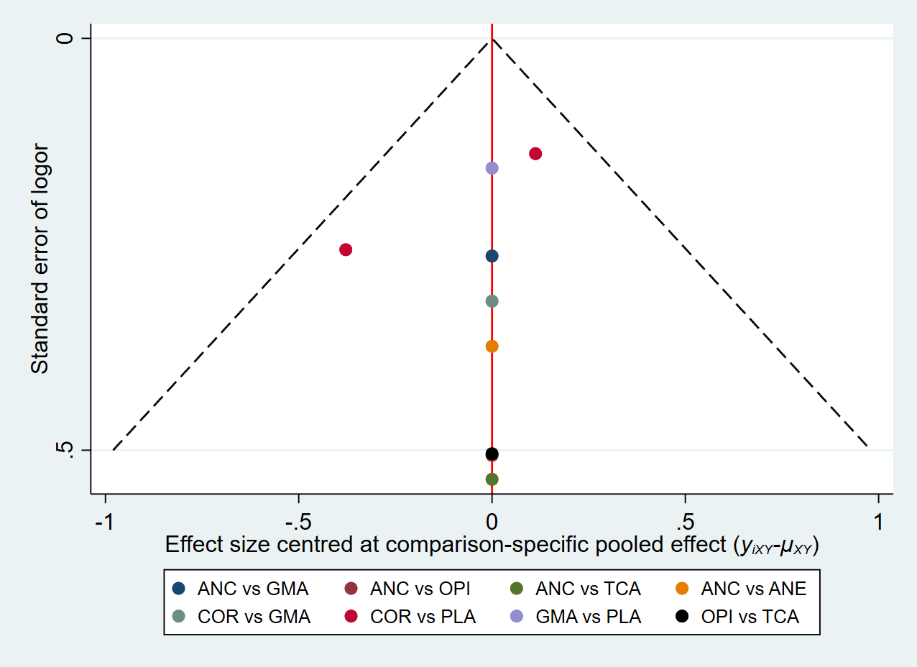


**7. Risk of Bias, Included Studies**

Figure S7.1. Figure S7.2.


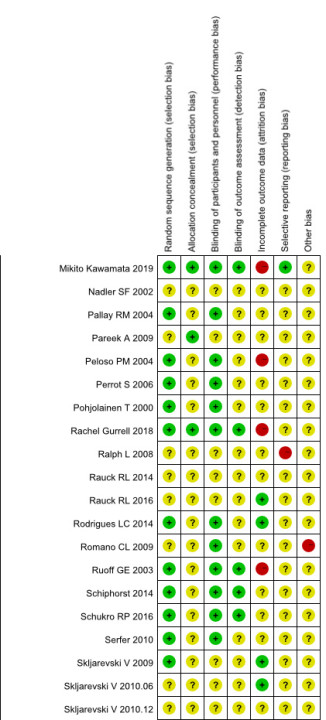

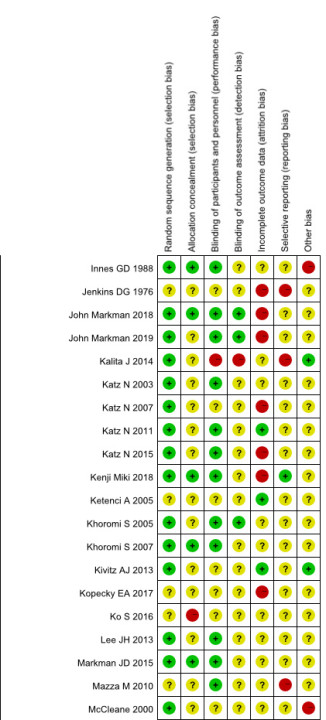


Figure S7.3
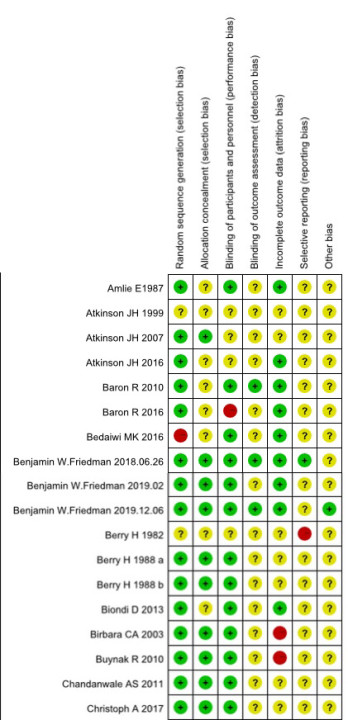
. Figure S7.4.


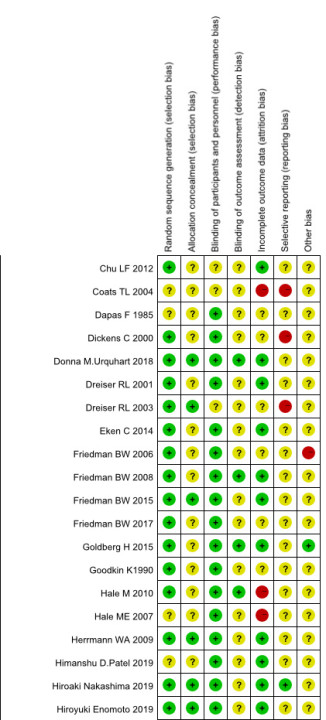


Figure S7.5.


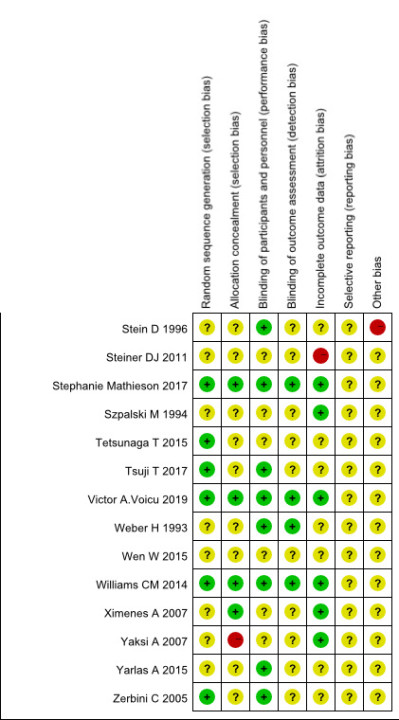


Figure S7.6.


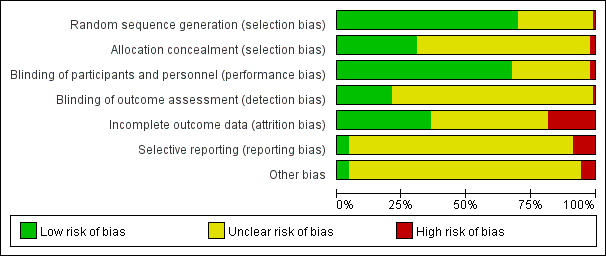


**8. Risk of Bias, Direct Comparisons**

Figure S8.

**9. Contribution Matrix**

This matrix reports the contribution of each direct comparison to the mixed comparison, indirect comparison, and the whole network.

Figure S9. Acute LBP, Pain Intensity


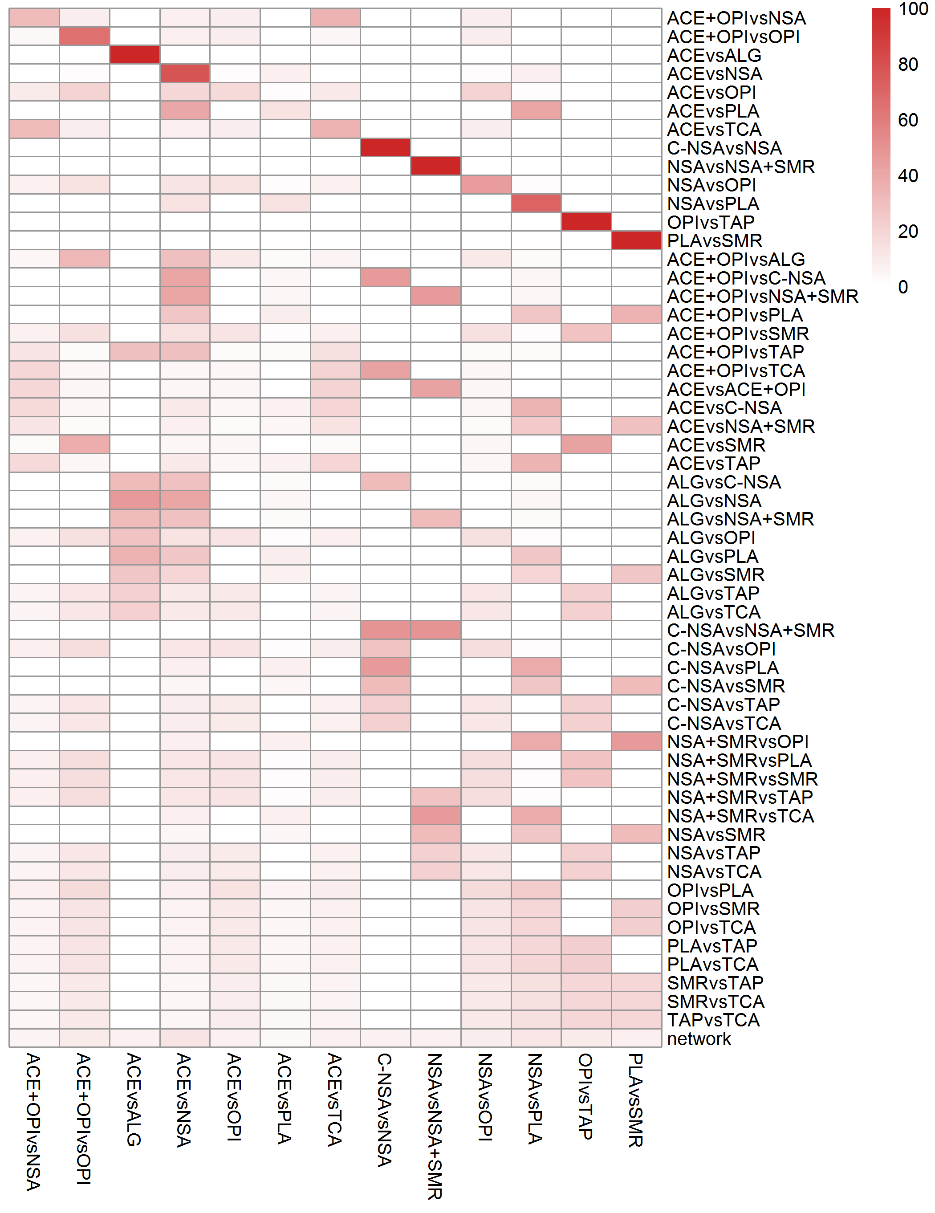


Figure S10. Acute LBP, Function Improvement


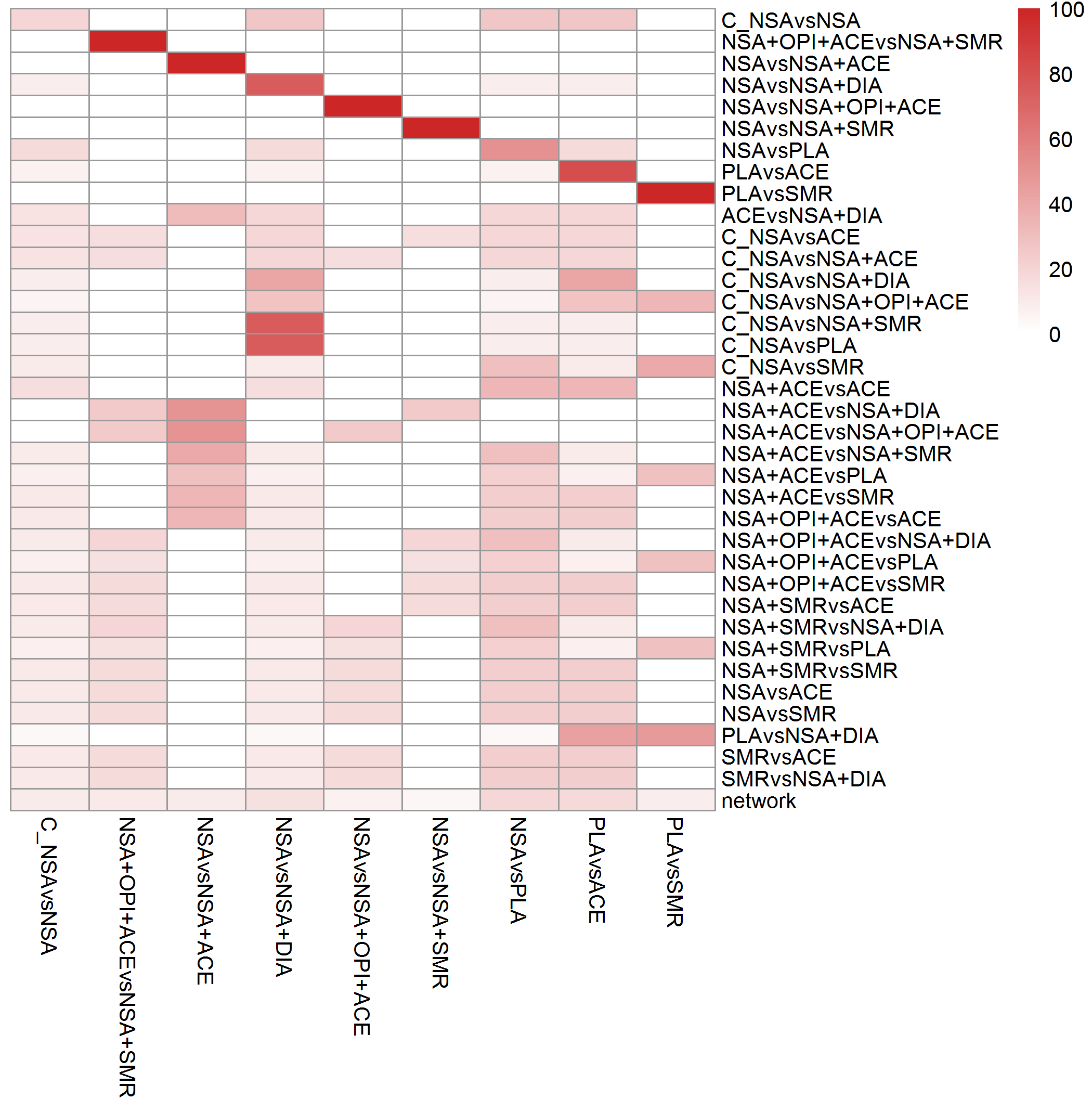


Figure S11. Chronic LBP, Pain Intensity


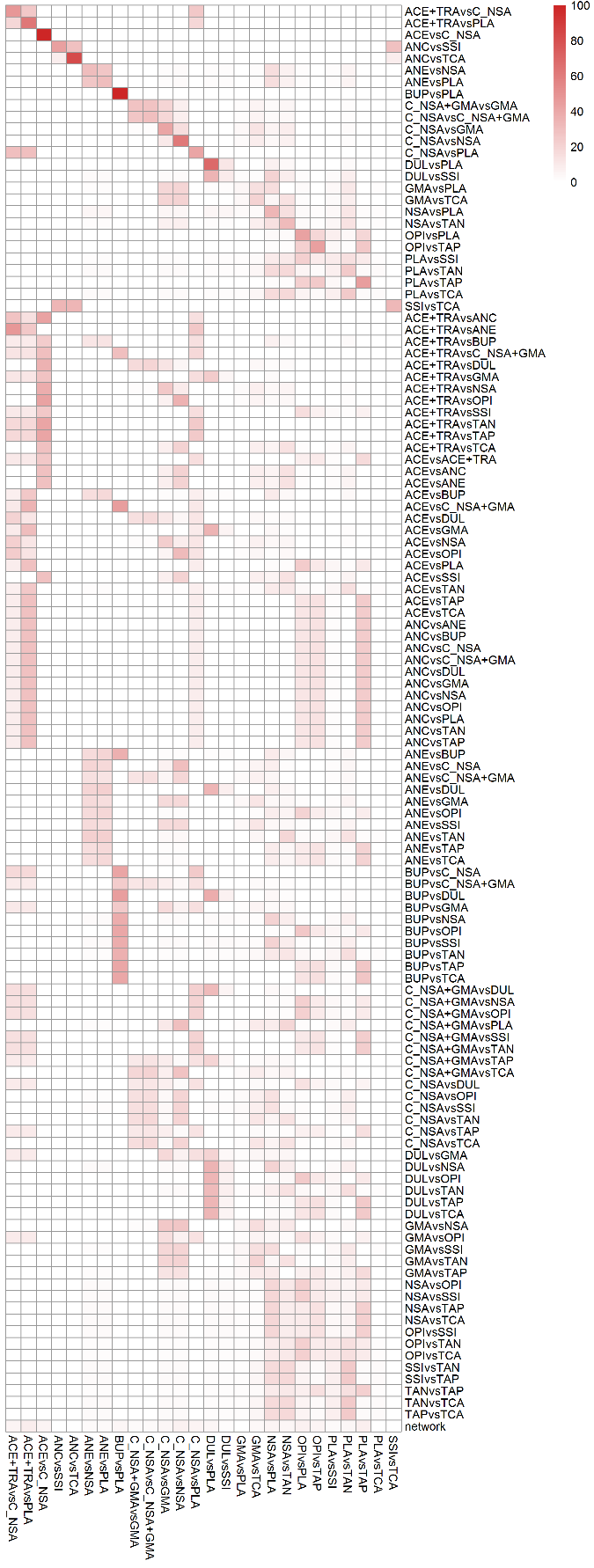


Figure S12. Chronic LBP, Function Improvement


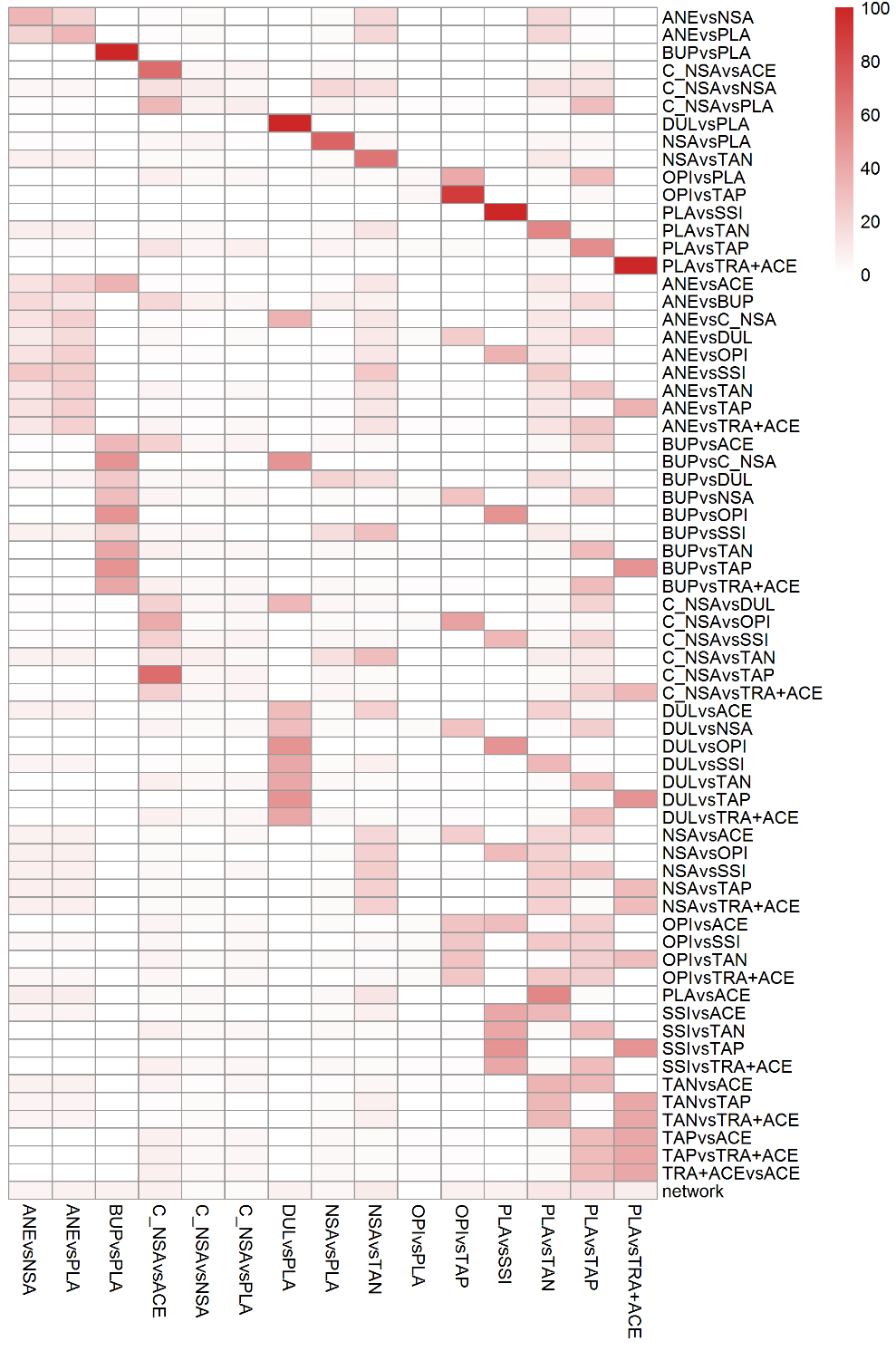


Figure S13. Radicular LBP, Pain Intensity


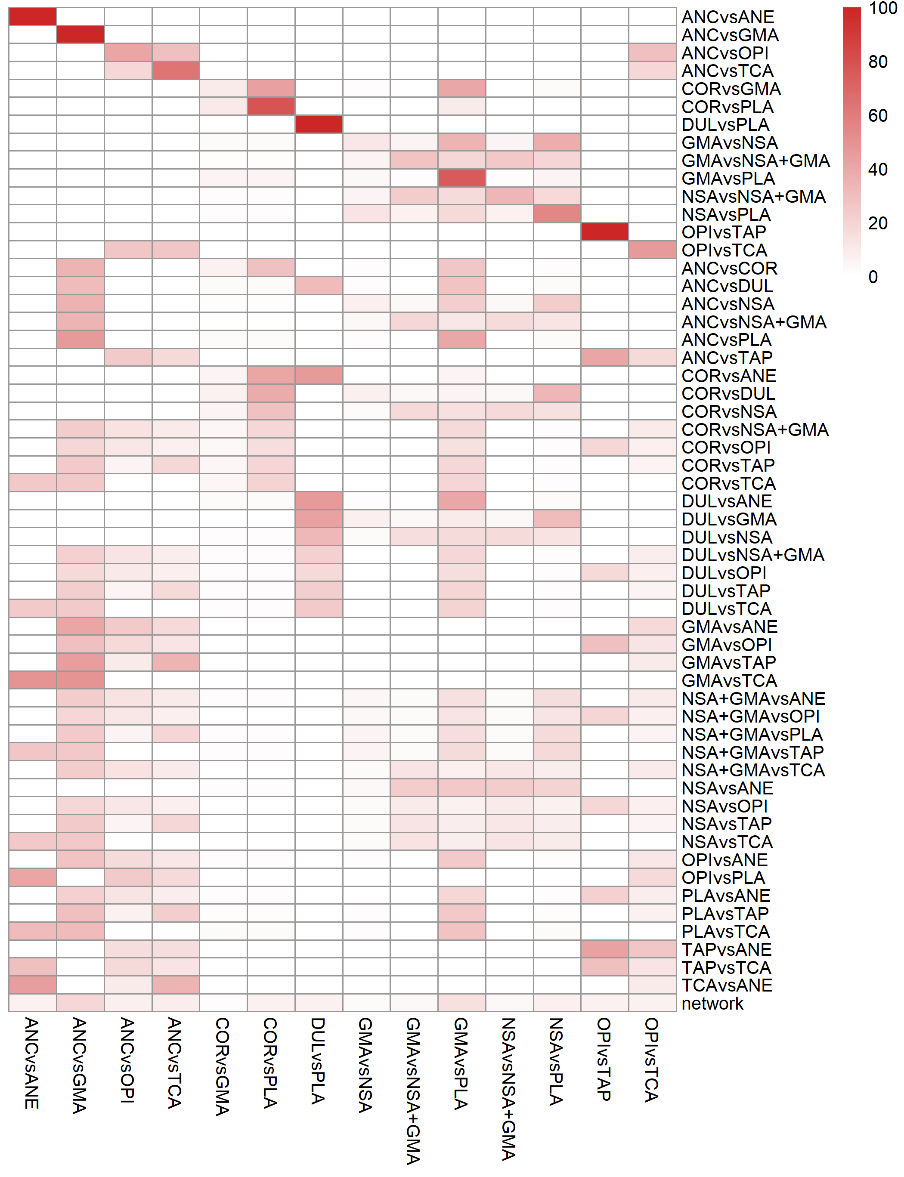


Figure S14. Radicular LBP, Function Improvement


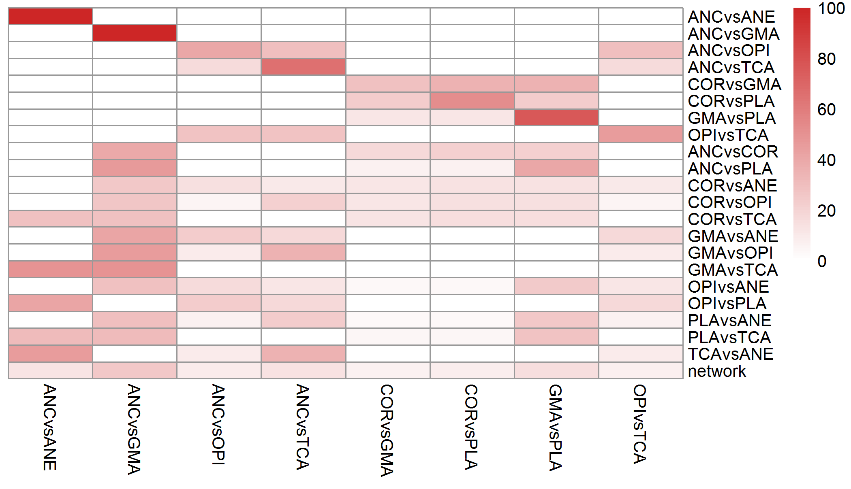


**10. Risk of Bias, Whole Network**

Figure S15. Acute LBP, Pain Intensity


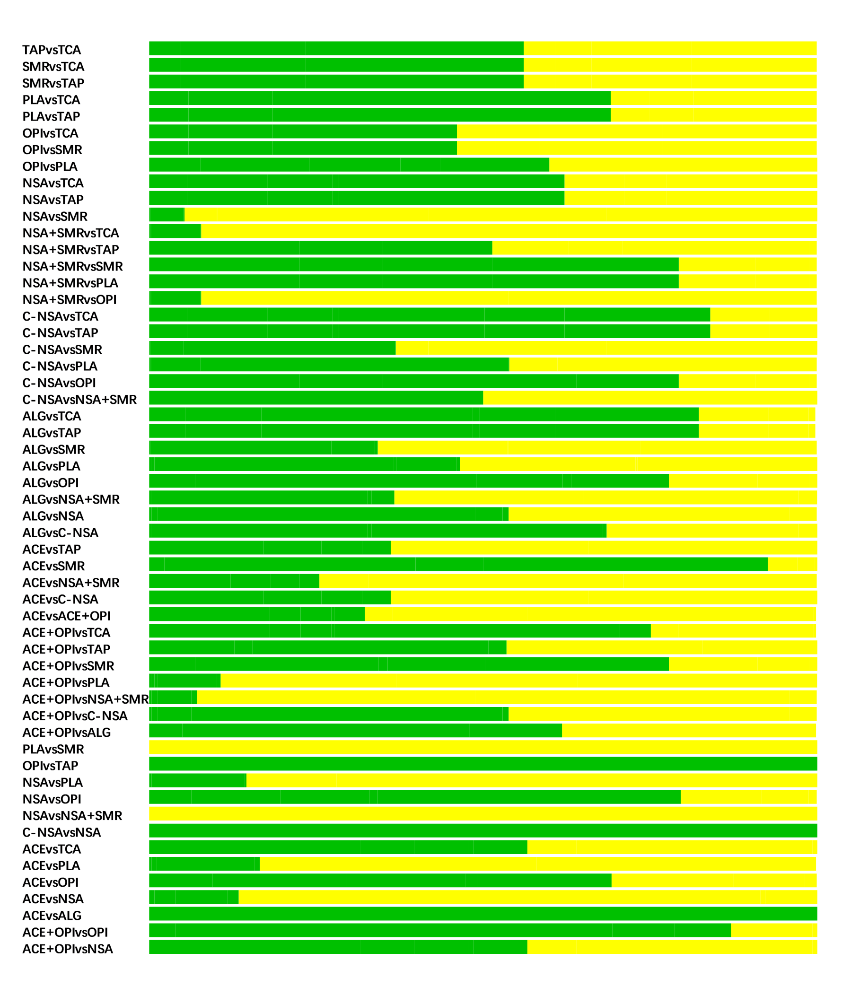


Figure S16. Acute LBP, Function Improvement


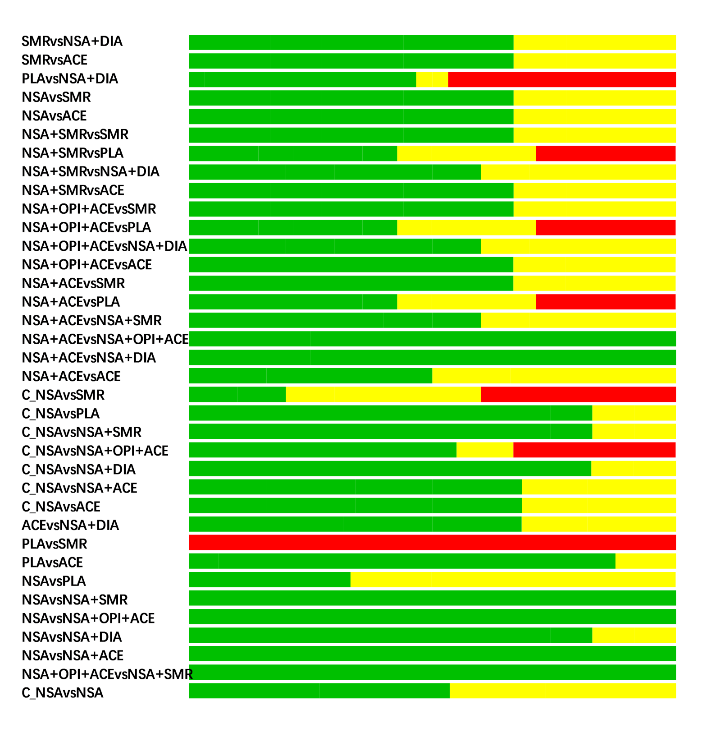


Chronic LBP, Pain Intensity

Figure S17A.


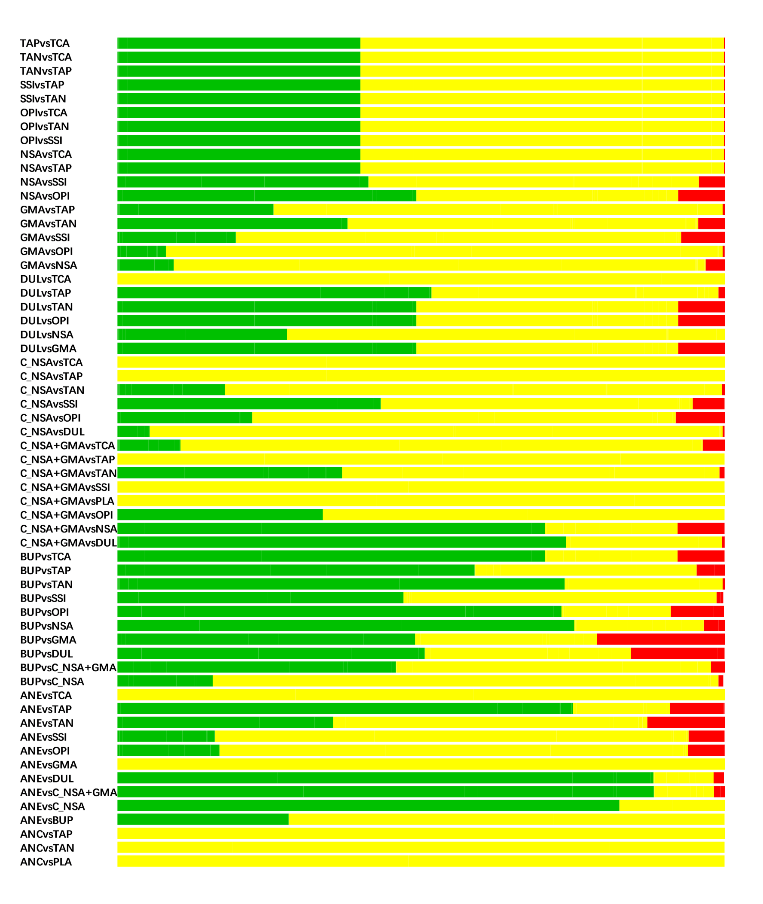


Figure S17B.


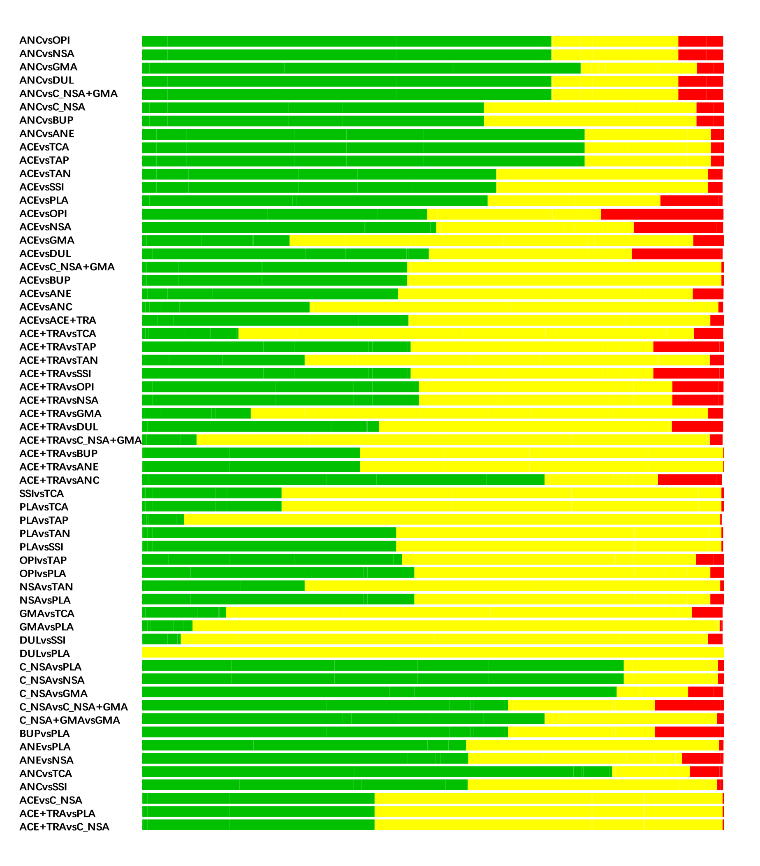


Figure S18. Chronic LBP, Function Improvement


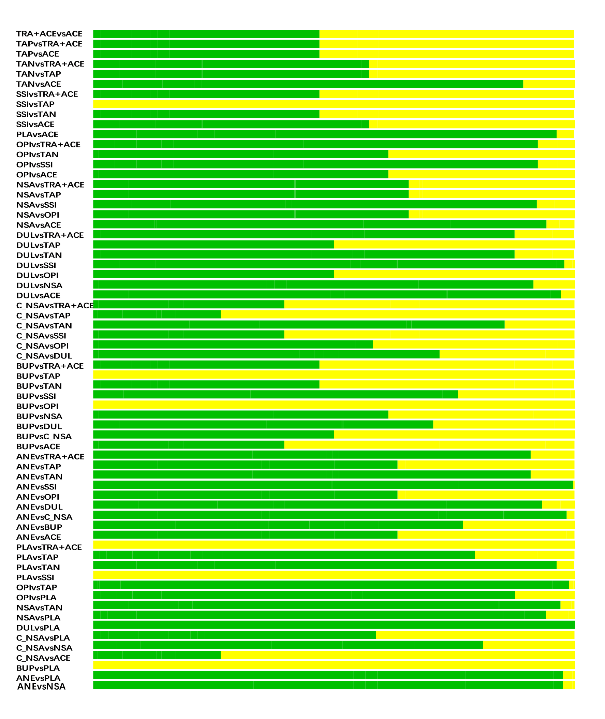


Figure S19. Radicular LBP, Pain Intensity


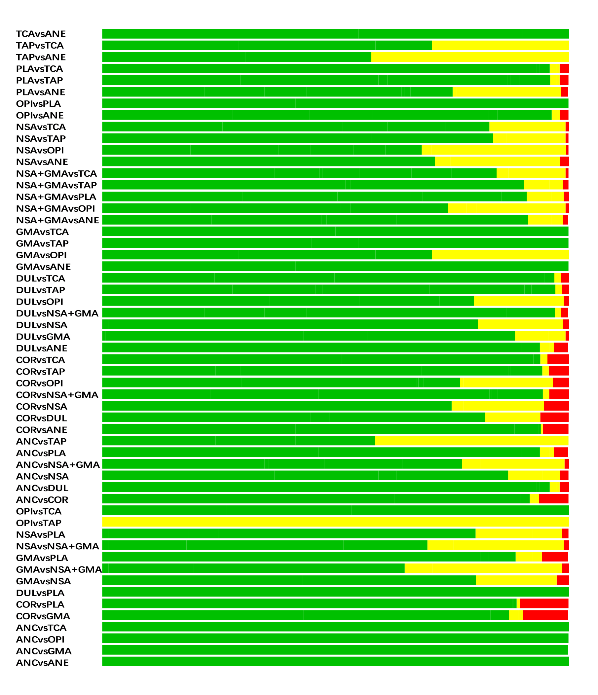


Figure S20. Radicular LBP, Function Improvement


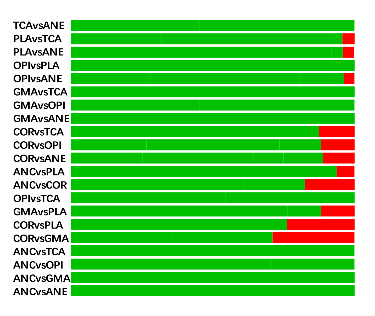


**11. Result of Network Meta-Analysis and Sensitivity Analysis**

|  | Complete model |
| --- | --- |
|  | Omitting the low-quality trials |

Omitted trials included, Berry H (1982)^8^, Coats TL (2004)^17^, Jenkins DG (1976)^36^, Kalita J (2014)^37^, Ko S (2016)^47^, Kopecky EA (2017)^48^, Ralph L (2008)^65^.

Table S20. Acute LBP, Pain Intensity

| PLA | 0.29 | **0.66** | 0.48 | 0.21 | 0.15 | 0.6 | 0.49 | 0.35 | 0.67 | 0.36 |
| --- | --- | --- | --- | --- | --- | --- | --- | --- | --- | --- |
|  | (-0.17,0.74) | **(0.2,1.12)** | (-0.32,1.3) | (-0.37,0.78) | (-0.64,0.94) | (-0.59,1.79) | (-0.67,1.67) | (-0.5,1.21) | (-0.02,1.35) | (-0.75,1.47) |
| 0.3 | NSA | 0.37 | 0.19 | -0.08 | -0.13 | 0.31 | 0.2 | 0.07 | 0.38 | 0.07 |
| (-0.14,0.75) |  | (-0.27,1.02) | (-0.51,0.91) | (-0.54,0.4) | (-0.77,0.49) | (-0.83,1.46) | (-0.89,1.3) | (-0.65,0.79) | (-0.13,0.89) | (-0.99,1.15) |
| **0.58** | 0.28 | SMR | -0.17 | -0.45 | -0.5 | -0.06 | -0.17 | -0.3 | 0.01 | -0.3 |
| **(0.2,0.97)** | (-0.31,0.87) |  | (-1.1,0.77) | (-1.18,0.28) | (-1.42,0.4) | (-1.34,1.23) | (-1.43,1.09) | (-1.28,0.66) | (-0.81,0.83) | (-1.5,0.9) |
| 0.49 | 0.19 | -0.09 | OPI | -0.28 | -0.33 | 0.11 | 0 | -0.13 | 0.19 | -0.12 |
| (-0.31,1.28) | (-0.5,0.87) | (-0.98,0.8) |  | (-1.01,0.47) | (-1.29,0.62) | (-1.15,1.41) | (-0.84,0.86) | (-0.87,0.62) | (-0.68,1.06) | (-1.33,1.08) |
| 0.22 | -0.08 | -0.36 | -0.27 | ACE | -0.05 | 0.39 | 0.28 | 0.15 | 0.46 | 0.15 |
| (-0.32,0.75) | (-0.53,0.37) | (-1.02,0.3) | (-1,0.45) |  | (-0.87,0.74) | (-0.66,1.45) | (-0.83,1.42) | (-0.68,0.96) | (-0.25,1.16) | (-0.8,1.1) |
| 0.17 | -0.13 | -0.41 | -0.32 | -0.05 | C-NSA | 0.44 | 0.33 | 0.2 | 0.52 | 0.2 |
| (-0.59,0.93) | (-0.74,0.49) | (-1.25,0.45) | (-1.25,0.59) | (-0.81,0.7) |  | (-0.88,1.77) | (-0.93,1.6) | (-0.75,1.17) | (-0.3,1.33) | (-1.03,1.48) |
| 0.62 | 0.32 | 0.04 | 0.13 | 0.4 | 0.45 | TCA | -0.11 | -0.24 | 0.07 | -0.24 |
| (-0.52,1.76) | (-0.79,1.43) | (-1.18,1.23) | (-1.11,1.37) | (-0.62,1.41) | (-0.8,1.73) |  | (-1.64,1.38) | (-1.58,1.09) | (-1.22,1.32) | (-1.63,1.17) |
| 0.49 | 0.19 | -0.09 | 0 | 0.27 | 0.33 | -0.13 | TAP | -0.13 | 0.18 | -0.13 |
| (-0.64,1.64) | (-0.88,1.26) | (-1.29,1.12) | (-0.81,0.83) | (-0.83,1.37) | (-0.9,1.56) | (-1.6,1.37) |  | (-1.24,0.98) | (-1.03,1.38) | (-1.58,1.37) |
| 0.36 | 0.06 | -0.22 | -0.13 | 0.14 | 0.19 | -0.26 | -0.13 | ACE+OPI | 0.32 | 0.01 |
| (-0.48,1.18) | (-0.66,0.77) | (-1.16,0.68) | (-0.86,0.59) | (-0.66,0.93) | (-0.75,1.12) | (-1.55,1.02) | (-1.22,0.96) |  | (-0.58,1.2) | (-1.26,1.29) |
| 0.68 | 0.38 | 0.1 | 0.18 | 0.46 | 0.51 | 0.06 | 0.18 | 0.32 | NSA+SMR | -0.31 |
| (-0.01,1.34) | (-0.13,0.87) | (-0.69,0.86) | (-0.68,1.04) | (-0.24,1.12) | (-0.3,1.29) | (-1.18,1.27) | (-1.02,1.35) | (-0.54,1.19) |  | (-1.48,0.88) |
| 0.38 | 0.08 | -0.2 | -0.12 | 0.16 | 0.21 | -0.24 | -0.12 | 0.02 | -0.3 | ALG |
| (-0.7,1.45) | (-0.96,1.09) | (-1.36,0.94) | (-1.29,1.06) | (-0.77,1.05) | (-1,1.38) | (-1.61,1.11) | (-1.55,1.31) | (-1.2,1.23) | (-1.44,0.84) |  |

Table S21. Acute LBP, Function Improvement

| PLA | 0.19 | 0.36 | -0.08 | 0.52 | 0.28 | 0.36 | 0.32 | 0.45 |
| --- | --- | --- | --- | --- | --- | --- | --- | --- |
|  | (-0.48,0.86) | (-0.32,1.02) | (-0.69,0.56) | (-0.26,1.36) | (-0.68,1.24) | (-0.61,1.35) | (-0.45,1.13) | (-0.47,1.35) |
| 0.27 | SMR | 0.17 | -0.27 | 0.32 | 0.09 | 0.16 | 0.13 | 0.25 |
| (-0.07,0.59) |  | (-0.78,1.09) | (-1.19,0.64) | (-0.69,1.39) | (-1.1,1.27) | (-1.03,1.37) | (-0.9,1.15) | (-0.88,1.39) |
| 0.37 | 0.1 | NSA | -0.44 | 0.15 | -0.08 | 0 | -0.04 | 0.09 |
| (-0.13,0.87) | (-0.49,0.71) |  | (-1.35,0.49) | (-0.29,0.66) | (-0.8,0.61) | (-0.71,0.72) | (-0.43,0.36) | (-0.52,0.7) |
| -0.08 | -0.34 | -0.45 | ACE | 0.59 | 0.36 | 0.44 | 0.4 | 0.53 |
| (-0.53,0.38) | (-0.92,0.22) | (-1.12,0.23) |  | (-0.42,1.65) | (-0.82,1.49) | (-0.71,1.59) | (-0.6,1.39) | (-0.59,1.59) |
| 0.51 | 0.25 | 0.14 | 0.59 | C-NSA | -0.23 | -0.16 | -0.19 | -0.07 |
| (-0.08,1.17) | (-0.42,0.99) | (-0.19,0.54) | (-0.17,1.41) |  | (-1.13,0.58) | (-1.04,0.68) | (-0.82,0.4) | (-0.86,0.66) |
| 0.29 | 0.03 | -0.08 | 0.37 | -0.22 | NSA+ACE | 0.08 | 0.04 | 0.17 |
| (-0.46,1.05) | (-0.81,0.84) | (-0.63,0.49) | (-0.5,1.24) | (-0.92,0.43) |  | (-0.91,1.1) | (-0.77,0.87) | (-0.75,1.1) |
| 0.36 | 0.1 | 0 | 0.44 | -0.15 | 0.07 | NSA+DIA | -0.03 | 0.09 |
| (-0.39,1.12) | (-0.74,0.92) | (-0.57,0.54) | (-0.44,1.31) | (-0.85,0.49) | (-0.75,0.87) |  | (-0.85,0.77) | (-0.86,1.01) |
| 0.33 | 0.06 | -0.04 | 0.41 | -0.18 | 0.04 | -0.03 | NSA+SMR | 0.12 |
| (-0.26,0.92) | (-0.62,0.74) | (-0.34,0.25) | (-0.33,1.16) | (-0.69,0.25) | (-0.61,0.68) | (-0.66,0.62) |  | (-0.5,0.74) |
| 0.46 | 0.19 | 0.09 | 0.54 | -0.05 | 0.17 | 0.09 | 0.13 | NSA+OPI+ACE |
| (-0.22,1.13) | (-0.57,0.95) | (-0.37,0.55) | (-0.28,1.37) | (-0.67,0.5) | (-0.56,0.9) | (-0.62,0.84) | (-0.32,0.59) |  |

Table S22. Chronic LBP, Pain Intensity

| PLA | 0.18 | **0.53** | **0.71** | 0.16 | **0.43** | -0.56 | 0.16 | 0.38 | 0.23 | **0.55** | **0.4** | **0.39** | **1.08** | -0.08 | 0.33 |
| --- | --- | --- | --- | --- | --- | --- | --- | --- | --- | --- | --- | --- | --- | --- | --- |
|  | (-0.05,0.41) | **(0.4,0.67)** | **(0.03,1.39)** | (-0.17,0.5) | **(0.17,0.7)** | (-1.25,0.12) | (-0.05,0.37) | (-0.27,1.03) | (-0.24,0.72) | **(0.31,0.79)** | **(0.1,0.71)** | **(0.19,0.59)** | **(0.4,1.76)** | (-0.51,0.34) | (-0.06,0.73) |
| **0.26** | NSA | **0.35** | 0.52 | -0.02 | 0.25 | **-0.75** | -0.02 | 0.2 | 0.05 | **0.36** | 0.22 | 0.21 | **0.9** | -0.27 | 0.15 |
| **(0.05,0.47)** |  | **(0.08,0.62)** | (-0.19,1.26) | (-0.43,0.39) | (-0.1,0.61) | **(-1.46,-0.05)** | (-0.29,0.25) | (-0.49,0.88) | (-0.47,0.57) | **(0.04,0.7)** | (-0.07,0.52) | (-0.1,0.51) | **(0.19,1.6)** | (-0.69,0.15) | (-0.3,0.6) |
| **0.51** | **0.25** | OPI | 0.18 | **-0.37** | -0.1 | **-1.09** | **-0.37** | -0.15 | -0.3 | 0.02 | -0.12 | -0.14 | 0.55 | **-0.61** | -0.2 |
| **(0.38,0.64)** | **(0.01,0.5)** |  | (-0.52,0.88) | **(-0.73,-0.01)** | (-0.37,0.17) | **(-1.8,-0.39)** | **(-0.63,-0.12)** | (-0.81,0.51) | (-0.8,0.2) | (-0.27,0.29) | (-0.47,0.21) | (-0.39,0.1) | (-0.14,1.25) | **(-1.06,-0.17)** | (-0.61,0.21) |
| **0.45** | 0.2 | -0.05 | TCA | -0.55 | -0.28 | **-1.27** | -0.55 | -0.33 | -0.48 | -0.16 | -0.3 | -0.32 | 0.37 | -0.79 | -0.38 |
| **(0.07,0.84)** | (-0.24,0.63) | (-0.46,0.34) |  | (-1.16,0.06) | (-1.01,0.47) | **(-2.24,-0.31)** | (-1.27,0.17) | (-0.67,0.01) | (-1.31,0.37) | (-0.89,0.57) | (-1.07,0.45) | (-1.01,0.37) | (-0.6,1.37) | (-1.6,0.02) | (-1.17,0.4) |
| 0.1 | -0.16 | **-0.41** | -0.35 | SSI | 0.27 | -0.73 | 0 | 0.22 | 0.07 | 0.39 | 0.24 | 0.23 | **0.92** | -0.24 | 0.17 |
| (-0.21,0.41) | (-0.53,0.22) | **(-0.75,-0.08)** | (-0.77,0.06) |  | (-0.16,0.7) | (-1.49,0.05) | (-0.4,0.4) | (-0.34,0.78) | (-0.52,0.67) | (-0.03,0.8) | (-0.21,0.7) | (-0.13,0.58) | **(0.17,1.68)** | (-0.78,0.3) | (-0.34,0.68) |
| **0.42** | 0.17 | -0.08 | -0.03 | 0.32 | TAP | **-1** | -0.27 | -0.05 | -0.2 | 0.12 | -0.03 | -0.04 | 0.65 | **-0.51** | -0.1 |
| **(0.16,0.69)** | (-0.17,0.5) | (-0.35,0.18) | (-0.49,0.43) | (-0.07,0.73) |  | **(-1.73,-0.26)** | (-0.62,0.06) | (-0.75,0.65) | (-0.75,0.34) | (-0.25,0.48) | (-0.44,0.38) | (-0.38,0.28) | (-0.08,1.38) | **(-1.02,-0.02)** | (-0.57,0.37) |
| -0.52 | **-0.77** | **-1.02** | **-0.97** | -0.62 | **-0.94** | ACE | **0.72** | 0.95 | 0.8 | **1.11** | **0.97** | **0.96** | **1.65** | 0.48 | **0.9** |
| (-1.2,0.17) | **(-1.48,-0.07)** | **(-1.71,-0.34)** | **(-1.74,-0.21)** | (-1.37,0.13) | **(-1.66,-0.21)** |  | **(0.07,1.39)** | (-0.01,1.89) | (-0.02,1.61) | **(0.4,1.84)** | **(0.24,1.72)** | **(0.24,1.66)** | **(0.72,2.56)** | (-0.32,1.29) | **(0.12,1.69)** |
| **0.21** | -0.05 | **-0.3** | -0.24 | 0.11 | -0.21 | **0.73** | C-NSA | 0.22 | 0.07 | **0.39** | 0.25 | 0.23 | **0.92** | -0.24 | 0.17 |
| **(0.03,0.39)** | (-0.29,0.2) | **(-0.53,-0.08)** | (-0.65,0.17) | (-0.25,0.47) | (-0.54,0.1) | **(0.07,1.38)** |  | (-0.46,0.91) | (-0.4,0.56) | **(0.1,0.69)** | (-0.11,0.6) | (-0.06,0.52) | **(0.27,1.59)** | (-0.71,0.21) | (-0.28,0.62) |
| 0.17 | -0.09 | -0.34 | -0.28 | 0.07 | -0.25 | 0.69 | -0.04 | ANC | -0.15 | 0.17 | 0.02 | 0.01 | 0.7 | -0.46 | -0.05 |
| (-0.28,0.62) | (-0.58,0.41) | (-0.8,0.13) | (-0.61,0.04) | (-0.37,0.51) | (-0.77,0.26) | (-0.11,1.49) | (-0.52,0.43) |  | (-0.95,0.67) | (-0.54,0.86) | (-0.69,0.75) | (-0.65,0.67) | (-0.23,1.65) | (-1.25,0.32) | (-0.81,0.7) |
| 0.16 | -0.09 | -0.34 | -0.29 | 0.06 | -0.26 | 0.68 | -0.05 | -0.01 | GMA | 0.32 | 0.17 | 0.16 | **0.85** | -0.31 | 0.1 |
| (-0.22,0.55) | (-0.52,0.34) | (-0.75,0.06) | (-0.66,0.07) | (-0.39,0.51) | (-0.72,0.21) | (-0.08,1.45) | (-0.45,0.36) | (-0.47,0.46) |  | (-0.23,0.85) | (-0.41,0.74) | (-0.37,0.68) | **(0.19,1.53)** | (-0.95,0.32) | (-0.52,0.73) |
| **0.55** | 0.3 | 0.05 | 0.1 | **0.45** | 0.13 | **1.07** | **0.34** | 0.38 | 0.39 | ACE+TRA | -0.14 | -0.16 | 0.54 | **-0.63** | -0.21 |
| **(0.32,0.79)** | (-0.01,0.61) | (-0.22,0.31) | (-0.36,0.55) | **(0.07,0.84)** | (-0.22,0.48) | **(0.36,1.79)** | **(0.07,0.63)** | (-0.14,0.89) | (-0.06,0.84) |  | (-0.54,0.25) | (-0.47,0.16) | (-0.17,1.23) | **(-1.12,-0.14)** | (-0.68,0.25) |
| **0.45** | 0.19 | -0.06 | -0.01 | 0.35 | 0.02 | **0.96** | 0.24 | 0.28 | 0.28 | -0.11 | TAN | -0.01 | 0.68 | -0.49 | -0.07 |
| **(0.15,0.75)** | (-0.1,0.48) | (-0.39,0.27) | (-0.49,0.49) | (-0.09,0.78) | (-0.38,0.42) | **(0.22,1.7)** | (-0.1,0.58) | (-0.27,0.82) | (-0.2,0.77) | (-0.49,0.28) |  | (-0.39,0.35) | (-0.05,1.42) | (-0.99,0.01) | (-0.57,0.43) |
| **0.39** | 0.13 | -0.12 | -0.07 | 0.29 | -0.04 | **0.9** | 0.18 | 0.22 | 0.22 | -0.17 | -0.06 | DUL | 0.69 | **-0.47** | -0.06 |
| **(0.19,0.58)** | (-0.16,0.41) | (-0.36,0.11) | (-0.49,0.36) | (-0.05,0.62) | (-0.37,0.29) | **(0.19,1.61)** | (-0.09,0.44) | (-0.27,0.7) | (-0.21,0.64) | (-0.48,0.14) | (-0.43,0.3) |  | (-0.01,1.4) | **(-0.94,-0.01)** | (-0.5,0.38) |
| **1.09** | **0.83** | 0.58 | 0.63 | **0.99** | 0.66 | **1.6** | **0.88** | **0.92** | **0.92** | 0.53 | 0.64 | **0.7** | C-NSA+GMA | **-1.16** | -0.75 |
| **(0.43,1.74)** | **(0.15,1.5)** | (-0.09,1.23) | (-0.08,1.33) | **(0.28,1.7)** | (-0.04,1.36) | **(0.68,2.51)** | **(0.23,1.51)** | **(0.17,1.65)** | **(0.27,1.58)** | (-0.17,1.22) | (-0.08,1.34) | **(0.02,1.38)** |  | **(-1.97,-0.37)** | (-1.54,0.04) |
| -0.05 | -0.31 | **-0.56** | -0.51 | -0.15 | -0.47 | 0.47 | -0.26 | -0.22 | -0.21 | **-0.6** | -0.5 | -0.44 | **-1.14** | ANE | 0.42 |
| (-0.47,0.38) | (-0.72,0.12) | **(-1,-0.11)** | (-1.07,0.07) | (-0.67,0.39) | (-0.97,0.03) | (-0.33,1.27) | (-0.71,0.2) | (-0.84,0.39) | (-0.78,0.36) | **(-1.08,-0.12)** | (-0.99,0.01) | (-0.89,0.04) | **(-1.91,-0.36)** |  | (-0.17,0.99) |
| 0.33 | 0.08 | -0.17 | -0.12 | 0.23 | -0.09 | **0.85** | 0.12 | 0.16 | 0.17 | -0.22 | -0.11 | -0.05 | -0.75 | 0.38 | BUP |
| (-0.05,0.74) | (-0.37,0.54) | (-0.59,0.25) | (-0.68,0.43) | (-0.26,0.73) | (-0.56,0.39) | **(0.06,1.64)** | (-0.3,0.56) | (-0.44,0.76) | (-0.38,0.71) | (-0.67,0.24) | (-0.62,0.39) | (-0.49,0.4) | (-1.5,0.01) | (-0.19,0.97) |  |

Table S23. Chronic LBP, Function Improvement

| PLA | 0.21  (-0.46,0.87) | 0.36  (-0.31,1.03) | **0.93**  **(0.36,1.51)** | 0.29  (-0.36,0.94) | 0.61  (-0.55,1.76) | 0.45  (-0.44,1.32) | 0.16  (-0.75,1.07) | 0.14  (-1.18,1.46) | -0.44  (-1.99,1.06) | 0.08  (-1.09,1.27) | 0.12  (-1.18,1.42) |
| --- | --- | --- | --- | --- | --- | --- | --- | --- | --- | --- | --- |
| 0.29  (-0.43,1.01) | NSA | 0.15  (-0.68,0.99) | 0.72  (-0.16,1.61) | 0.08  (-0.84,1.01) | 0.4  (-0.96,1.71) | 0.23  (-0.64,1.1) | -0.05  (-1.18,1.1) | -0.07  (-1.54,1.43) | -0.65  (-2.24,0.91) | -0.13  (-1.31,1.04) | -0.09  (-1.56,1.38) |
| **0.64**  **(0,1.27)** | 0.35  (-0.49,1.21) | C-NSA | 0.57  (-0.32,1.45) | -0.07  (-1.01,0.86) | 0.25  (-1.07,1.57) | 0.08  (-0.96,1.13) | -0.2  (-1.33,0.92) | -0.22  (-1.7,1.24) | -0.81  (-2.16,0.57) | -0.28  (-1.6,1.04) | -0.24  (-1.69,1.2) |
| **0.93**  **(0.3,1.56)** | 0.64  (-0.33,1.6) | 0.29  (-0.6,1.18) | OPI | -0.64  (-1.51,0.23) | -0.32  (-1.5,0.83) | -0.49  (-1.53,0.56) | -0.77  (-1.85,0.29) | -0.79  (-2.23,0.67) | -1.38  (-3.03,0.25) | -0.85  (-2.17,0.45) | -0.81  (-2.19,0.6) |
| 0.29  (-0.4,0.99) | 0  (-1,0.99) | -0.35  (-1.28,0.6) | -0.64  (-1.58,0.3) | TRA+ACE | 0.32  (-0.98,1.63) | 0.16  (-0.94,1.23) | -0.13  (-1.27,1) | -0.15  (-1.62,1.34) | -0.73  (-2.4,0.92) | -0.21  (-1.54,1.15) | -0.17  (-1.61,1.27) |
| 0.6  (-0.64,1.84) | 0.31  (-1.13,1.76) | -0.04  (-1.43,1.38) | -0.33  (-1.55,0.91) | 0.31  (-1.11,1.74) | TAP | -0.17  (-1.59,1.28) | -0.45  (-1.91,1.03) | -0.47  (-2.25,1.27) | -1.06  (-2.97,0.82) | -0.53  (-2.15,1.12) | -0.49  (-2.23,1.22) |
| 0.5  (-0.45,1.43) | 0.21  (-0.73,1.14) | -0.14  (-1.22,0.96) | -0.43  (-1.55,0.68) | 0.21  (-0.95,1.38) | -0.1  (-1.7,1.43) | TAN | -0.29  (-1.54,0.96) | -0.3  (-1.88,1.28) | -0.89  (-2.6,0.83) | -0.36  (-1.74,1.03) | -0.33  (-1.87,1.24) |
| 0.15  (-0.83,1.13) | -0.14  (-1.34,1.06) | -0.48  (-1.64,0.67) | -0.78  (-1.95,0.4) | -0.13  (-1.32,1.05) | -0.45  (-2.04,1.14) | -0.35  (-1.69,0.96) | DUL | -0.02  (-1.63,1.6) | -0.6  (-2.38,1.16) | -0.08  (-1.58,1.43) | -0.04  (-1.6,1.55) |
| 0.16  (-1.26,1.6) | -0.13  (-1.72,1.46) | -0.48  (-2.02,1.09) | -0.77  (-2.3,0.82) | -0.13  (-1.7,1.46) | -0.44  (-2.31,1.44) | -0.34  (-2.03,1.4) | 0  (-1.71,1.76) | SSI | -0.59  (-2.62,1.41) | -0.06  (-1.83,1.7) | -0.02  (-1.89,1.84) |
| -0.15  (-1.72,1.43) | -0.44  (-2.09,1.25) | -0.79  (-2.23,0.67) | -1.08  (-2.77,0.66) | -0.44  (-2.14,1.3) | -0.75  (-2.74,1.27) | -0.65  (-2.46,1.15) | -0.3  (-2.12,1.57) | -0.31  (-2.41,1.84) | ACE | 0.53  (-1.37,2.41) | 0.56  (-1.41,2.55) |
| 0.13  (-1.16,1.4) | -0.16  (-1.45,1.09) | -0.51  (-1.9,0.89) | -0.8  (-2.19,0.62) | -0.16  (-1.62,1.27) | -0.47  (-2.24,1.27) | -0.37  (-1.89,1.1) | -0.03  (-1.64,1.58) | -0.03  (-1.91,1.86) | 0.28  (-1.77,2.31) | ANE | 0.04  (-1.7,1.78) |
| 0.11  (-1.25,1.49) | -0.18  (-1.73,1.35) | -0.53  (-2.01,0.99) | -0.82  (-2.31,0.69) | -0.18  (-1.7,1.36) | -0.49  (-2.36,1.35) | -0.39  (-2.03,1.26) | -0.04  (-1.74,1.65) | -0.05  (-2.04,1.91) | 0.26  (-1.86,2.33) | -0.01  (-1.88,1.86) | BUP |

Table S24. Radicular LBP, Pain Intensity

| PLA | 0  (-0.38,0.39) | 0.01  (-0.51,0.56) | 0.48  (-0.98,1.96) | 0.19  (-0.19,0.59) | 0.78  (-0.08,1.62) | 0.85  (-0.79,2.48) | **0.92**  **(0.36,1.52)** | 0.68  (-0.82,2.19) | 0.32  (-0.6,1.27) | 1.22  (-0.11,2.59) |
| --- | --- | --- | --- | --- | --- | --- | --- | --- | --- | --- |
| -0.01  (-0.41,0.38) | NSA | 0.01  (-0.65,0.66) | 0.48  (-1.03,1.99) | 0.18  (-0.29,0.67) | 0.78  (-0.16,1.71) | 0.84  (-0.82,2.52) | **0.92**  **(0.45,1.4)** | 0.67  (-0.84,2.2) | 0.32  (-0.66,1.29) | 1.22  (-0.17,2.64) |
| 0.17  (-0.3,0.67) | 0.17  (-0.41,0.81) | COR | 0.47  (-1.1,2.03) | 0.17  (-0.49,0.86) | 0.77  (-0.24,1.78) | 0.83  (-0.9,2.55) | **0.91**  **(0.13,1.73)** | 0.66  (-0.92,2.24) | 0.31  (-0.78,1.39) | 1.21  (-0.24,2.67) |
| 0.41  (-1.05,1.93) | 0.42  (-1.08,2) | 0.25  (-1.3,1.82) | OPI | -0.29  (-1.74,1.13) | 0.3  (-1.38,1.98) | 0.37  (-0.37,1.09) | 0.44  (-1.11,2.01) | 0.2  (-0.96,1.36) | -0.16  (-1.32,1.01) | 0.74  (-0.77,2.24) |
| 0.11  (-0.26,0.49) | 0.12  (-0.36,0.6) | -0.06  (-0.64,0.47) | -0.31  (-1.78,1.13) | GMA | 0.59  (-0.35,1.52) | 0.66  (-0.92,2.28) | **0.74**  **(0.12,1.35)** | 0.49  (-0.95,1.95) | 0.14  (-0.72,0.97) | 1.04  (-0.29,2.34) |
| 0.78  (-0.11,1.65) | 0.79  (-0.17,1.74) | 0.62  (-0.39,1.61) | 0.37  (-1.39,2.08) | 0.67  (-0.28,1.62) | DUL | 0.07  (-1.78,1.89) | 0.14  (-0.87,1.17) | -0.1  (-1.84,1.62) | -0.46  (-1.71,0.8) | 0.44  (-1.13,2.02) |
| 0.79  (-0.88,2.46) | 0.79  (-0.88,2.52) | 0.62  (-1.08,2.36) | 0.37  (-0.37,1.12) | 0.68  (-0.94,2.33) | 0  (-1.89,1.89) | TAP | 0.07  (-1.63,1.8) | -0.17  (-1.52,1.18) | -0.52  (-1.88,0.85) | 0.38  (-1.26,2.07) |
| **0.9**  **(0.32,1.5)** | **0.91**  **(0.42,1.42)** | 0.73  (-0.03,1.48) | 0.49  (-1.12,2.06) | **0.79**  **(0.17,1.43)** | 0.12  (-0.93,1.19) | 0.11  (-1.66,1.87) | NSA+GMA | -0.24  (-1.83,1.32) | -0.6  (-1.66,0.44) | 0.3  (-1.14,1.73) |
| 0.6  (-0.89,2.14) | 0.61  (-0.92,2.19) | 0.44  (-1.15,2) | 0.19  (-0.97,1.35) | 0.5  (-0.95,1.96) | -0.18  (-1.9,1.62) | -0.18  (-1.53,1.18) | -0.3  (-1.87,1.31) | TCA | -0.36  (-1.55,0.84) | 0.54  (-1,2.09) |
| 0.25  (-0.66,1.21) | 0.26  (-0.7,1.27) | 0.08  (-0.94,1.1) | -0.16  (-1.36,0.97) | 0.14  (-0.69,1.01) | -0.53  (-1.81,0.79) | -0.53  (-1.93,0.84) | -0.65  (-1.72,0.44) | -0.35  (-1.55,0.86) | ANC | 0.9  (-0.08,1.88) |
| 1.15  (-0.18,2.48) | 1.15  (-0.22,2.54) | 0.98  (-0.42,2.37) | 0.73  (-0.82,2.25) | 1.04  (-0.26,2.33) | 0.36  (-1.23,1.96) | 0.36  (-1.39,2.06) | 0.25  (-1.22,1.69) | 0.54  (-1.07,2.09) | 0.9  (-0.1,1.87) | ANE |

Table S25. Radicular LBP, Function Improvement

| PLA | 0.12  (-0.71,0.89) | -0.2  (-1.34,0.93) | 0.38  (-1.7,2.5) | 0.27  (-1.87,2.39) | 0.1  (-1.52,1.72) | 0.23  (-1.81,2.28) |
| --- | --- | --- | --- | --- | --- | --- |
| 0.15  (-0.48,0.69) | COR | -0.32  (-1.66,1.1) | 0.26  (-1.94,2.58) | 0.15  (-2.17,2.41) | -0.02  (-1.77,1.79) | 0.1  (-2.03,2.32) |
| -0.2  (-0.94,0.51) | -0.35  (-1.11,0.44) | GMA | 0.57  (-1.18,2.39) | 0.47  (-1.4,2.31) | 0.3  (-0.9,1.5) | 0.42  (-1.31,2.17) |
| 0.37  (-1.35,2.05) | 0.22  (-1.5,1.96) | 0.57  (-1,2.11) | OPI | -0.1  (-1.5,1.32) | -0.28  (-1.66,1.1) | -0.15  (-2.01,1.77) |
| 0.27  (-1.54,1.97) | 0.12  (-1.68,1.9) | 0.47  (-1.16,2.06) | -0.1  (-1.37,1.13) | TCA | -0.17  (-1.58,1.29) | -0.05  (-1.94,1.85) |
| 0.09  (-1.13,1.31) | -0.06  (-1.28,1.22) | 0.3  (-0.68,1.26) | -0.28  (-1.51,0.95) | -0.17  (-1.44,1.12) | ANC | 0.13  (-1.16,1.39) |
| 0.23  (-1.41,1.85) | 0.08  (-1.55,1.75) | 0.43  (-1.05,1.87) | -0.14  (-1.8,1.48) | -0.04  (-1.68,1.64) | 0.14  (-0.97,1.2) | ANE |

**12. References of the Included Studies**

1. Amlie E, Weber H, Holme I. Treatment of acute low-back pain with piroxicam: results of a double-blind placebo-controlled trial. *Spine* **12**, 473-476 (1987).

2. Atkinson JH*, et al.* Effects of noradrenergic and serotonergic antidepressants on chronic low back pain intensity. *Pain* **83**, 137-145 (1999).

3. Atkinson JH*, et al.* Efficacy of noradrenergic and serotonergic antidepressants in chronic back pain: a preliminary concentration-controlled trial. *Journal of clinical psychopharmacology* **27**, 135-142 (2007).

4. Atkinson JH*, et al.* A randomized controlled trial of gabapentin for chronic low back pain with and without a radiating component. *Pain* **157**, 1499-1507 (2016).

5. Baron R*, et al.* The efficacy and safety of pregabalin in the treatment of neuropathic pain associated with chronic lumbosacral radiculopathy. *Pain* **150**, 420-427 (2010).

6. Baron R*, et al.* Effectiveness of Tapentadol Prolonged Release (PR) Compared with Oxycodone/Naloxone PR for the Management of Severe Chronic Low Back Pain with a Neuropathic Component: A Randomized, Controlled, Open-Label, Phase 3b/4 Study. *Pain practice : the official journal of World Institute of Pain* **16**, 580-599 (2016).

7. Bedaiwi MK*, et al.* Clinical Efficacy of Celecoxib Compared to Acetaminophen in Chronic Nonspecific Low Back Pain: Results of a Randomized Controlled Trial. *Arthritis care & research* **68**, 845-852 (2016).

8. Berry H, Bloom B, Hamilton EB, Swinson DR. Naproxen sodium, diflunisal, and placebo in the treatment of chronic back pain. *Annals of the rheumatic diseases* **41**, 129-132 (1982).

9. Berry H, Hutchinson DR. A multicentre placebo-controlled study in general practice to evaluate the efficacy and safety of tizanidine in acute low-back pain. *The Journal of international medical research* **16**, 75-82 (1988).

10. Berry H, Hutchinson DR. Tizanidine and ibuprofen in acute low-back pain: results of a double-blind multicentre study in general practice. *The Journal of international medical research* **16**, 83-91 (1988).

11. Biondi D, Xiang J, Benson C, Etropolski M, Moskovitz B, Rauschkolb C. Tapentadol immediate release versus oxycodone immediate release for treatment of acute low back pain. *Pain physician* **16**, E237-246 (2013).

12. Birbara CA*, et al.* Treatment of chronic low back pain with etoricoxib, a new cyclo-oxygenase-2 selective inhibitor: improvement in pain and disability--a randomized, placebo-controlled, 3-month trial. *The journal of pain : official journal of the American Pain Society* **4**, 307-315 (2003).

13. Buynak R*, et al.* Efficacy and safety of tapentadol extended release for the management of chronic low back pain: results of a prospective, randomized, double-blind, placebo- and active-controlled Phase III study. *Expert opinion on pharmacotherapy* **11**, 1787-1804 (2010).

14. Chandanwale AS*, et al.* Evaluation of eperisone hydrochloride in the treatment of acute musculoskeletal spasm associated with low back pain: a randomized, double-blind, placebo-controlled trial. *Journal of postgraduate medicine* **57**, 278-285 (2011).

15. Christoph A, Eerdekens MH, Kok M, Volkers G, Freynhagen R. Cebranopadol, a novel first-in-class analgesic drug candidate: first experience in patients with chronic low back pain in a randomized clinical trial. *Pain* **158**, 1813-1824 (2017).

16. Chu LF*, et al.* Analgesic tolerance without demonstrable opioid-induced hyperalgesia: a double-blinded, randomized, placebo-controlled trial of sustained-release morphine for treatment of chronic nonradicular low-back pain. *Pain* **153**, 1583-1592 (2012).

17. Coats TL, Borenstein DG, Nangia NK, Brown MT. Effects of valdecoxib in the treatment of chronic low back pain: results of a randomized, placebo-controlled trial. *Clinical therapeutics* **26**, 1249-1260 (2004).

18. Dapas F*, et al.* Baclofen for the treatment of acute low-back syndrome. A double-blind comparison with placebo. *Spine* **10**, 345-349 (1985).

19. Dickens C, Jayson M, Sutton C, Creed F. The relationship between pain and depression in a trial using paroxetine in sufferers of chronic low back pain. *Psychosomatics* **41**, 490-499 (2000).

20. Dreiser RL, Le Parc JM, Vélicitat P, Lleu PL. Oral meloxicam is effective in acute sciatica: two randomised, double-blind trials versus placebo or diclofenac. *Inflammation research : official journal of the European Histamine Research Society [et al]* **50 Suppl 1**, S17-23 (2001).

21. Dreiser RL, Marty M, Ionescu E, Gold M, Liu JH. Relief of acute low back pain with diclofenac-K 12.5 mg tablets: a flexible dose, ibuprofen 200 mg and placebo-controlled clinical trial. *International journal of clinical pharmacology and therapeutics* **41**, 375-385 (2003).

22. Eken C, Serinken M, Elicabuk H, Uyanik E, Erdal M. Intravenous paracetamol versus dexketoprofen versus morphine in acute mechanical low back pain in the emergency department: a randomised double-blind controlled trial. *Emergency medicine journal : EMJ* **31**, 177-181 (2014).

23. Enomoto H, Sasaki N, Fujikoshi S, Yoshikawa A, Tsuji T, Takeshita K. Relationship Between Pain Alleviation and Disease-specific Health-related Quality of Life Measures in Patients With Chronic Low Back Pain Receiving Duloxetine: Exploratory Post Hoc Analysis of a Japanese Phase 3 Randomized Study. *Journal of the American Academy of Orthopaedic Surgeons Global research & reviews* **3**, (2019).

24. Friedman BW*, et al.* Naproxen With Cyclobenzaprine, Oxycodone/Acetaminophen, or Placebo for Treating Acute Low Back Pain: A Randomized Clinical Trial. *Jama* **314**, 1572-1580 (2015).

25. Friedman BW*, et al.* Diazepam Is No Better Than Placebo When Added to Naproxen for Acute Low Back Pain. *Annals of emergency medicine* **70**, 169-176.e161 (2017).

26. Friedman BW*, et al.* A Randomized, Double-Blind, Placebo-Controlled Trial of Naproxen With or Without Orphenadrine or Methocarbamol for Acute Low Back Pain. *Annals of emergency medicine* **71**, 348-356.e345 (2018).

27. Friedman BW*, et al.* A Randomized, Placebo-Controlled Trial of Ibuprofen Plus Metaxalone, Tizanidine, or Baclofen for Acute Low Back Pain. *Annals of emergency medicine* **74**, 512-520 (2019).

28. Friedman BW*, et al.* Ibuprofen Plus Acetaminophen Versus Ibuprofen Alone for Acute Low Back Pain: An Emergency Department-based Randomized Study. *Academic emergency medicine : official journal of the Society for Academic Emergency Medicine* **27**, 229-235 (2020).

29. Goldberg H*, et al.* Oral steroids for acute radiculopathy due to a herniated lumbar disk: a randomized clinical trial. *Jama* **313**, 1915-1923 (2015).

30. Goodkin K, Gullion CM, Agras WS. A randomized, double-blind, placebo-controlled trial of trazodone hydrochloride in chronic low back pain syndrome. *Journal of clinical psychopharmacology* **10**, 269-278 (1990).

31. Gurrell R*, et al.* A randomised, placebo-controlled clinical trial with the α2/3/5 subunit selective GABAA positive allosteric modulator PF-06372865 in patients with chronic low back pain. *Pain* **159**, 1742-1751 (2018).

32. Hale M, Khan A, Kutch M, Li S. Once-daily OROS hydromorphone ER compared with placebo in opioid-tolerant patients with chronic low back pain. *Current medical research and opinion* **26**, 1505-1518 (2010).

33. Hale ME, Ahdieh H, Ma T, Rauck R. Efficacy and safety of OPANA ER (oxymorphone extended release) for relief of moderate to severe chronic low back pain in opioid-experienced patients: a 12-week, randomized, double-blind, placebo-controlled study. *The journal of pain : official journal of the American Pain Society* **8**, 175-184 (2007).

34. Herrmann WA, Geertsen MS. Efficacy and safety of lornoxicam compared with placebo and diclofenac in acute sciatica/lumbo-sciatica: an analysis from a randomised, double-blind, multicentre, parallel-group study. *International journal of clinical practice* **63**, 1613-1621 (2009).

35. Innes GD, Croskerry P, Worthington J, Beveridge R, Jones D. Ketorolac versus acetaminophen-codeine in the emergency department treatment of acute low back pain. *The Journal of emergency medicine* **16**, 549-556 (1998).

36. Jenkins DG, Ebbutt AF, Evans CD. Tofranil in the treatment of low back pain. *The Journal of international medical research* **4**, 28-40 (1976).

37. Kalita J, Kohat AK, Misra UK, Bhoi SK. An open labeled randomized controlled trial of pregabalin versus amitriptyline in chronic low backache. *Journal of the neurological sciences* **342**, 127-132 (2014).

38. Katz N*, et al.* Efficacy and safety of rofecoxib in patients with chronic low back pain: results from two 4-week, randomized, placebo-controlled, parallel-group, double-blind trials. *Spine* **28**, 851-858; discussion 859 (2003).

39. Katz N*, et al.* A 12-week, randomized, placebo-controlled trial assessing the safety and efficacy of oxymorphone extended release for opioid-naive patients with chronic low back pain. *Current medical research and opinion* **23**, 117-128 (2007).

40. Katz N*, et al.* Efficacy and safety of tanezumab in the treatment of chronic low back pain. *Pain* **152**, 2248-2258 (2011)

41. Katz N, Kopecky EA, OʼConnor M, Brown RH, Fleming AB. A phase 3, multicenter, randomized, double-blind, placebo-controlled, safety, tolerability, and efficacy study of Xtampza ER in patients with moderate-to-severe chronic low back pain. *Pain* **156**, 2458-2467 (2015).

42. Kawamata M*, et al.* Efficacy and safety of controlled-release oxycodone for the management of moderate-to-severe chronic low back pain in Japan: results of an enriched enrollment randomized withdrawal study followed by an open-label extension study. *Journal of pain research* **12**, 363-375 (2019).

43. Ketenci A, Ozcan E, Karamursel S. Assessment of efficacy and psychomotor performances of thiocolchicoside and tizanidine in patients with acute low back pain. *International journal of clinical practice* **59**, 764-770 (2005).

44. Khoromi S, Patsalides A, Parada S, Salehi V, Meegan JM, Max MB. Topiramate in chronic lumbar radicular pain. *The journal of pain : official journal of the American Pain Society* **6**, 829-836 (2005).

45. Khoromi S, Cui L, Nackers L, Max MB. Morphine, nortriptyline and their combination vs. placebo in patients with chronic lumbar root pain. *Pain* **130**, 66-75 (2007).

46. Kivitz AJ*, et al.* Efficacy and safety of tanezumab versus naproxen in the treatment of chronic low back pain. *Pain* **154**, 1009-1021 (2013).

47. Ko S, Kim S, Kim J, Oh T. The Effectiveness of Oral Corticosteroids for Management of Lumbar Radiating Pain: Randomized, Controlled Trial Study. *Clinics in orthopedic surgery* **8**, 262-267 (2016).

48. Kopecky EA, Vaughn B, Lagasse S, O'Connor M. Tolerability, Safety, and Effectiveness of Oxycodone DETERx in Elderly Patients ≥65 Years of Age with Chronic Low Back Pain: A Randomized Controlled Trial. *Drugs & aging* **34**, 603-613 (2017).

49. Lee JH, Lee CS. A randomized, double-blind, placebo-controlled, parallel-group study to evaluate the efficacy and safety of the extended-release tramadol hydrochloride/acetaminophen fixed-dose combination tablet for the treatment of chronic low back pain. *Clinical therapeutics* **35**, 1830-1840 (2013).

50. Markman J, Meske DS, Kopecky EA, Vaughn B, O'Connor ML, Passik SD. Analgesic efficacy, safety, and tolerability of a long-acting abuse-deterrent formulation of oxycodone for moderate-to-severe chronic low back pain in subjects successfully switched from immediate-release oxycodone. *Journal of pain research* **11**, 2051-2059 (2018).

51. Markman J*, et al.* SUMMIT-07: a randomized trial of NKTR-181, a new molecular entity, full mu-opioid receptor agonist for chronic low-back pain. *Pain* **160**, 1374-1382 (2019).

52. Markman JD*, et al.* Double-blind, randomized, controlled, crossover trial of pregabalin for neurogenic claudication. *Neurology* **84**, 265-272 (2015).

53. Mathieson S*, et al.* Trial of Pregabalin for Acute and Chronic Sciatica. *The New England journal of medicine* **376**, 1111-1120 (2017).

54. Mazza M, Mazza O, Pazzaglia C, Padua L, Mazza S. Escitalopram 20 mg versus duloxetine 60 mg for the treatment of chronic low back pain. *Expert opinion on pharmacotherapy* **11**, 1049-1052 (2010).

55. Mccleane GJJPC. Gabapentin reduces chronic benign nociceptive pain: A double-blind, placebo-controlled cross-over study. **12**, 81-85 (2000).

56. Miki K*, et al.* Randomized open-label [corrected] non-inferiority trial of acetaminophen or loxoprofen for patients with acute low back pain. *Journal of orthopaedic science : official journal of the Japanese Orthopaedic Association* **23**, 483-487 (2018).

57. Nadler SF*, et al.* Continuous low-level heat wrap therapy provides more efficacy than Ibuprofen and acetaminophen for acute low back pain. *Spine* **27**, 1012-1017 (2002).

58. Nakashima H*, et al.* Is Pregabalin Effective Against Acute Lumbar Radicular Pain ? *Spine surgery and related research* **3**, 61-66 (2019).

59. Pallay RM*, et al.* Etoricoxib reduced pain and disability and improved quality of life in patients with chronic low back pain: a 3 month, randomized, controlled trial. *Scandinavian journal of rheumatology* **33**, 257-266 (2004).

60. Pareek A, Chandurkar N, Chandanwale AS, Ambade R, Gupta A, Bartakke G. Aceclofenac-tizanidine in the treatment of acute low back pain: a double-blind, double-dummy, randomized, multicentric, comparative study against aceclofenac alone. *European spine journal : official publication of the European Spine Society, the European Spinal Deformity Society, and the European Section of the Cervical Spine Research Society* **18**, 1836-1842 (2009).

61. Patel HD, Uppin RB, Naidu AR, Rao YR, Khandarkar S, Garg A. Efficacy and Safety of Combination of NSAIDs and Muscle Relaxants in the Management of Acute Low Back Pain. *Pain and therapy* **8**, 121-132 (2019).

62. Peloso PM, Fortin L, Beaulieu A, Kamin M, Rosenthal N. Analgesic efficacy and safety of tramadol/ acetaminophen combination tablets (Ultracet) in treatment of chronic low back pain: a multicenter, outpatient, randomized, double blind, placebo controlled trial. *The Journal of rheumatology* **31**, 2454-2463 (2004).

63. Perrot S, Krause D, Crozes P, Naïm C. Efficacy and tolerability of paracetamol/tramadol (325 mg/37.5 mg) combination treatment compared with tramadol (50 mg) monotherapy in patients with subacute low back pain: a multicenter, randomized, double-blind, parallel-group, 10-day treatment study. *Clinical therapeutics* **28**, 1592-1606 (2006).

64. Pohjolainen T, Jekunen A, Autio L, Vuorela H. Treatment of acute low back pain with the COX-2-selective anti-inflammatory drug nimesulide: results of a randomized, double-blind comparative trial versus ibuprofen. *Spine* **25**, 1579-1585 (2000).

65. Ralph L, Look M, Wheeler W, Sacks H. Double-blind, placebo-controlled trial of carisoprodol 250-mg tablets in the treatment of acute lower-back spasm. *Current medical research and opinion* **24**, 551-558 (2008).

66. Rauck RL*, et al.* Single-entity hydrocodone extended-release capsules in opioid-tolerant subjects with moderate-to-severe chronic low back pain: a randomized double-blind, placebo-controlled study. *Pain medicine (Malden, Mass)* **15**, 975-985 (2014).

67. Rauck RL, Potts J, Xiang Q, Tzanis E, Finn A. Efficacy and tolerability of buccal buprenorphine in opioid-naive patients with moderate to severe chronic low back pain. *Postgraduate medicine* **128**, 1-11 (2016).

68. Rodrigues LC, Natour J. A double-blind, randomized controlled, prospective trial assessing the effectiveness of oral corticoids in the treatment of symptomatic lumbar canal stenosis. *Journal of negative results in biomedicine* **13**, 13 (2014).

69. Romanò CL, Romanò D, Bonora C, Mineo G. Pregabalin, celecoxib, and their combination for treatment of chronic low-back pain. *Journal of orthopaedics and traumatology : official journal of the Italian Society of Orthopaedics and Traumatology* **10**, 185-191 (2009).

70. Ruoff GE, Rosenthal N, Jordan D, Karim R, Kamin M. Tramadol/acetaminophen combination tablets for the treatment of chronic lower back pain: a multicenter, randomized, double-blind, placebo-controlled outpatient study. *Clinical therapeutics* **25**, 1123-1141 (2003).

71. Schiphorst Preuper HR*, et al.* Do analgesics improve functioning in patients with chronic low back pain? An explorative triple-blinded RCT. *European spine journal : official publication of the European Spine Society, the European Spinal Deformity Society, and the European Section of the Cervical Spine Research Society* **23**, 800-806 (2014).

72. Schukro RP, Oehmke MJ, Geroldinger A, Heinze G, Kress HG, Pramhas S. Efficacy of Duloxetine in Chronic Low Back Pain with a Neuropathic Component: A Randomized, Double-blind, Placebo-controlled Crossover Trial. *Anesthesiology* **124**, 150-158 (2016).

73. Serfer GT, Wheeler WJ, Sacks HJ. Randomized, double-blind trial of carisoprodol 250 mg compared with placebo and carisoprodol 350 mg for the treatment of low back spasm. *Current medical research and opinion* **26**, 91-99 (2010).

74. Skljarevski V*, et al.* A double-blind, randomized trial of duloxetine versus placebo in the management of chronic low back pain. *European journal of neurology* **16**, 1041-1048 (2009).

75. Skljarevski V*, et al.* Duloxetine versus placebo in patients with chronic low back pain: a 12-week, fixed-dose, randomized, double-blind trial. *The journal of pain : official journal of the American Pain Society* **11**, 1282-1290 (2010).

76. Skljarevski V*, et al.* Efficacy and safety of duloxetine in patients with chronic low back pain. *Spine* **35**, E578-585 (2010).

77. Stein D, Peri T, Edelstein E, Elizur A, Floman Y. The efficacy of amitriptyline and acetaminophen in the management of acute low back pain. *Psychosomatics* **37**, 63-70 (1996).

78. Szpalski M, Hayez JP. Objective functional assessment of the efficacy of tenoxicam in the treatment of acute low back pain. A double-blind placebo-controlled study. *British journal of rheumatology* **33**, 74-78 (1994).

79. Tetsunaga T, Tetsunaga T, Tanaka M, Ozaki T. Efficacy of tramadol-acetaminophen tablets in low back pain patients with depression. *Journal of orthopaedic science : official journal of the Japanese Orthopaedic Association* **20**, 281-286 (2015).

80. Tsuji T, Itoh N, Ishida M, Ochiai T, Konno S. Response to duloxetine in chronic low back pain: exploratory post hoc analysis of a Japanese Phase III randomized study. *Journal of pain research* **10**, 2157-2168 (2017).

81. Urquhart DM*, et al.* Efficacy of Low-Dose Amitriptyline for Chronic Low Back Pain: A Randomized Clinical Trial. *JAMA internal medicine* **178**, 1474-1481 (2018).

82. Voicu VA*, et al.* Effect of a New Synergistic Combination of Low Doses of Acetylsalicylic Acid, Caffeine, Acetaminophen, and Chlorpheniramine in Acute Low Back Pain. *Frontiers in pharmacology* **10**, 607 (2019).

83. Weber H, Holme I, Amlie E. The natural course of acute sciatica with nerve root symptoms in a double-blind placebo-controlled trial evaluating the effect of piroxicam. *Spine* **18**, 1433-1438 (1993).

84. Wen W, Sitar S, Lynch SY, He E, Ripa SR. A multicenter, randomized, double-blind, placebo-controlled trial to assess the efficacy and safety of single-entity, once-daily hydrocodone tablets in patients with uncontrolled moderate to severe chronic low back pain. *Expert opinion on pharmacotherapy* **16**, 1593-1606 (2015).

85. Williams CM*, et al.* Efficacy of paracetamol for acute low-back pain: a double-blind, randomised controlled trial. *Lancet (London, England)* **384**, 1586-1596 (2014).

86. Ximenes A, Robles M, Sands G, Vinueza R. Valdecoxib is as efficacious as diclofenac in the treatment of acute low back pain. *The Clinical journal of pain* **23**, 244-250 (2007).

87. Yaksi A, Ozgönenel L, Ozgönenel B. The efficiency of gabapentin therapy in patients with lumbar spinal stenosis. *Spine* **32**, 939-942 (2007).

88. Zerbini C*, et al.* Efficacy of etoricoxib 60 mg/day and diclofenac 150 mg/day in reduction of pain and disability in patients with chronic low back pain: results of a 4-week, multinational, randomized, double-blind study. *Current medical research and opinion* **21**, 2037-2049 (2005).
